# Function, Structure, and Regulation of Nitrogen Fixation-like Metalloproteins for Nitrogen, Energy, Carbon, and Sulfur Metabolism

**DOI:** 10.1021/acs.chemrev.5c00922

**Published:** 2026-07-24

**Authors:** Justin A. North, Hannah S. Shafaat

**Affiliations:** † Department of Microbiology, 2647 The Ohio State University, Columbus, Ohio 43210, United States; ‡ Department of Chemistry and Biochemistry, 8783 University of California, Los Angeles, Los Angeles, California 90095, United States

## Abstract

Nitrogenases (N_2_ases) and nitrogen fixation-like (NFL) systems play distinct roles in nitrogen, carbon, sulfur, and energy metabolism based on their fundamental differences in structure and metallocofactor identity. As new NFL systems have recently been identified and characterized, striking parallels and differences compared to N_2_ase structure, catalysis, and regulation have emerged. NFL systems use metallocofactors that span from simple [4Fe-4S] clusters to complex clusters akin to FeMo-co, previously only thought to occur in N_2_ase. This review describes the present state of knowledge on the function, structure, catalytic mechanisms, and regulation of NFL systems that perform distinct biological roles across all three domains of life. Recent advancements in N_2_ase spectroscopic techniques for probing metallocofactor structure and electronic states guide current and future work on how each NFL system catalyzes its specific biological reaction(s). Key knowledge gaps and needed areas of research for uncovering the specific metallocofactors and structural motifs that are at the heart of NFL system reaction specificity, along with how these systems are regulated, are discussed.

## Graphical abstract

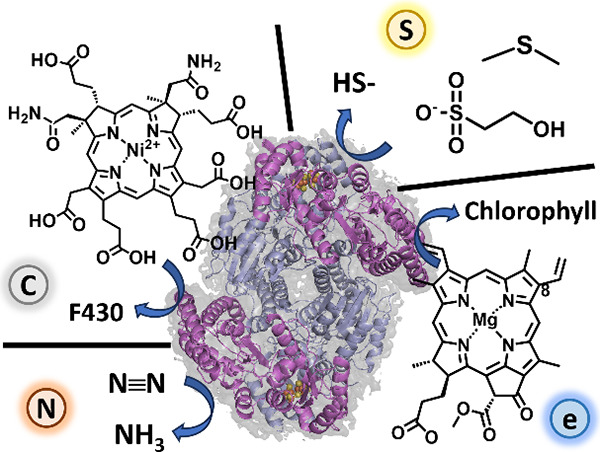

## 1 Introduction

Nitrogenases (N_2_ases) and nitrogen fixation-like (NFL) systems comprise a superfamily of enzymes involved in nitrogen, carbon, sulfur, and energy metabolism in diverse species ranging from prokaryotic bacteria and archaea to eukaryotic algae and plants. The three isoforms of N_2_ase, which are the quintessential biological catalysts for fixation of dinitrogen (N_2_) gas into ammonia, have been extensively studied for nearly 100 years. In the late 80s and early 90s, genes encoding proteins that looked like N_2_ases but were incapable of N_2_ fixation were found in the genomes of phototrophic bacteria, algae, mosses, and gymnosperm plants. These NFL sequences are called the dark-operative protochlorophyllide oxidoreductases (DPORs) and chlorophyllide oxidoreductases (CORs). DPORs and CORs catalyze specific C–C double bond reductions in the synthesis of chlorophyll (Chl) and bacteriochlorophyll (Bch) for light energy harvesting by photosystems. In the early 2010s, additional NFL sequences were discovered in the genomes of diverse bacteria and archaea. While many of these sequences remain of unknown or only hypothesized functions, several groups now have established roles. An NFL system for Ni^2+^-sirohydrochlorin *a*,*c*-diamide reduction called CfbCD catalyzes key steps in F430 cofactor biosynthesis (Cfb). F430 then serves as a cofactor of methyl-CoM reductase in methanogenic and methanotrophic archaea for carbon and energy metabolism. In bacteria, an NFL system named methylthio-alkane reductase (MAR) functions in the reductive cleavage of volatile organic sulfur compounds like dimethyl sulfide and 2-methylthioethanol for sulfur acquisition. Similarly, isethionate reductase (ISR) catalyzes cleavage of organic sulfonates like isethionate for sulfur acquisition, also in bacteria. In the realm of nitrogen metabolism, the NFL system designated as Nfa (for nitrogen fixation alternative) is distinct from N_2_ase and implicated in an alternative mechanism for N_2_ fixation. Further still, NFL systems waiting to be characterized are implicated in sulfur metabolism or the synthesis of diverse heterocyclic cofactors.

In section 2, we begin by introducing the biological role of N_2_ase and each NFL system of identified function, highlighting how the N_2_ase superfamily represents a diverse family of sequences, likely evolving from a common ancestor, for catalyzing the reduction of a surprising distribution of reactions. Notably, these reactions involve reduction of nitrogen–nitrogen, carbon–carbon, carbon–oxygen, and carbon–sulfur bonds, ranging from single to double to triple bonds, and in compounds ranging from diatomic gases to small organic molecules to large macrocyclic cofactors. There are many recent comprehensive reviews and perspectives on specific aspects of N_2_ase catalysis. These include N_2_ase catalytic mechanism;^1–6^ structure, enzymology, and reactivity of N_2_ase;^7–12^ electron transfer by N_2_ase components;^13,14^ assembly of N_2_ase metallocofactors,^15–19^ and spectroscopy of N_2_ase metallocofactors and mimics.^20–22^ As such, in section 3 we provide for the nonexpert an essential treatment of N_2_ase structure, catalysis, and metallocofactor identity, along with recent advances in spectroscopic methods that critically connect to the current and future understanding of NFL systems in sections 4–9. Specifically, sections 4–9 fully describe for each NFL system of known or proposed function the current state of knowledge on how their structure and metallocofactors work concertedly for electron transfer and ultimately substrate reduction. Further, as NFL systems are ultimately complex biological metalloprotein catalysts, metallocofactor maturation and the regulation of metalloprotein synthesis and activity are vital for their proper operation in the physiological context of a living cell. In each section, we detail fundamental progress on understanding of the transcriptional and post-translational regulatory mechanisms of NFL systems.

## 2 Nitrogenase and NFL Enzyme Function, Architecture, and Evolution

### 2.1 Biological and Biochemical Role of Nitrogenase in N_2_ Fixation

The primary function of N_2_ase is to catalyze the six-electron reduction of N_2_ into two ammonia (NH_3_) molecules. N_2_ase is found in the cytoplasm of certain bacteria and archaea, enabling them to function as diazotrophs, meaning that they can grow using N_2_ gas as a sole nitrogen source.^23–27^ Diazotrophic bacteria and archaea can be free living or form symbiotic and even endosymbiotic relationships with plants,^28–33^ fungi,^34–38^ and algae.^39–43^ Notably, N_2_ase has recently been observed to enable diazotrophic growth of some lineages of marine algae like *Braarudosphaera bigelowii*, which lack a recognizable (endo)­symbiont. This diazotrophic phenotype results from *B. bigelowii* apparently evolving its ancestral cyanobacterial endosymbiont (UCYN-A; *Candidatus atelocyanobacterium thalassa*) to the level of a N_2_ase-operating organelle called the nitroplast.^44–47^ N_2_ase from Rhizobiaceae family bacteria (i.e., *Teciglobus diatomicola*) may also be following the same trajectory for incorporation into marine diatoms.^32,41,48^ As such, species across all domains of life have now been observed to express and operate N_2_ase for biological N_2_ fixation. In total, free-living prokaryotes, (endo)­symbiotic prokaryotes, and eukaryotic organelle-like N_2_-fixing compartments contribute to 50–100 Tg (10^12^) fixed N_2_ per year in the global terrestrial ecosystem.^49–51^ Similarly, it is estimated that 135 Tg or more is fixed per year by organisms in marine ecosystems.^52^ Biological N_2_ fixation is on the same order of magnitude as the 140–150 Tg of nitrogen fixed industrially by the Haber–Bosch process for agricultural food production and chemical synthesis, representing 2% of the global energy consumption and 1–3% of the global anthropogenic CO_2_ budget.^53–55^ In contrast, lightning only accounts for 5–6 Tg of fixed nitrogen per year.^56,57^ Therefore, N_2_ fixation by N_2_ase accounts for approximately 50% of the biologically available nitrogen in the biosphere.

N_2_ases are two-component systems composed of a heterooligomeric catalytic complex (“component 1”) and a dimeric ATP-dependent N_2_ase reductase called the Fe protein (“component 2”) (Figure 1). Three distinct metallocofactors are involved in the electron transfer and substrate reduction processes of N_2_ase:

(a) the Fe protein [4Fe-4S] cluster is involved in transferring an electron to the catalytic component 1;
(b) the [8Fe-7S] P-cluster of component 1 accepts the electron from the Fe protein;
(c) the catalytic cluster (e.g., FeMo-co, also known as M-cluster) of component 1 accepts the electron from the P-cluster and transfers the electron to the substrate for reduction.

**Figure 1:**
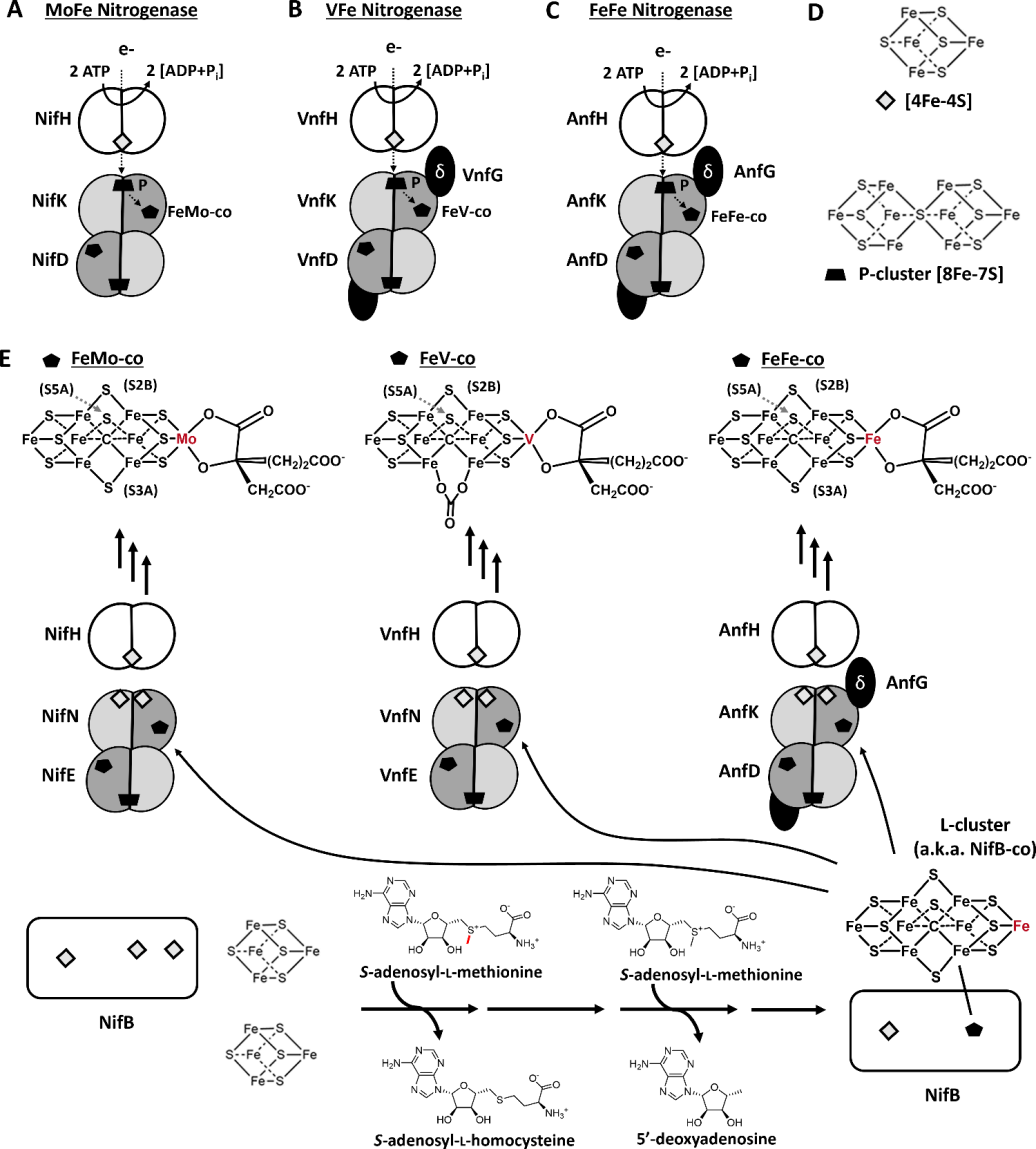
**The three isoforms of nitrogenase and their corresponding metallocofactors.** (A) Molybdenum (MoFe) N_2_ase is composed of the NifH dinitrogenase reductase Fe protein (component 2) and NifDK catalytic complex (component 1). (B) Vanadium (VFe) N_2_ase is composed of the VnfH dinitrogenase reductase Fe protein (component 2) and VnfDGK catalytic complex (component 1). (C) Iron-only (FeFe) N_2_ase is composed of the AnfH dinitrogenase reductase Fe protein (component 2) and AnfDGK catalytic complex (component 1). (D) Each Fe protein possesses a single [4Fe-4S] cluster at the homodimer interface for transferring an electron to the N_2_ase P-cluster, which resides at each of the two heterodimeric interfaces of the component 1 complex. (E) Structures of the catalytic cluster specific to each isoform of N_2_ase and their essential synthesis by NifB and NifEN or VnfEN chaperones. Additional proteins involved in metal delivery, homocitrate insertion, and fidelity are not shown.^18^ Note that in FeV-co, the belt-sulfur S3A is replaced by a coordinated carbonate ion.

There are three isoforms of N_2_ase, which are distinguished in part by the molybdenum, vanadium, or iron heterometal present in the catalytic cluster (Figure 1). Further, amino acid sequence and corresponding structural differences surrounding the catalytic cluster appear to govern the observed differences in substrate specificities for N_2_ versus alternative substrates across the three isoforms (reviewed in refs 3 and 8; see section 2.2). The molybdenum (MoFe) N_2_ase possess the catalytic FeMo-co, the vanadium (VFe) N_2_ase possesses the FeV-co, and the iron-only (FeFe) N_2_ase possesses the FeFe-co. In biological organisms, gene and corresponding protein components composing the MoFe N_2_ase are designated as Nif for “nitrogen fixation”. The Nif system Fe protein is the NifH_2_ homodimer and the catalytic component 1 is composed of NifDK in an α_2_β_2_ assembly.^58^ Components of the VFe N_2_ase system are designated as Vnf (for vanadium nitrogen fixation), with dimeric VnfH_2_ Fe protein and catalytic component 1 composed of VnfDGK in an α_2_β_2_δ_2_ assembly.^59,60^ Similarly, components of the FeFe N_2_ase system are designated as Anf (for alternative nitrogen fixation), with dimeric AnfH_2_ Fe protein and catalytic component I composed of AnfDGK also in an α_2_β_2_δ_2_ assembly.^61,62^

All N_2_ase Fe protein homologues are observed to possess a single [4Fe-4S] cluster at the dimeric NifH, VnfH, and AnfH dimer interface.^63^ Within the catalytic component 1 of N_2_ase, there are two [8Fe-7S] P-clusters, one coordinated at each of the αβ subunit interfaces (i.e., NifDK, VnfDK, AnfDK). Likewise, the catalytic FeMo-co, FeV-co, or FeFe-co is located near the P-cluster within each of the two N_2_ase α subunits (i.e., NifD, VnfD, AnfD) (Figure 1). For MoFe and FeFe N_2_ase, the respective FeMo-co and FeFe-co are a [7Fe-9S-C-X] metallocofactor with a *R-*homocitrate ligand and being X= Mo or Fe.^58,61^ In VFe N_2_ase, the FeV-co is a [7Fe-8S-CO_3_-C-V] metallocofactor with *R*-homocitrate ligand. Specifically, the CO_3_ carbonate ligand replaces sulfur S3A present in the other catalytic cofactor isoforms (Figure 1).^59^ The FeMo-co is synthesized by NifB and NifEN, the latter of which is an assembly scaffold that is a homologue of N_2_ase. NifB is a radical SAM enzyme that catalyzes multiple chemical transformations in series: (i) NifB joins two [4Fe-4S] “K” clusters, (ii) inserts and sequentially deprotonates the methyl group of *S-*adenosyl-l-methionine (SAM) to form a central μ^6^-carbide, and (iii) reduces sulfite and inserts the resultant sulfide to form the [8Fe-9S-C] L-cluster (a.k.a. NifB-co)^18,64–67^ (Figure 1). NifB subsequently transfers the L-cluster to NifEN where the homocitrate and Mo are added to form the mature FeMo-co.^18^ Similarly, FeV-co is assembled by NifB and VnfEN, the latter of which is also a homologue of N_2_ase. The mature metallocofactors are then transferred to their respective N_2_ase targets, NifDK and VnfDGK. For the FeFe N_2_ase, maturation of the L-cluster into FeFe-co occurs *in situ* on the AnfDGK scaffold.^18,68,69^ In the alternative VFe and FeFe N_2_ases, the additional VnfG or AnfG δ subunit binds to VnfD or AnfD, respectively, near where the Fe protein binds. These subunits are also thought to be involved in ensuring the stable or proper incorporation of the catalytic metallocofactor,^70–72^ and they have been implicated in recruiting the correct Fe protein to the VnfDK or AnfDK surface to perform electron transfer for catalysis.^62,73^

As part of the N_2_ase catalytic mechanism for strict proton-coupled N_2_ reduction, one equivalent of hydrogen is released through reductive elimination as an obligatory component of the mechanism (Figure 2).^1,74–78^ Notably, during the catalytic cycle, electrons transferred to the catalytic cofactor can also be lost through unproductive hydrogen release without subsequent reduction of N_2_ or alternative substrate. Under standard reaction conditions of 1 atm N_2_ and 1:1 ratio of Fe protein to N_2_ase catalytic complex, the MoFe N_2_ase is the most efficient at catalyzing N_2_ reduction to NH_3_ versus proton reduction to H_2_. The VFe and FeFe N_2_ases are increasingly less efficient, resulting in the following apparent reaction stoichiometries:

MoFe nitrogenase:

1 $$N_{2}+8e^{-}+8H^{+}+16\mathrm{MgATP}\to 2{\mathrm{NH}}_{3}+H_{2}+16\left[\mathrm{MgADP}+P_{i}\right]$$

VFe nitrogenase:

2 $$N_{2}+18e^{-}+18H^{+}+36\mathrm{MgATP}\to 2{\mathrm{NH}}_{3}+6H_{2}+36\left[\mathrm{MgADP}+P_{i}\right]$$

FeFe nitrogenase:

3 $$N_{2}+20e^{-}+20H^{+}+40\mathrm{MgATP}\to 2{\mathrm{NH}}_{3}+7H_{2}+40\left[\mathrm{MgADP}+P_{i}\right]$$

However, upon increasing the reaction electron flux by increasing the ratio of Fe protein to catalytic component and increasing the N_2_ partial pressure above 1 atm, all three isoforms converge to a common reaction scheme:^79^

4 $$N_{2}+8e^{-}+8H^{+}+16\mathrm{MgATP}\to 2{\mathrm{NH}}_{3}+H_{2}+16\left[\mathrm{MgADP}+P_{i}\right]$$

**Figure 2:**
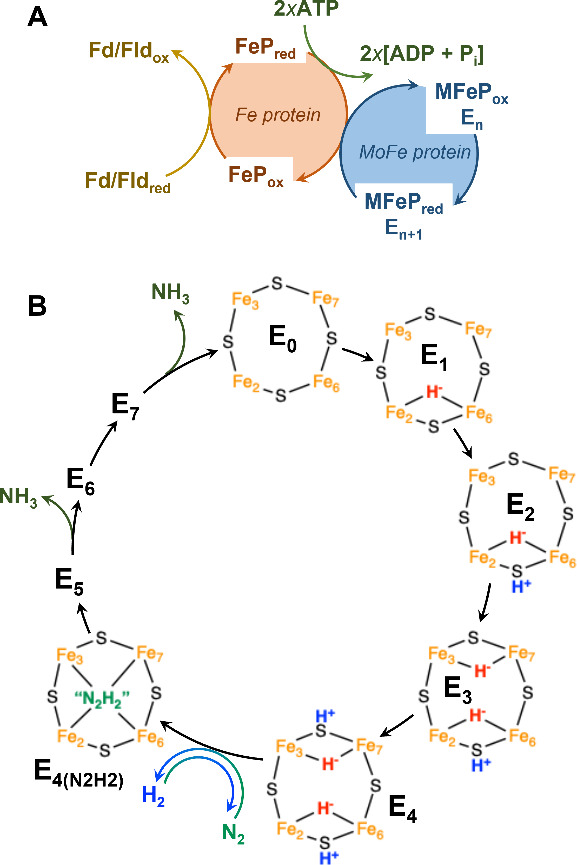
**Thorneley–Lowe model for electron transfer and catalysis by nitrogenase.** (A) Electron transfer processes between a cellular ferredoxin (Fd) or flavodoxin (Fld) and the oxidized Fe protein (FeP_ox_) to result in reduced Fe protein (FeP_red_). Further ATP:ADP exchange is required for an FeP_red_ that can complex to the catalytic protein (MoFeP) and result in electron transfer (ET) to advance each E_n_ redox state. (B) Catalytic cycle of N_2_ase and current structural models for hydride and hydrosulfide formation through the E_n_ states.^6,79,80^

### 2.2 Alternative Reactions Catalyzed by Nitrogenase

In addition to N_2_ fixation, there are several reactions that N_2_ase catalyzes *in vitro* and *in vivo* with biological relevance. The FeFe N_2_ase exhibits the highest activity for the eight-electron reduction of CO_2_ to CH_4_,^81–84^ followed by the VFe N_2_ase and MoFe N_2_ase isoforms.^85–88^ These methane formation activities are sufficient to sustain growth of methylotrophic bacteria (*Methylomonas* sp.) in coculture and may contribute to carbon cycling in anoxic environments.^81^ Alternatively, the FeFe N_2_ase, and to a lesser extent MoFe and VFe N_2_ases, can also perform the two-electron reduction of CO_2_ to CO or formate (HCOO^–^), which can serve as carbon and energy sources for many autotrophic and heterotrophic bacteria. FeFe N_2_ase activities for CO_2_ reduction to CO and formate are approximately 10-fold higher than for reduction of CO_2_ to CH_4_.^85,89–92^ Lastly, although the biological relevance is less clear, N_2_ases can reduce CO to methane and reduce and couple CO and CO_2_ molecules together to form hydrocarbons like ethylene, ethane, butylene, and longer alkanes up to 10-carbons in length.^8,89,92–95^ Multiple species of bacteria and thermophilic methylotrophic archaea from oceanic vents recently have been identified to anaerobically oxidize short chain alkanes for growth, including ethane, propane, and butane.^96–99^ However, whether bacteria and archaea utilize nitrogenase-originating hydrocarbons in the environment as compared to recognized petroleum-originating hydrocarbons is unknown. Beyond biologically relevant reactions, N_2_ase can also catalyze numerous reduction reactions involving double- and triple-bond containing molecules with C, N, S, and O atoms. Such processes are comprehensively reviewed elsewhere.^6,9^

### 2.3 Biological Function and Nomenclature of NFL Systems

NFL systems, often referred to as “nitrogenase-like” systems, are also observed across all domains of life with structural and sequence homology to N_2_ase for catalyzing specific reactions involved in carbon, energy, sulfur, and nitrogen metabolism (Figure 3). Methanogenic and methylotrophic archaea possess CfbCD NFL systems for the synthesis of F430. Cofactor F430 functions in the interconversion of methyl-CoM and CoB to CoM-CoB heterodisulfide and CH_4_ by methyl-CoM reductase. Photosynthetic bacteria, algae, and some plants possess ChlLNB, BchLNB, and BchXYZ NFL systems for the synthesis of bacteriochlorophyll and chlorophyll as light harvesting pigments for photosynthesis. Further, some anaerobic bacteria possess MarBHDK and IsrBHDK NFL systems that reductively cleave C–S bonds present in organic sulfur compounds for bacterial sulfur acquisitions. Further still, other anaerobic bacteria possess enigmatic NfaBHDK NFL systems that appear to fix N_2_, and yet, these NFL proteins lack quintessential residues known in N_2_ase to be required for N_2_ fixation. In this section, the essential functions and reactions of NFL systems are introduced to lay the foundation for detailed analysis of the current state of understanding for each system in sections 4–9. In addition, as detailed in section 2.4, years of phylogenetic studies of N_2_ase and NFL systems have been conducted as these systems have been uncovered in succession. Phylogenetic categorization has resulted in each N_2_ase and NFL phylogenetic groups being designated with a number, and where necessary a subgroup or “clade” letter, to demarcate their position within the N_2_ase superfamily of sequences.

**Figure 3:**
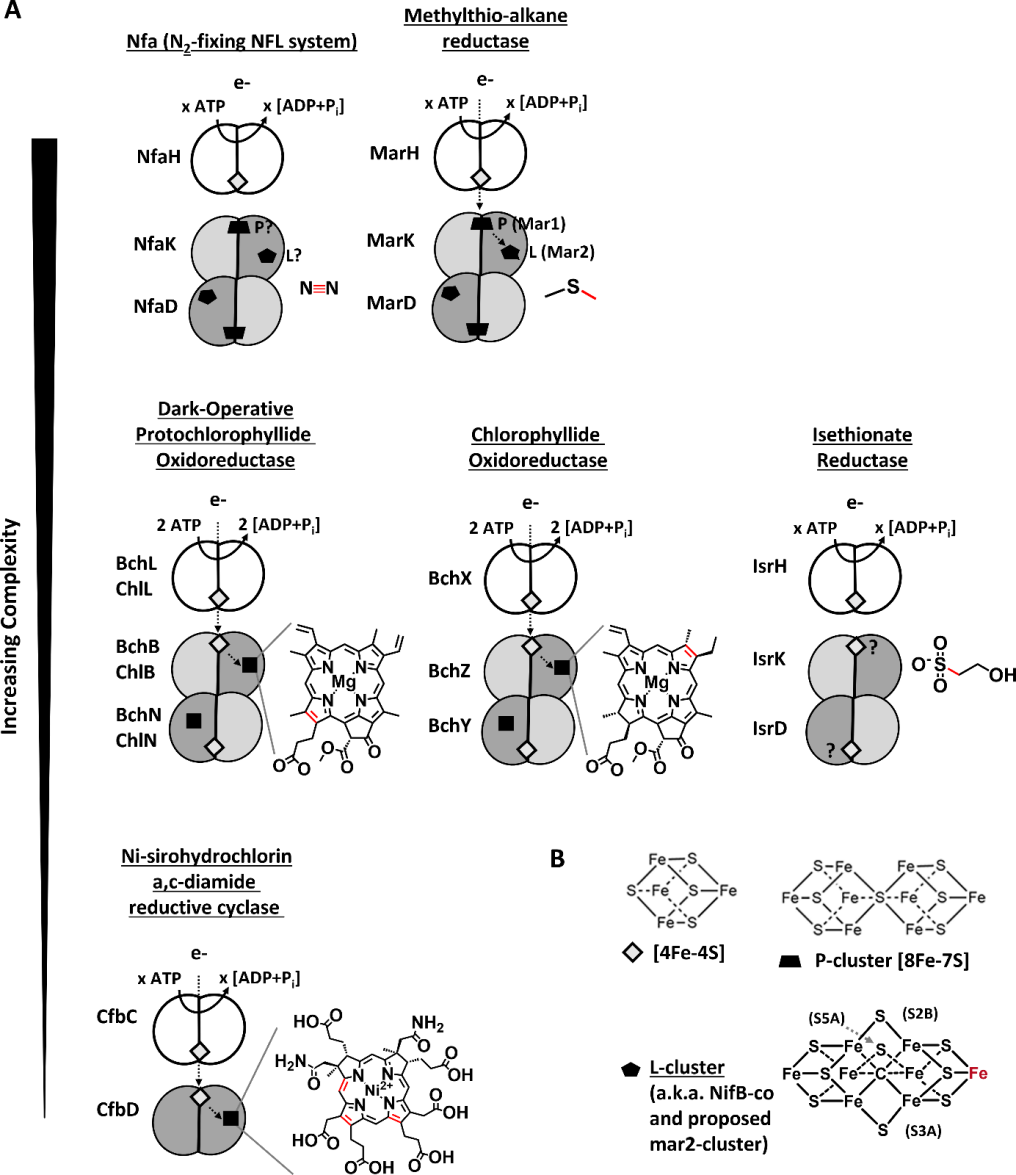
**Enzyme systems that are homologous to nitrogenase: the nitrogen fixation-like proteins.** (A) Nitrogen fixation-like systems of known function ranked by subunit complexity. (B) Metallocofactor composition of nitrogen fixation-like systems. Dashed arrows indicate routes of known electron transfer for substrate reduction. Black squares indicate observed location of indicated tetrapyrrole substrate binding. Red bonds indicate location of reduction. For DPOR, the [4Fe-4S] cluster of BchNB or ChlNB is termed the NB-cluster. In methylthio-alkane reductase, a P-cluster (mar1-cluster) and L-cluster (mar2-cluster) are implicated by cryo-EM, spectroscopic, and genetic evidence.^100,101^ However, the presence of a central carbide is unverified. The proposed Nfa P-cluster and L-cluster is solely based on sequence identity to methylthio-alkane reductase.^102,103^ The isethionate reductase [4Fe-4S] cluster is proposed based on sequence homology to the DPOR and COR systems.^104^

#### 2.3.1 Nitrogen Fixation Alternative Enzyme (Nfa)

The nitrogen fixation alternative (Nfa) systems are Group IV-A NFL systems (no E.C. number yet assigned) composed of NfaBHDK. Nfa systems have been implicated genetically and biochemically in the reduction of N_2_ for diazotrophic growth to low culture densities of certain *Endomicrobium* species^105^ and *Heliophilum fasciatum*,^106,107^ as fully discussed in section 4. While no Nfa system has yet to be isolated, the NfaBHDK protein sequences are homologous to N_2_ase by virtue of a component 2 Fe protein sequence, NfaH, and presumed component 1 sequences NfaDK (Figure 3). NfaB is anticipated to be involved in metallocofactor assembly analogous to N_2_ase NifB function in FeMo-co synthesis (Figure 1), but the Nfa system metallocofactors are currently unknown. Based on sequence identity, Nfa is phylogenetically located within the NFL sequences instead of with all the known extant MoFe, VFe, and FeFe N_2_ases. Further NfaD is devoid of key N_2_ase residues known to be required for N_2_ fixation, raising interesting questions as to how N_2_ may be reduced by these NFL systems (Figure 11). The overall reaction scheme of the Nfa systems is unknown, but at minimum it is anticipated that N_2_ is reduced to 2 equivalents of ammonia, and Nfa may catalyze alternative albeit unknown biologically relevant reactions in analogy to those observed for N_2_ase (section 2.2).

#### 2.3.2 Dark-Operative Protochlorophyllide Oxidoreductase (DPOR)

DPOR is a Group V NFL system (E.C. 1.3.7.7) involved in biosynthesis of photosynthetic pigments for energy metabolism, as fully discussed in section 5. DPOR systems are designated as BchLNB or ChlLNB, depending on their involvement in bacteriochlorophyll or chlorophyll synthesis, respectively. They are two-component systems comprising a BchL or ChlL homodimeric Fe protein and a BchNB or ChlNB heteromeric catalytic complex (Figure 3). No metallocofactor synthesis protein homologous to NifB is associated with DPOR systems, and they are observed to function using only [4Fe-4S] clusters. DPOR thus catalyzes the following C=C double bound reduction reactions within a tetrapyrrole-based macrocycle^108–110^ (Figure 4):

$$\mathrm{8‐vinyl‐protochlorophyllide}\;a+2e^{-}+2H^{+}+4\mathrm{MgATP}\to \mathrm{8‐vinyl‐chlorophyllide}\;a+4\left[\mathrm{MgADP}+P_{i}\right]$$ 5

$$\mathrm{protochlorophyllide}\;a+{2e}^{-}+2H^{+}+4\mathrm{MgATP}\to \mathrm{chlorophyllide}\;a+4\left[\mathrm{MgADP}+P_{i}\right]$$ 6

$$\mathrm{protochlorophyllide}\;b+2e^{-}+2H^{+}+4\mathrm{MgATP}\to \mathrm{chlorophyllide}\;b+4\left[\mathrm{MgADP}+P_{i}\right]$$ 7

where two ATP are hydrolyzed per electron transferred from the Fe protein (component 2) to the catalytic complex (component 1).^110^

**Figure 4:**
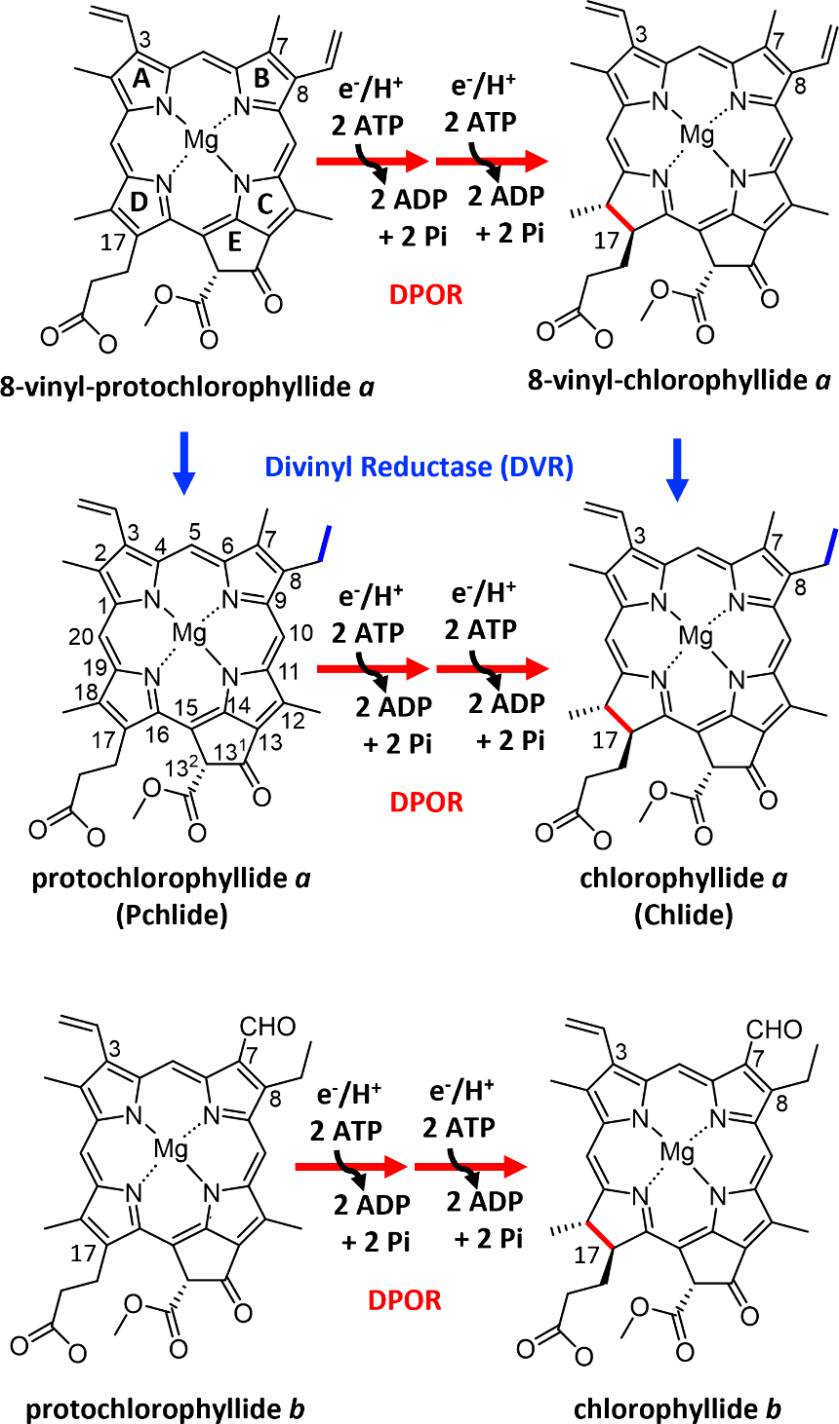
Specific reactions catalyzed by DPOR in chlorophyll and bacteriochlorophyll synthesis.

Notably, chlorophyllide *a* (Chlide), which is made by DPOR, is a common precursor to both chlorophyll *a* and bacteriochlorophylls. As such, DPOR systems that synthesize Chlide as an intermediate in plant, algae, and gymnosperm chlorophyll *a* synthesis are designated as ChlLNB (for chlorophyll), whereas DPOR systems that synthesize Chlide and chlorophyllide *b* as an intermediate in bacteriochlorophyll synthesis are designated as BchLNB (for bacteriochlorophyll). DPOR is distinct from the plant light-dependent protochlorophyllide oxidoreductase (LPOR; E.C. 1.3.1.33), which catalyzes the light- and NADPH-dependent reactions:^111^

$$\mathrm{8‐vinyl‐protochlorophyllide}\;a+\mathrm{NADPH}+H^{+}+h\nu \to \mathrm{8‐vinyl‐chlorophyllide}a+{\mathrm{NADP}}^{+}$$ 8

$$\mathrm{protochlorophyllide}\;a+\mathrm{NADPH}+H^{+}+h\nu \to \mathrm{chlorophyllide}\;a+{\mathrm{NADP}}^{+}$$ 9

DPOR and LPOR ultimately result in the same chlorophyllide *a* (Chlide) or 8-vinyl-Chlide intermediate of chlorophyll/bacteriochlorophyll biosynthesis. However, LPOR is not a sequence homologue of DPOR. No significant amino acid similarity is found between the two enzymes except for a 14 amino acid sequence called the TFT motif (TFTAEGF-X-LSRLLLD) found in both the DPOR ChlL Fe protein and LPOR.^112^ The TFT motif is otherwise absent in short-chain dehydrogenase family proteins to which LPOR belongs, and is absent from the NifH Fe protein of N_2_ase, indicating convergent evolution for LPOR and DPOR.^112,113^

#### 2.3.3 Chlorophyllide Oxidoreductase (COR)

COR is a Group V NFL system (E.C. 1.3.7.15) that is a close relative of DPOR and is specifically involved in the later steps of bacteriochlorophyll synthesis in bacteria as fully detailed in section 5. COR systems are designated as BchXYZ and exhibit the same architecture and metallocofactor identities as DPOR systems (Figure 3). The “a” isoform, a-COR, catalyzes the following C=C double bond reduction reactions within tetrapyrrole-based macrocycles for bacteriochlorophyll *a* synthesis^114^ (Figure 5A):

$$\mathrm{8‐vinyl‐chlorophyllide}\;a+2e^{-}+2H^{+}+4\mathrm{MgATP}\to \mathrm{chlorophyllide}\;a+4\left[\mathrm{MgADP}+P_{i}\right]$$ 10

$$\mathrm{chlorophyllide}\;a+2e^{-}+2H^{+}+4\mathrm{MgATP}\to \mathrm{3‐vinyl‐bacteriochlorophyllide}\;a+4\left[\mathrm{MgADP}+P_{i}\right]$$ 11

$$\mathrm{3‐acetyl‐chlorophyllide}\;a+2e^{-}+2H^{+}+4\mathrm{MgATP}\to \mathrm{bacteriochlorophyllide}\;a+4\left[\mathrm{MgADP}+P_{i}\right]$$ 12

The “b” isoform, b-COR, catalyzes the following reaction for bacteriochlorophyll *b* synthesis^114–116^ (Figure 5B):

$$\mathrm{8‐vinyl‐chlorophyllide}\;a+2e^{-}+2H^{+}+4\mathrm{MgATP}\to \mathrm{3‐vinyl‐bacteriochlorophyllide}\;b+4\left[\mathrm{MgADP}+P_{i}\right]$$ 13

where 3-vinyl-bacteriochlorophyllide *b* is also known as biacteriochlorophyllide *g*.

**Figure 5:**
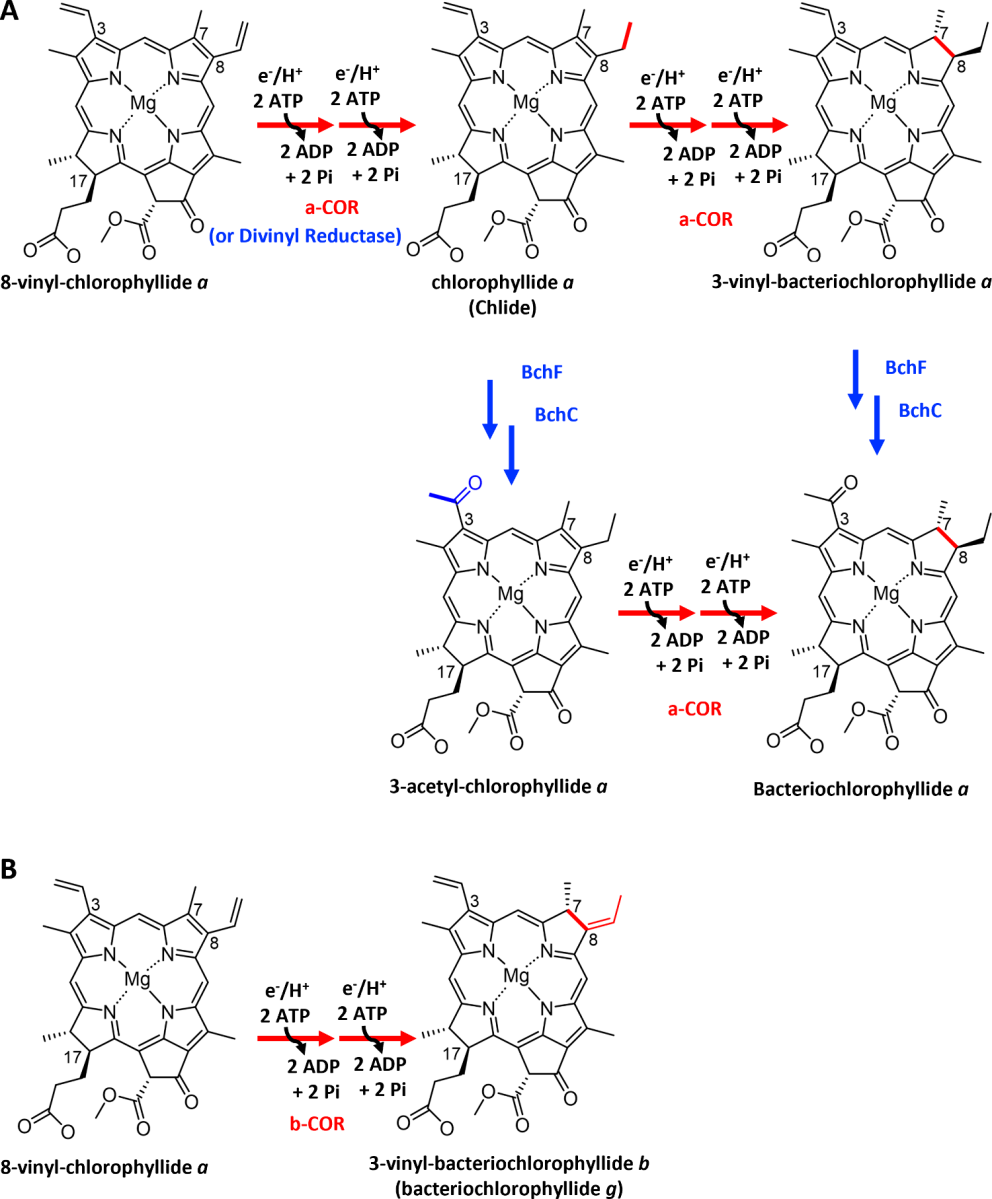
**Specific reactions catalyzed by COR in bacteriochlorophyll synthesis.** Reactions catalyzed by the (A) a-COR isoform and (B) b-COR isoform.^114–116^

#### 2.3.4 Ni^2+^-Sirohydrochlorin *a*,*c*-Diamide Reductive Cyclase (CfbCD)

CfbCD is a Group IV-B NFL system (E.C. 6.3.3.7) that is involved in synthesis of cofactor F430. F430 is present in the methyl-CoM reductase enzyme that catalyzes the final step of methanogenesis and first step of anaerobic oxidation of methane (AOM), as fully detailed in section 6. CfbCD to date is the simplest NFL system comprising a homodimeric Fe protein (component 2) and homomeric catalytic core (component 1) (Figure 3). CfbCD catalyzes a sequence of multiple C=C double bond reduction reactions and C–N bond formation for cyclization on the same tetrapyrrole-based macrocycle substrate to yield the overall reaction (Figure 6):

$${\mathrm{Ni}}^{2+}\mathrm{‐sirohydrochlorin}\;a,c\mathrm{‐diamide}+6e^{-}+7H^{+}+x\mathrm{MgATP}\to 15,17^{3}{\mathrm{‐seco‐F430‐17}}^{3}\mathrm{‐acid}+x\left[\mathrm{MgADP}+P_{i}\right]$$ 14

where the stoichiometry of ATPs hydrolyzed per electron transferred is unknown.

**Figure 6:**
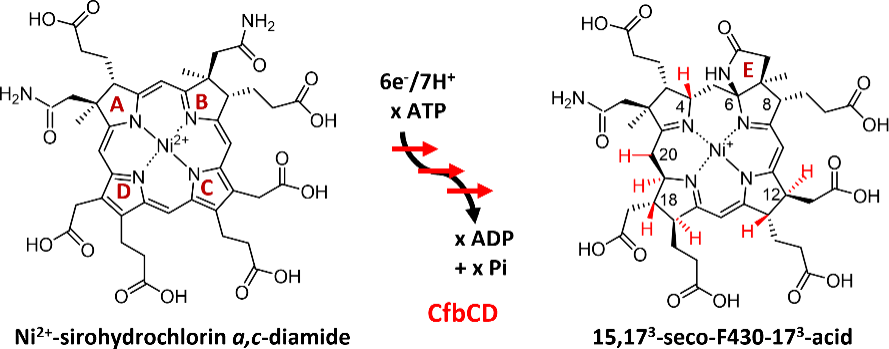
**Specific reaction catalyzed by CfbCD in F430 cofactor biosynthesis.** CfbCD is a Ni^2+^-sirohydrochlorin *a*,*c*-diamide reductive cyclase.^117^ The multiple arrows indicate the likely, albeit unknown, sequential reduction of the substrate by one-electron at a time.

#### 2.3.5 Methylthio-alkane Reductase (MAR)

MAR is a Group IV-C NFL system (no E.C. number yet assigned) that is involved in the reductive cleavage of C–S bonds within volatile organic sulfur compounds. Biologically, the reduction of the methylthio-alkanes produces methanethiol, which is a direct precursor for the synthesis of methionine in many bacteria, as fully discussed in section 8.^102,118–120^ MarBHDK is a two-component system homologous to N_2_ase comprising a MarH homodimeric Fe protein and MarDK heteromeric catalytic complex (Figure 3). Remarkably, while there is much to be understood about the precise metallocofactor identities of MAR, several lines of evidence indicate the presence of a P-cluster and an L-cluster or L-like cluster previously thought only to exist in N_2_ase. In particular, MarB, a homologue of NifB, is required for cluster assembly. Genetic and spectroscopic evidence indicate that MarB makes the same, or similar, L-cluster (a.k.a. NifB-co) that is synthesized by NifB.^100,101^ Currently verified substrates and products catalyzed by MAR are^100–102^ (Figure 7A):

$$\mathrm{dimethyl}\;\mathrm{sulfide}+2e^{-}+2H^{+}+x\mathrm{MgATP}\to \mathrm{methane}+\mathrm{methanethiol}+x\left[\mathrm{MgADP}+P_{i}\right]$$ 15

$$\mathrm{ethyl}\;\mathrm{methyl}\;\mathrm{sulfide}+2e^{-}+2H^{+}+x\mathrm{MgATP}\to \mathrm{ethane}+\mathrm{methanethiol}+x\left[\mathrm{MgADP}+P_{i}\right]$$ 16

$$\mathrm{2‐methylthioethanol}+2e^{-}+2H^{+}+x\mathrm{MgATP}\to \mathrm{ethylene}+\mathrm{methanethiol}+H_{2}O+x\left[\mathrm{MgADP}+P_{i}\right]$$ 17

$$\mathrm{acetylene}+2e^{-}+2H^{+}+x\mathrm{MgATP}\to \mathrm{ethylene}+x\left[\mathrm{MgADP}+P_{i}\right]$$ 18

$$\mathrm{acetylene}+4e^{-}+4H^{+}+x\mathrm{MgATP}\to \mathrm{ethane}+x\left[\mathrm{MgADP}+P_{i}\right]$$ 19

$$2e^{-}+2H^{+}+x\mathrm{MgATP}\to H_{2}+x\left[\mathrm{MgADP}+P_{i}\right]$$ 20

Notably, while hydrogen evolution is also observed during reduction of organic substrates (reactions 14–18), it is not known whether hydrogen production is an obligate part of the MAR catalytic mechanism, as it is for N_2_ase.^101^ Also, the stoichiometry of ATP hydrolyzed per electron transferred is unknown.

**Figure 7:**
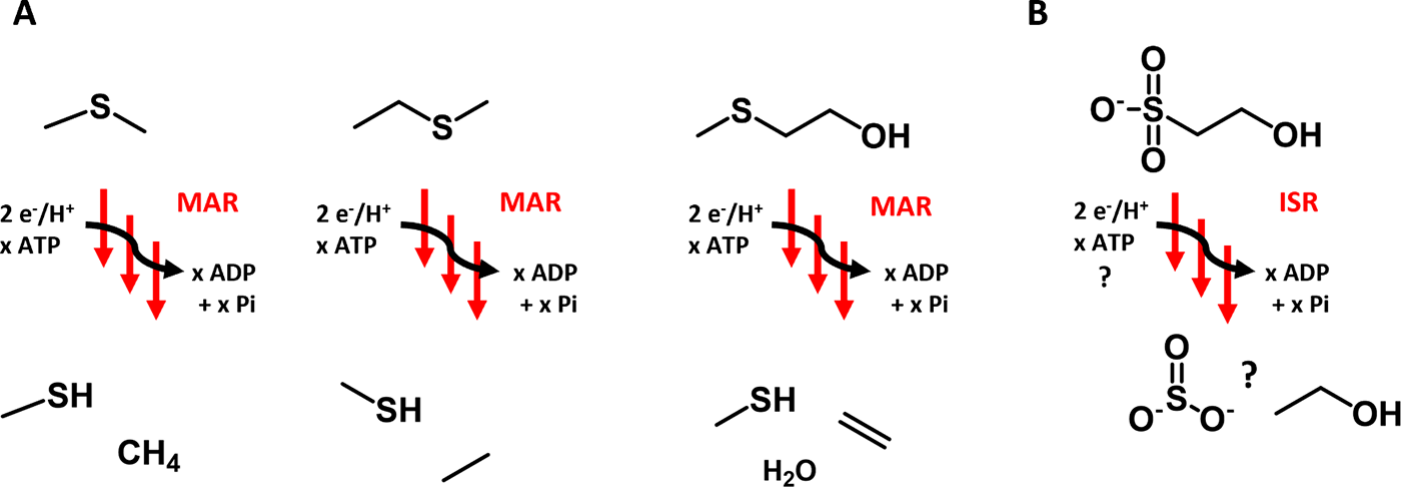
**Specific reactions with organic sulfur compounds catalyzed by NFL systems.** (A) Methylthio-alkane reductase (MAR).^100–102^ (B) Isethionate reductase (ISR) with proposed reactions.^104^ The multiple arrows indicate the likely, albeit unknown, sequential reduction of substrates by one electron at a time. Methanethiol is further utilized for methionine biogenesis via *O-*acetyl-l-homoserine sulfhydrylase (E.C. 4.2.99.10).^120^

#### 2.3.6 Isethionate Reductase (ISR)

ISR is a Group IV-F NFL system (no E.C. number yet assigned) that is involved in the cleavage of isethionate for bacterial sulfur acquisition. IsrBHDK is a two-component system homologous to N_2_ase comprising an IsrH homodimeric Fe protein and IsrDK heteromeric catalytic complex. The identities of the ISR metallocofactors are entirely unknown. However, based on sequence identity with DPOR and COR, ISR systems are predicted to function using [4Fe-4S] clusters (Figure 3). Intriguingly, the associated NifB homologue, IsrB, only possesses a radical SAM domain, raising questions as to its role in metallocofactor assembly and/or catalysis, as fully detailed in section 9.^104^ While the reaction has not been fully verified regarding the ethanol and sulfite products, the proposed ISR reaction is as follows (Figure 7B):

$$\mathrm{isethionate}+2e^{-}+2H^{+}+x\mathrm{MgATP}\to \mathrm{ethanol}+{{\mathrm{SO}}_{3}}^{2-}+x\left[\mathrm{MgADP}+P_{i}\right]$$ 21

In summary, in analogy to N_2_ase, all of these NFL systems with distinct functions are verified or at least implicated to be two-component systems composed of an Fe protein homologue (component 2) for ATP-dependent, intermolecular electron transfer via a single [4Fe-4S] cluster, and a catalytic core (component 1) that performs substrate reduction. Furthermore, save for the Ni^2+^-sirohydrochlorin *a*,*c*-diamide reductive cyclase, whose catalytic component 1 is homomeric, all of the other NFL systems appear to form heterotetrametric complexes analogous to N_2_ase (Figure 3). What distinguishes these systems, in part, is the observed or inferred metallocofactor based on sequence alignment as fully detailed in sections 4–9. While the N_2_ase catalytic component uses a P-cluster and catalytic cluster (e.g., FeMo-co), only a single [4Fe-4S] cluster is present in each of the dimeric interfaces of DPOR, COR, Cfb, and presumably ISR. Paramount to the NFL field, recent work by multiple groups has revealed that certain systems including MAR for C–S bond cleavage, and likely Naf for N_2_ fixation, utilize complex P-like and L-like clusters for substrate reduction. The presence of P- and L-clusters in NFL systems reveals that N_2_ase metallocofactors have been employed by biology beyond N_2_ fixation.^100,101,105,106^

### 2.4 Phylogeny and Sequence Features of Nitrogenase and NFL Systems

Sequence and phylogenetic analysis of N_2_ase and NFL proteins, both early on and recently, have identified distinct groups of sequences.^101,102,121–130^ Currently there are 5 identified major groups of functional N_2_ases based on sequence similarity with NifD (Figures 8 and 9). All of these N_2_ase NifD homologue sequences possess 3 conserved cysteine residues for coordinating one-half of the P-cluster at the NifDK interface. Likewise, all of the associated N_2_ase NifK homologue sequences possess 3 conserved cysteine residues for coordinating the other half of the P-cluster (Figures 9 and 10). Also, the NifD homologue sequences possess a conserved cysteine and histidine residue pair for FeMo-co coordination within NifD (Figures 9 and 11). Group I contains MoFe N_2_ase NifD sequences primarily found in facultative proteobacteria and cyanobacteria, including *A. vinelandii*. Similarly, Group II contains MoFe N_2_ase NifD sequences primarily found in anaerobic bacteria (Bacilliota, Firmicutes) and some archaea. Group III contains both VFe N_2_ase VnfD sequences and FeFe N_2_ase AnfD sequences found across bacteria and archaea, as well as some MoFe N_2_ase NifD sequences from archaea. In addition, earlier work identified N_2_ase sequences within Group III from *Candidatus Desulforudis audaxviator* and *Thermodesulfatator indicus* with a selenocysteine (U) that replaces the conserved Cys62 P-cluster coordination residue (*A. vinelandii* numbering, Figure 10).^128^ Among the Group III NifD sequences, multiple organisms are observed to only possess NifE (no NifN; Figure 1) for possible FeMo-co maturation. Further, a subgroup of Group III NifD sequences are found in thermophilic archaea and bacteria like *Roseiflexus* sp. that lack any observable NifEN sequences. Instead, these Group III N_2_ases are thought to assemble their metallocofactor directly on the NifDK scaffold (see section 2.5 below). However, demonstrable diazotrophic growth of a microbe lacking NifEN still remains elusive.^23,131,132^ It is noteworthy that the NifD subgroup of sequences of Group III were initially called Group IV by Howard et al.,^128^ and not to be confused with the Group IV terminology now used to designate the NFL sequences. The final two distinct NifD sequence groups are the “methanosarcina-like” (MSL) and Group VII, which were recently identified in the genomes and metagenomes of archaea, including ANME, in deep sea cold-seep sediments.^122^

**Figure 8:**
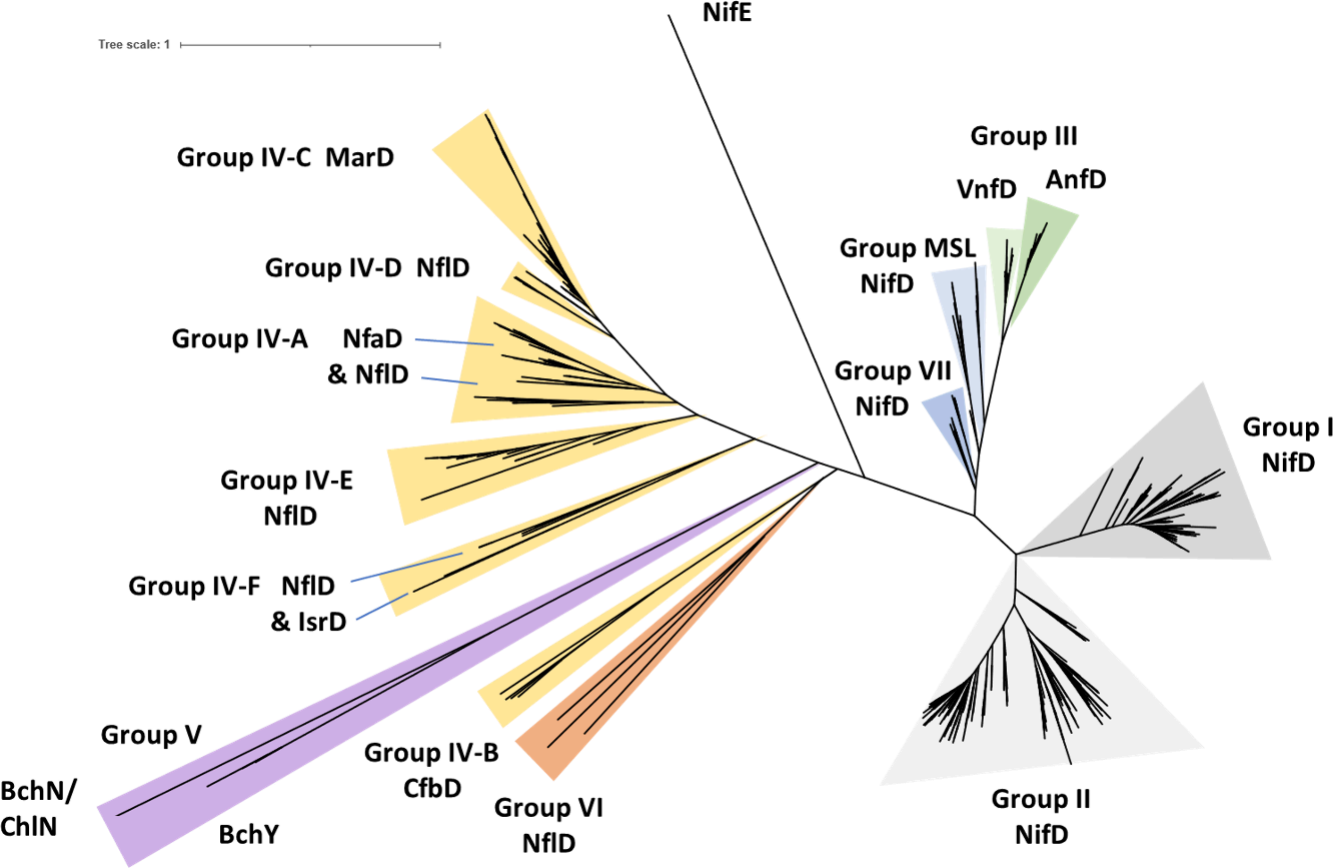
**Phylogenetic tree of N**_**2**_ **ase and NFL sequences.** The tree was reconstructed from sequences provided in references,^102,122,130^ rooted with NifE as the outgroup, and bootstraps hidden for clarity. Groups I and II are the MoFe N_2_ases of facultative bacteria/cyanobacteria and anaerobic clostridia/archaea, respectively. Group III contains the VFe and FeFe N_2_ases, along with some bacteria/archaea MoFe sequences.^130^ MSL, the “methanosarcina-like” NifD clade and Group VII N_2_ases are recently identified archaeal sequences from oceanic cold-seeps.^122^ Groups IV–VI are NFL sequences. Group IV sequences of known function include Group IV-A NfaD catalyzing N_2_ fixation,^105^ Group IV-B CfbD catalyzing F430 cofactor synthesis,^117^ Group IV-C MarD catalyzing volatile organic sulfur compound cleavage,^102^ and Group IV-F IsrD catalyzing isethionate cleavage.^104^ Group V is DPOR (BchN/ChlN) and COR (BchY) for bacteriochlorophyll biosynthesis, and Group VI NflD is proposed to be involved in cofactor synthesis.^121^ All sequences that are not Nif, Vnf, or Anf N_2_ases are by default nitrogen fixation-like.

**Figure 9:**
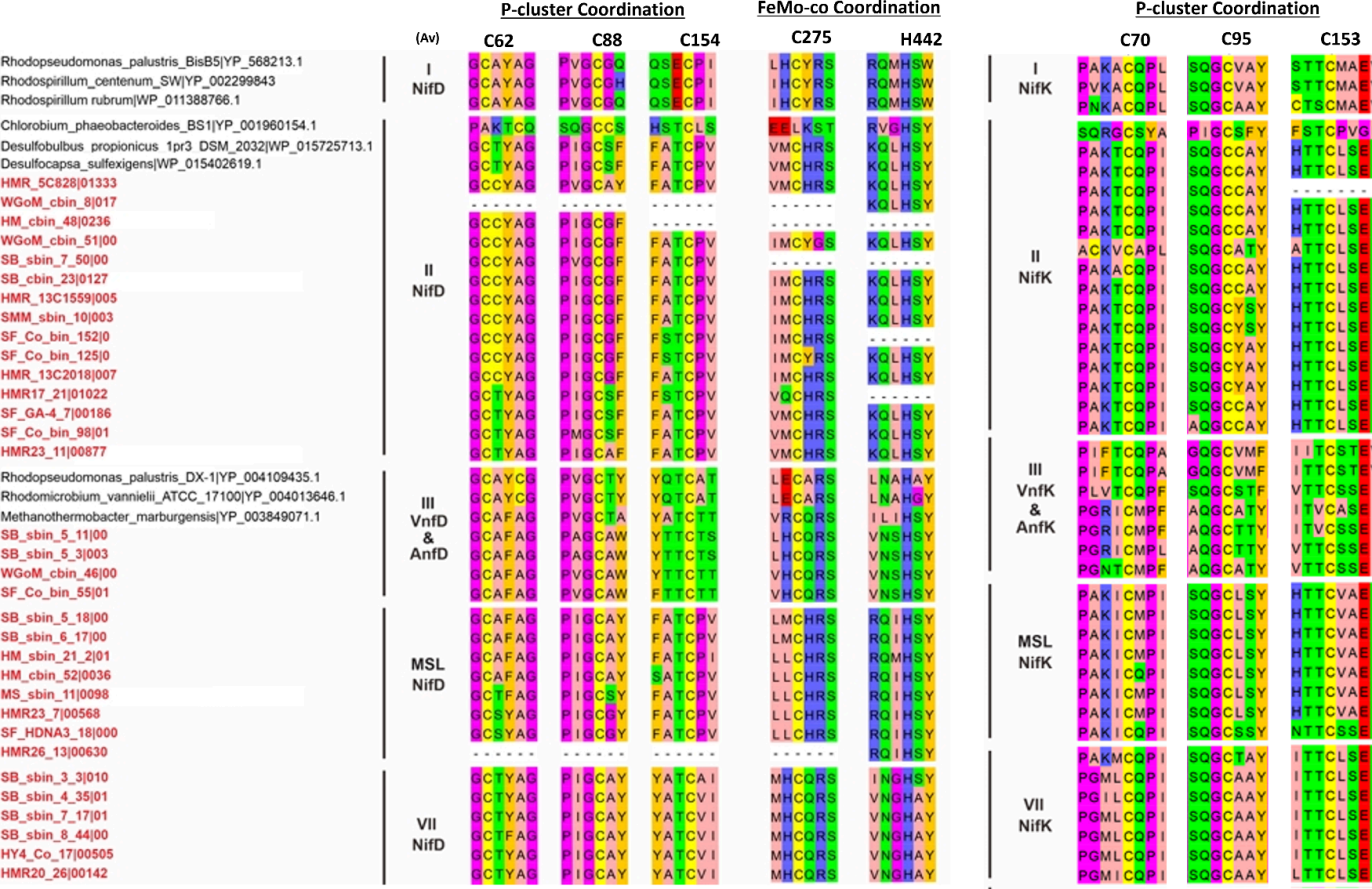
**Sequence conservation between N**_**2**_ **ase proteins for P-cluster and FeMo-co coordination.** Shown in red are bacterial (Group II and III) and archaeal (Group III, “methanosarcina like” (MSL), and VII) sequences identified in deep-sea environments. Shown in black are select reference terrestrial microbial N_2_ases. The sequences displayed are the set used by Dong et al.,^122^ demonstrating organisms possessing N_2_ase are globally distributed. Adapted from ref 122 to omit NFL sequences. CC BY 4.0.

**Figure 10:**
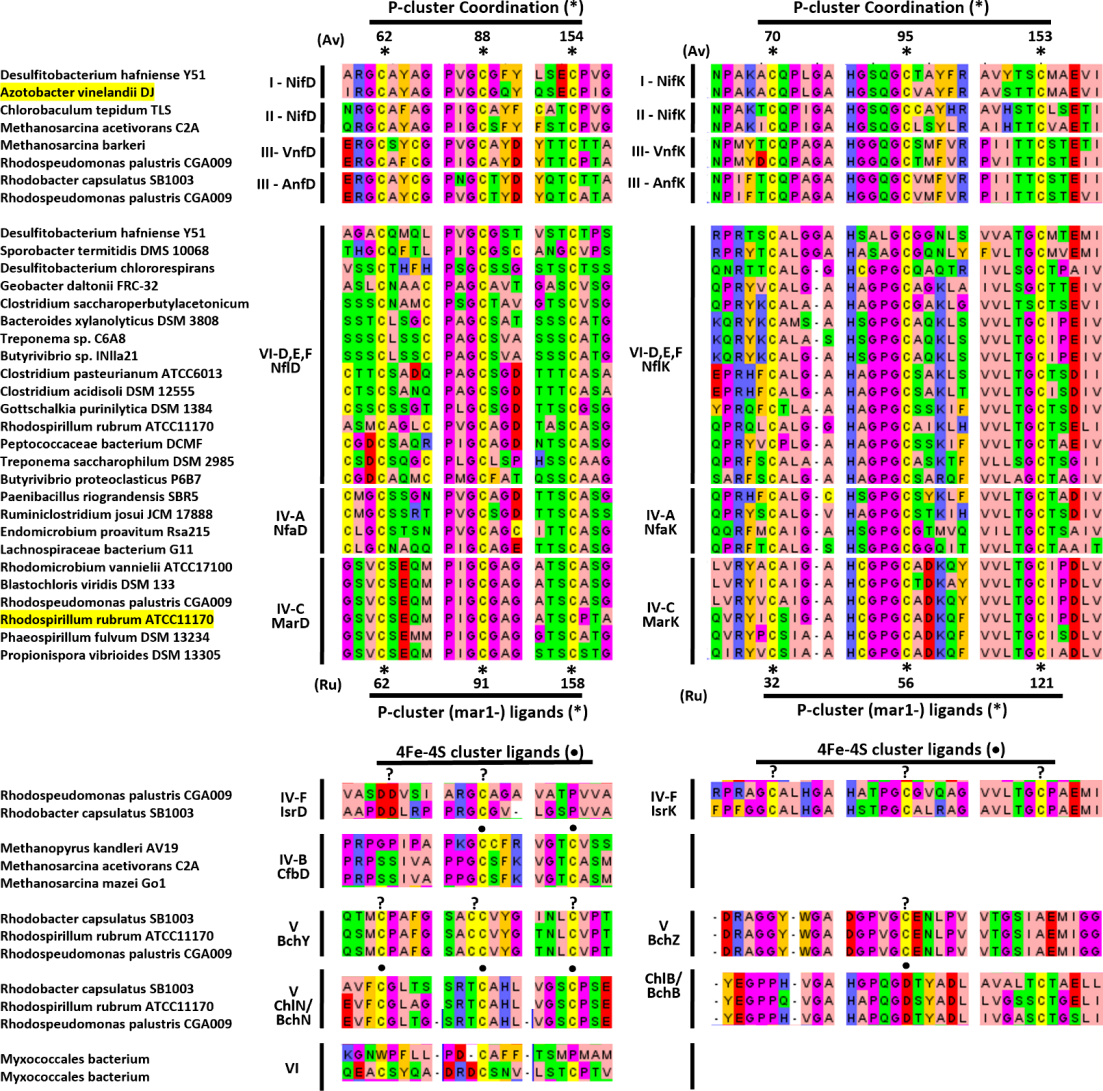
**Sequence conservation between N**_**2**_ **ase and NFL proteins for N**_**2**_ **ase P-cluster coordination.** Symbols and numbering correspond to known metallocofactor coordination of N_2_ase and MAR P-clusters from *A. vinelandii* and *R. rubrum*, respectively. For CfbD and DPOR ChlNB/BchNB, specific [4Fe-4S] metallocofactor coordination is known (•). In contrast, [4Fe-4S] metallocofactor coordination is only predicted for ISR and in implicated for COR BchYZ by mutagenesis studies (**?**). Sequences are from ref 102. Accession numbers for the sequences are provided in Figure 11. The *A. vinelandii* NifD and *R. rubrum* MarD sequences are highlighted for reference.

**Figure 11:**
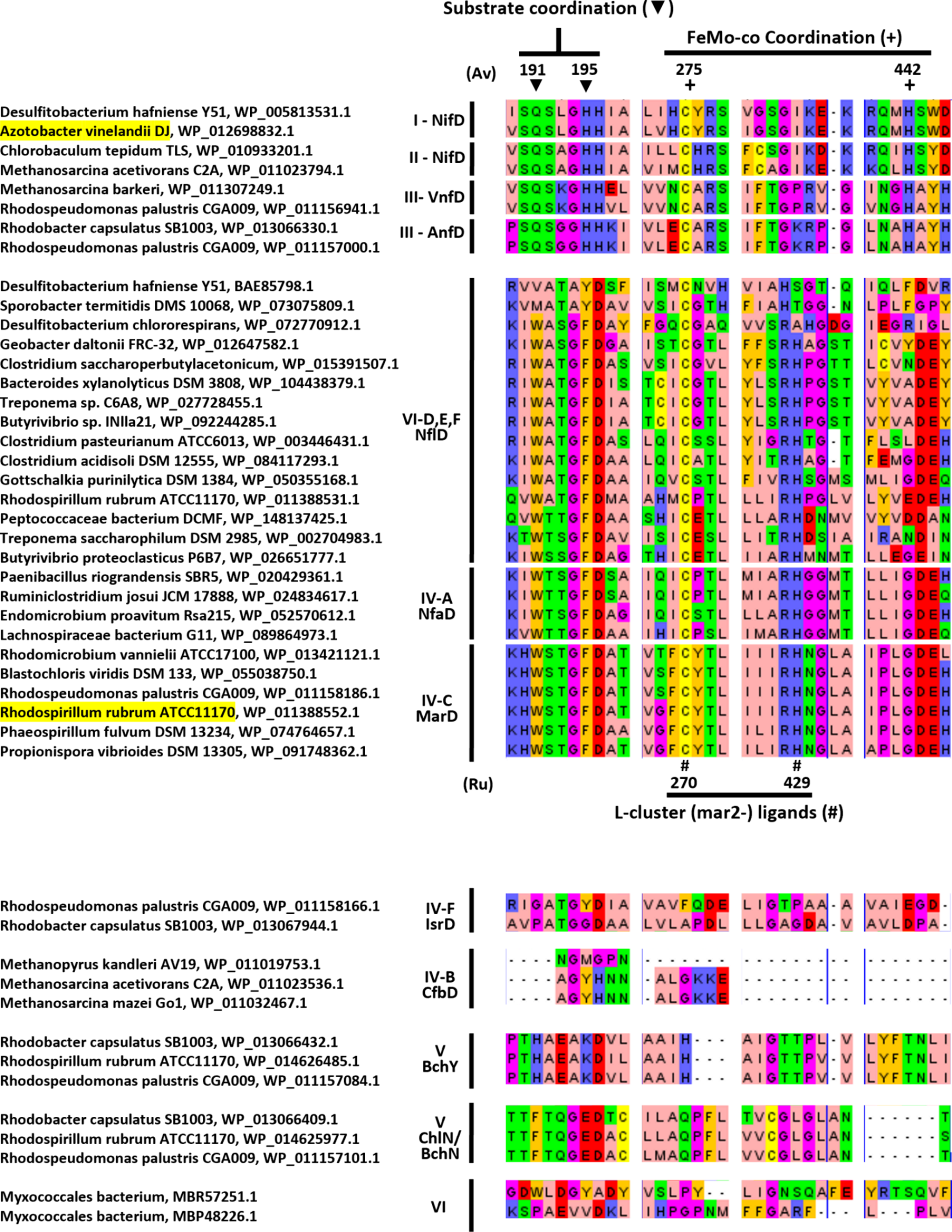
**Sequence conservation between N**_**2**_ **ase and NFL proteins for N**_**2**_ **ase FeMo-co coordination.** Symbols and numbering correspond to known metallocofactor coordination of N_2_ase FeMo-co and MAR L-cluster (mar2-cluster) from *A. vinelandii* and *R. rubrum*, respectively. For CfbD, DPOR ChlN/BchN, COR BchY, IsrD, and Group VI NFL there are no known conserved metallocofactor coordinating residues in this region. Sequences are from ref 102, and NCBI accession numbers are given for each sequence. The *A. vinelandii* NifD and *R. rubrum* MarD sequences are highlighted for reference.

All known NifD sequence homologues beyond the functional N_2_ase sequences of Groups I, II, III, MSL, and VII are termed NFL sequences. NFL sequences do not cluster with the true N_2_ase sequences, but instead form separate groups and clades that in general reflect their respective functions.^101,121–123^ Given the temporal distances over which the NFL sequence groups have been discovered due to progressive advancements in the sequencing of microbes from previously uncharacterized environments, there is not a straightforward and codified naming scheme of the NFL groups. The initial NFL systems discovered were the (bacterio)­chlorophyll oxidoreductases, DPOR and COR, which later were specifically designated as Group V. All other NFL sequence groups of unknown function were lumped as Group IV with alphabetical subgroups (Figure 8). Such subgroup labels have been added over time as functions were ascribed to existing clades, or new clades were observable as new sequences were included from advancements in microbial genome sequencing. Specifically, in 2016 the Group IV-A NFL designation was given to NfaD (nitrogen fixation alternative) homologues of *Endomicrobium proavitum* involved in N_2_ fixation.^105,121^ Subsequently in 2016, the discovered CfbD homologues involved in F430 cofactor synthesis were designated as Group IV-B.^117,133^ With the identification of Methylthio-alkane Reductase in 2020, the homologous MarD sequences were designated as Group IV-C, and the remaining Group IV clades of unknown function were labeled D-F at that time to minimize future confusion and discrepancies.^102^ In 2024, Group IV-F ISR sequences were identified to function as isethionate reductase.^104^ Lastly, Group VI NflD sequences proposed to be involved in tetrapyrrole-based cofactor synthesis were added to the NFL family in 2020 after their identification in diverse Elusimicrobia species. Designation of Group VI as an entirely new group was required due to its high sequence divergence from the already established Group IV NFL sequences. Further, the implicated role of Group VI sequences in tetrapyrrole-based cofactor synthesis analogous to established Group V DPOR and COR sequences also justified their specific Group VI designation (Figure 8).^102,121^

A key feature of the NFL sequences is their partial or complete divergence from N_2_ase sequences at amino acid residue positions involved in metallocofactor and N_2_ substrate coordination. While most Group IV NFL sequences exhibit the conserved cysteine residues present in N_2_ase for P-cluster coordination, the conserved histidine residue used by N_2_ase for catalytic FeMo-co binding is absent (Figure 10). Further, Group IV-F ISR, Group V DPOR and COR, and Group VI of proposed tetrapyrrole-based synthesis all diverge from N_2_ase with respect to the conserved N_2_ase cysteine residues for P-cluster coordination, and conserved cysteine and histidine residues for FeMo-co coordination. These NFL sequences, as further detailed in sections 4–9, are thus either observed or predicted to coordinate one or more specific metallocofactors for electron transfer and catalysis that differ from N_2_ase. Specifically, Methylthio-alkane Reductase (MAR; Group IV-C) is observed, and most but not all sequences of Groups IV-A,C,D,E,F are predicted to coordinate a P-cluster or P-like cluster (Figure 10). These same sequences are also observed, or otherwise predicted, to bind an L-cluster or L-like cluster.^100,101^ In contrast, residues of the dimeric Group IV-A CfbCD system for F430 cofactor synthesis symmetrically coordinate only a [4Fe-4S] cluster. In DPOR BchNB/ChlNB the [4Fe-4S] NB-cluster is asymmetrically coordinated by 3 cysteine residues from BchN/ChlN and an aspartate from BchB/ChlB (Figure 10). The three [4Fe-4S] coordinating cysteine residues of BchN/ChlN correspond to the three NifD P-cluster binding residues in sequence alignments, and the unique [4Fe-4S] coordinating aspartate is in the same sequence position as one of the NifK cysteine residues for P-cluster coordination.^134^ Depending on the input sequences and methods used in the alignment analysis, the DPOR ChlN/BchN and COR BchY cysteine residue corresponding to NifD Cys62 can also align with the nonconserved VnfD/AnfD Cys65 (Ala65 in NifD) located 3 amino acids away.^102,104,124^ Residues coordinating the COR BchYZ [4Fe-4S] cluster and the predicted ISR [4Fe-4S] cluster are unknown (Figure 10).^104^

### 2.5 Evolution of Nitrogenase and NFL Enzymes

#### 2.5.1 Evolution of NifDK, NifEN, and NFL Systems

In genomes of diazotrophs with Group I, II, and III N_2_ases, all have at least a Group I or Group II MoFe N_2_ase, and in addition may have a Group III VFe or FeFe N_2_ase.^23,81,123,135,136^ For FeMo-co maturation, the *nifE* and *nifN* genes probably originated from an ancient duplication of *nifD* and *nifK*, respectively, or a *nifD* and *nifK* ancestor gene cluster^124,125,137,138^ (Figure 12). The MoFe N_2_ase appears to have originated in archaea, consistent with the apparent origin of NifB in archaea as well. Subsequently, based on the geochemical history of Mo versus V and Fe availability, it is thought that gene duplications and further sequence modifications gave rise to the lower-efficiency alternative VFe and FeFe N_2_ases for N_2_ fixation when Mo is deplete in the environment.^125,139–142^ More recent phylogenetic studies and ancestral sequence reconstruction suggest N_2_ases and their maturases emerged from maturase-like sequences, although the precise functional role of such heterometric ancestors remains speculative.^101,138^ Regardless, these heterometric ancestors are deeply rooted with NFL systems like MAR, where the last common ancestor appears to have the 6 conserved cysteine residues for P-cluster binding, and at least a conserved cysteine and unknown second residue for binding of an L-cluster-like metallocofactor (Figures 10 and 11).^101^ The last common ancestor of N_2_ase and NFL systems like MAR is predicted to have arisen from an ancient predecessor of the extant homodimeric CfbD for F430 cofactor biosynthesis through duplication of an ancestral CfbD-like gene.^101,143,144^ Such duplication and evolution would then give rise to the heterotetrametric N_2_ase and NFL systems. However, whether this duplication event for transition from homodimer to heterotetramer occurred once leading to all extant systems, or happened at two separate events leading to the bacteriochlorophyll oxidoreductases (DPOR and COR) from one event and the other heteromeric NFL systems from another event, requires further investigation.^101^

**Figure 12:**
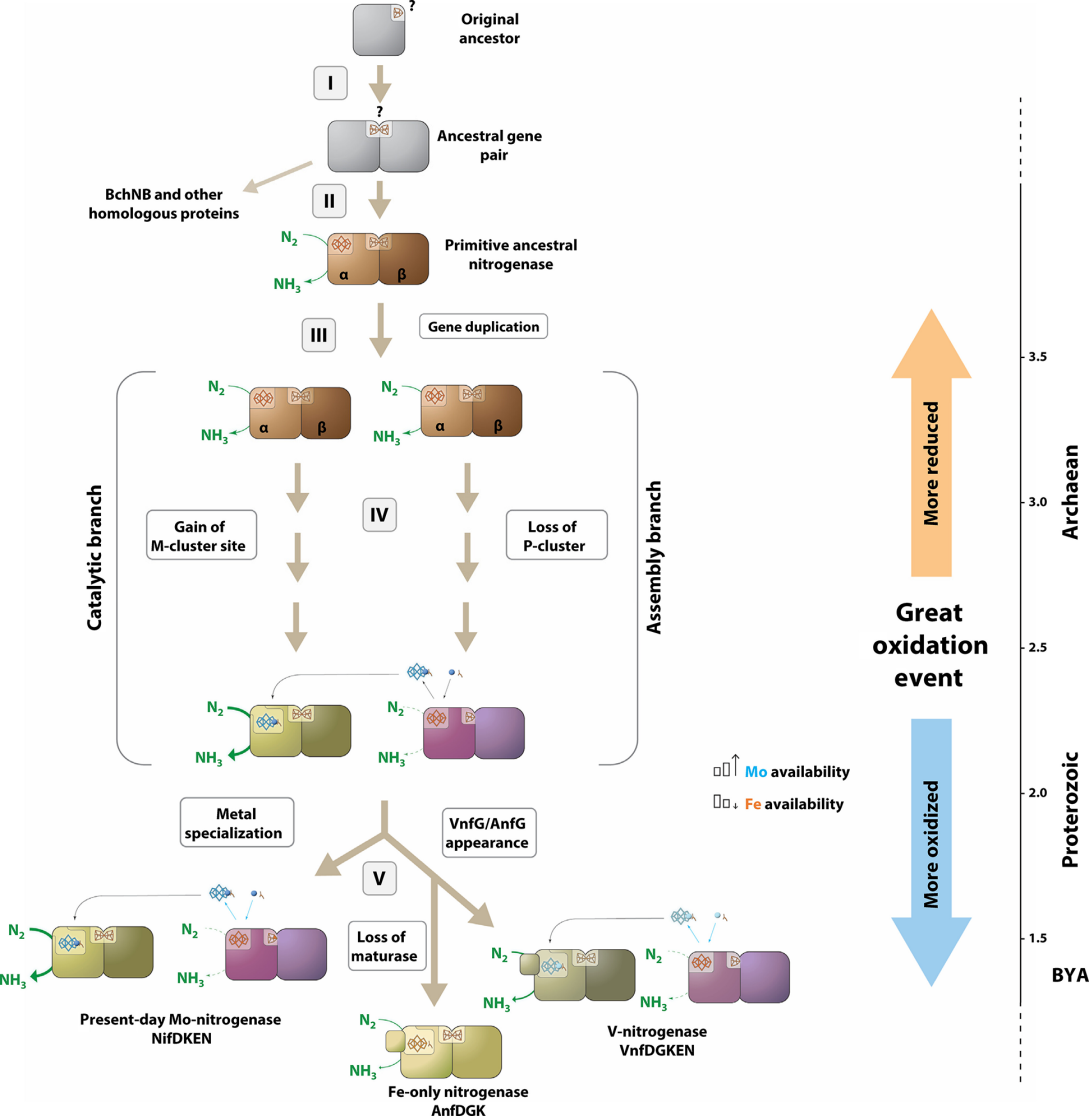
**Proposed evolutionary history of nitrogenase.** An ancestral gene duplication event gave rise to the α and β subunits, and given their closer similarities, the duplication event likely occurred before their divergence into N_2_ase and related proteins (e.g., BchNB) (I). The primitive N_2_ase emerged from the ancestral gene pair, featuring an L-cluster for N_2_ fixation and a P-cluster for electron transport (II). A second gene duplication of the αβ gene pair permitted the exploration of new evolutionary space while allowing improvement of the N_2_-fixing activity (III). One gene copy evolved into a specific maturase for the incorporation of Mo/homocitrate into the L-cluster (assembly branch) and the other copy evolved into an efficient catalyst that enclosed the FeMo-co at a unique reaction site for N_2_ fixation (catalytic branch) (IV). Specialization of N_2_ase and maturase continued after the Great Oxygenation Event (GOE) due to a shift in metal availability, resulting in the appearance of NifDK (the catalytic component of the extant Nif system) and NifEN (the cofactor maturase of the extant Nif system). Subsequently, the Vnf and Anf systems evolved for adaptation to specific metals, with Vnf likely appearing first, followed by Anf from Vnf gene duplication but without duplication of the maturase (V). This proposal places the emergence of N_2_ fixation at least 3 billion years ago, coinciding with the shift of metal availability during the GOE while underscoring the evolutionary advantage and adaptability of early N_2_ases in metal-scarce environments. Reproduced from ref 144. CC BY 4.0.

Duplication of *nifDK* or an ancestral sequence in the evolution of *nifEN* is supported by the observation that the genomes of some diazotrophs are devoid of *nifN* or *nifEN* altogether.^123,128^ It was initially proposed that the significant similarity between NifDK and NifEN probably allows systems only possessing *nifBHDK* to directly assemble FeMo-co or a simpler precursory metallocofactor (i.e., L-cluster) on the NifDK heterotetramer.^128,145^ In support of *in situ* FeMo-co assembly, the purified *A. vinelandii* NifEN heterotetramer with bound L-cluster combined with NifH Fe protein exhibits N_2_ reduction turnover numbers of ∼1.5. When expressed in *E. coli* without any *nifDK* genes, assimilation of ^15^N_2_ into ammonia and biomass by NifH and NifEN is observed.^143,144,146^ Further, recent work with the thermophilic diazotrophic bacterium *Roseiflexus* sp. RS-1, which lacks NifEN, indicates that FeMo-co may assemble *in situ* on the NifDK scaffold. Heterologously expressed and purified NifB, NifH and NifDK protein components of *Roseiflexus* alone can assemble a functional N_2_ase, whose activity is 50-fold higher when Mo is included in the assembly reaction versus Fe only. However, the precise identity of the catalytic metallocofactor has yet to be verified.^131^ Lastly, reconstruction, synthesis, and structural characterization of ancestral N_2_ase sequences indicate that key subdomain motifs distinctive of the extant MoFe, VFe, and FeFe N_2_ases have evolved over time through insertional events into an ancestral N_2_ase or N_2_ase predecessor^130^ (Figure 13). Even the predicted ancestral N_2_ase sequences appear to follow a similar N_2_ reduction mechanism, where concomitant H_2_ liberation is part of the reductive hydride elimination step associated with N_2_ binding and reduction^138^ (Figure 2). While further work is needed, these initial discoveries indicate that certain extant NifDK, and probably ancestral sequences as well, compose an N_2_ase in which FeMo-co is directly assembled on the catalytic scaffold (Figure 12). Ultimately, the question arises as to whether any of the extant or ancient NFL systems that perform alternative reactions directly assemble FeMo-co or similar heterometal-containing metallocofactors on the catalytic scaffold.

**Figure 13:**
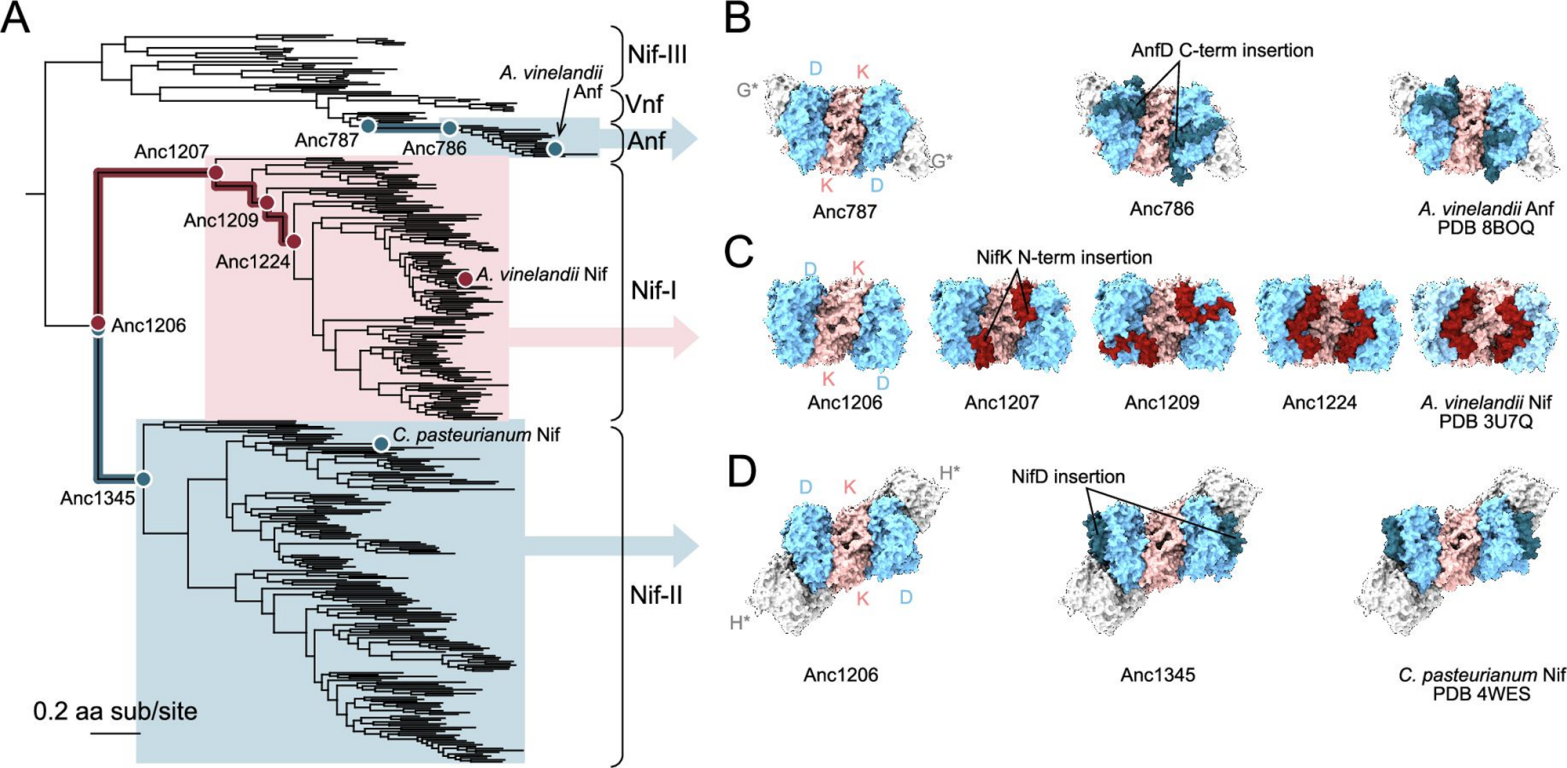
**Variation of nitrogenase structure in the context of sequence phylogeny.** (A) N_2_ase protein sequence phylogeny. Branches and ancestral nodes corresponding to structural insertion events, as well as representative extant variants conserving those insertions, are highlighted and/or labeled. Clade and node colors correspond to the subunit for which an insertion is observed (i.e., blue for the D subunit, red for the K subunit). (B) Elongation of the NifD C-terminus coincident with the origin of the Anf clade. (C) Progressive elongation of the NifK N-terminus through the early evolution of the Group I clade. (D) Insertion within NifD coincident with the origin of the Group II clade. (B–D) All visualized structures are predicted unless otherwise specified with the corresponding Protein Data Bank identifier. Bound G- and H-subunit structures were not predicted together with the NifDK structures and are thus indicated with an asterisk. The binding positions of the G- and H-subunit structures are inferred based on alignment with PDB 8BOQ^61^ and 1M34,^156^ respectively. Reproduced from ref 130. CC BY 4.0.

#### 2.5.2 Evolution of NifB

Phylogenetic analyses suggest that NifB and synthesis of the L-cluster (a.k.a NifB-co) predates N_2_ase systems. Instead, it appears that NifB arose with methyl-CoM reductase (MCR) in the common ancestor of Euryarchaeota, the TACK group (Thaumarchaeota, Aigarchaeota, Crenarchaeota and Korarchaeota) and Asgard archaea.^147^ Subsequently, the “proto” N_2_ase systems which contain *nifBHDK* genes but lack *nifEN* or *nifN*, and subsequently the canonical *nifBHDKEN* systems, appear to have arisen in the Euryarchaeota and transferred therefrom.^139,142,147^ Apparently, such early transfers also gave rise to the bacterial NFL systems, as NifB homologues, albeit of unknown function, are also colocalized with NFL gene clusters.^102,104,106,121,123,124,148^ Consistent with an origin of NifB in archaea, recent structural analysis of the MCR activation complex indicates the presence of L-cluster-like metallocofactors composed of two [4Fe-3S] clusters bridged by three nonprotein ligands and enclosing a central light atom (Figure 3; L-cluster, NifB-co). Two of these L-cluster-like metallocofactors were coordinated by McrC via His49 and His118 for one cluster and Cys55 and Cys12 for the other. The third cluster was coordinated by Mmp7 via Cys143 and His84. However, while McrC has been implicated in the activation of MCR, and while these clusters are poised to form an electron transfer chain to activate F430 by reducing it to the Ni­(I) state, their redox potential and role in electron transfer remain unknown.^147,149^

Also in archaea, many methanogens and alkane-oxidizing methylotrophs lack N_2_ase and NFL systems but possess a *nifB* gene homologue that appears essential for growth and methanogenesis.^147,150^ Even those archaea with Nif gene clusters possess *nifB* elsewhere on the genome.^140,142,151,152^ Although a NifB homologue from a nondiazotrophic methanogen or alkane oxidizing archaea has yet to be characterized, NifB from diazotrophic *Methanosarscina acetivorans* and *Methanobacterium thermoautotorphicum* are functionally competent for synthesis of L-clusters, which can then be further utilized by the NifEN maturase system of *A. vinelandii* for FeMo-co synthesis.^152^ The key difference is that these archaeal NifB homologues do not possess the C-terminal NifX domain of the *A. vinelandii* NifB for FeMo-co trafficking. In *A. vinelandii*, the NifX domain of NifB and the separate NifX protein function to transfer FeMo-co and FeMo-co precursors between nitrogenase-associated proteins for N_2_ase cofactor maturation and insertion.^153,154^ In contrast, diazotrophic archaea possess multiple individual NifX protein homologues for proposed metallocofactor trafficking.^152,155^ As such, much remains to be resolved regarding the function of NifB homologues, the metallocofactors they synthesize, and how the metallocofactors are shuttled around the cell to target bacterial NFL systems and archaeal proteins of unknown identity.

## 3 Nitrogenase Catalysis and Spectroscopic Advancements for the Characterization Thereof

All extant MoFe, VFe, and FeFe N_2_ase possess key conserved structural motifs and emerging underlying catalytic mechanisms for N_2_ fixation. Decades of N_2_ase genetic, amino acid substitution, structural, spectroscopic, and biochemical studies have contributed to the current understanding and knowledge gaps on the assembly, structure, function, mechanism, and regulation of the three N_2_ase isoforms. Undeniably, progress in fundamental N_2_ase understanding has provided an advantageous framework for elucidating the functional and mechanistic underpinnings of the contemporary NFL systems. As enumerated in the Introduction, there are numerous comprehensive reviews on N_2_ase. However, for the purposes of this review, it is instructive to highlight the salient features of N_2_ase structure, function, and catalytic mechanisms as a framework for discussing the functions and key knowledge gaps of the NFL systems in sections 4–9.

### 3.1 Nitrogenase Architecture, Electron Transfer, and Catalysis

#### 3.1.1 Metallocofactor Coordination

Fundamentally, N_2_ases conduct electron transfer along a system of increasingly complex iron–sulfur clusters in a gated manner for N_2_ or alternative substrate reduction. As introduced in section 2.1, the MoFe, VFe, and FeFe N_2_ases each are two-component systems composed of the dinitrogenase reductase Fe protein (component 2) and the catalytic component (component 1) for substrate reduction (Figure 1). All N_2_ase Fe protein homologues possess a single [4Fe-4S] cluster at the dimeric NifH, VnfH, or AnfH interface coordinated by conserved NifH residues Cys97 and Cys132 (*Av* numbering). Four conserved cysteine residues, two from each homodimeric half, coordinate the four Fe ions of the [4Fe-4S cluster] (Figure 14). Throughout this review for consistency with the field, the *Av* NifH, NifD, and NifK sequence numbering will be used as the basis for comparison to other N_2_ase and NFL sequences.

**Figure 14:**
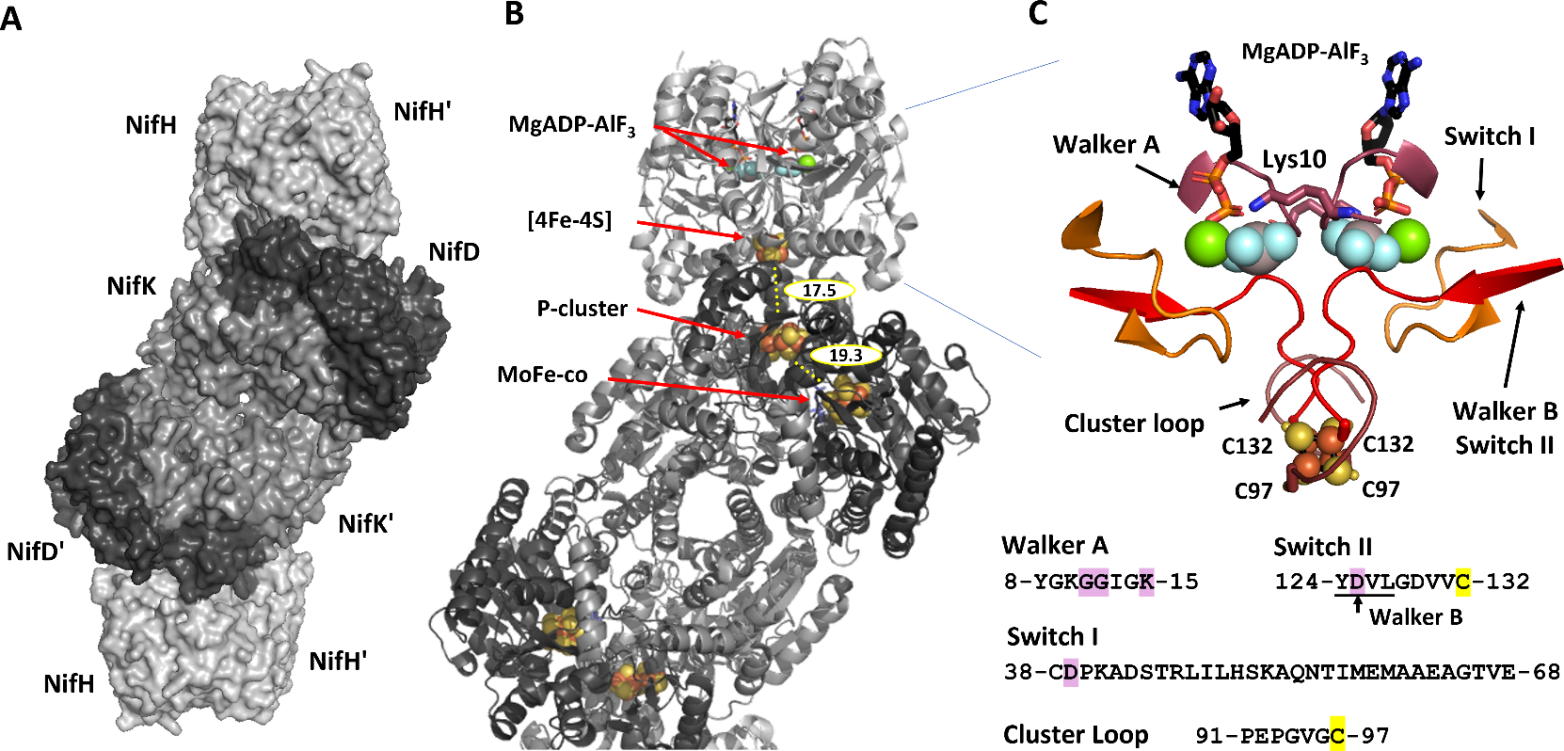
**Structure and metallocofactors of the MoFe nitrogenase.** (A, B) Surface and cartoon representation of NifH Fe protein (component 2) dimers complexed to the catalytic NifDK (component 1) tetramer and stabilized by Mg-ADP aluminum tetrafluoride (Mg^2+^, green sphere; AlF_4_, gray with cyan spheres). Fe ions indicated in orange and S atoms in yellow. Metallocofactor intermolecular distances are given in Å. (C) Zoom-in of [4Fe-4S] cluster and nucleotide coordination by Walker A/B, Switch I/II and Cluster loops of NifH. Amino acid sequence numbering is for *A. vinelandii* NifHDK; yellow residues are for [4Fe-4S] cluster coordinating cysteines; pink residues are nucleotide coordinating residues. PDB accession 1M34.^156^

Within the catalytic N_2_ase NifDK component, the P-cluster Fe ions are coordinated by NifD Cys62, Cys88, and Cys154 and NifK Cys70, Cys95, and Cys153 (Figure 15). These residues are conserved in VnfDK and AnfDK, and likewise function in P-cluster coordination in the respective VFe and FeFe N_2_ase systems. The NifDK FeMo-co is covalently coordinated by only two residues, NifD Cys275 and NifD His442.^157,158^ The NifD Cys275 thiol coordinates the FeMo-co iron Fe1. At the opposite pole of FeMo-co, the NifD His442 residue, which is located on the protein loop between NifD β-sheet #14 and unique left-handed αL-helix #19, covalently bonds with the heterometal Mo through the imidazole ring (Figure 15). Both Cys275 and His442 residues of MoFe N_2_ase are absolutely required for stable incorporation of the FeMo-co and catalysis, as mutations at these positions result in an “apo”-protein lacking FeMo-co. In the alternative N_2_ases, the same conserved cysteine residue of VFe and FeFe N_2_ases coordinate the Fe1 ion of the respective FeV-co and FeFe-co. Likewise, the same conserved histidine residue coordinates the respective heterometal V or Fe of the FeV-co and FeFe-co.^159–162^ Remarkably, even though a different histidine and cysteine pair are used for NFL systems like MAR,^100,101^ coordination of a complex catalytic metallocofactor by a cysteine and histidine residue pairs appears to be a pervasive structural and functional motif throughout the N_2_ase superfamily (Figure 11).

**Figure 15:**
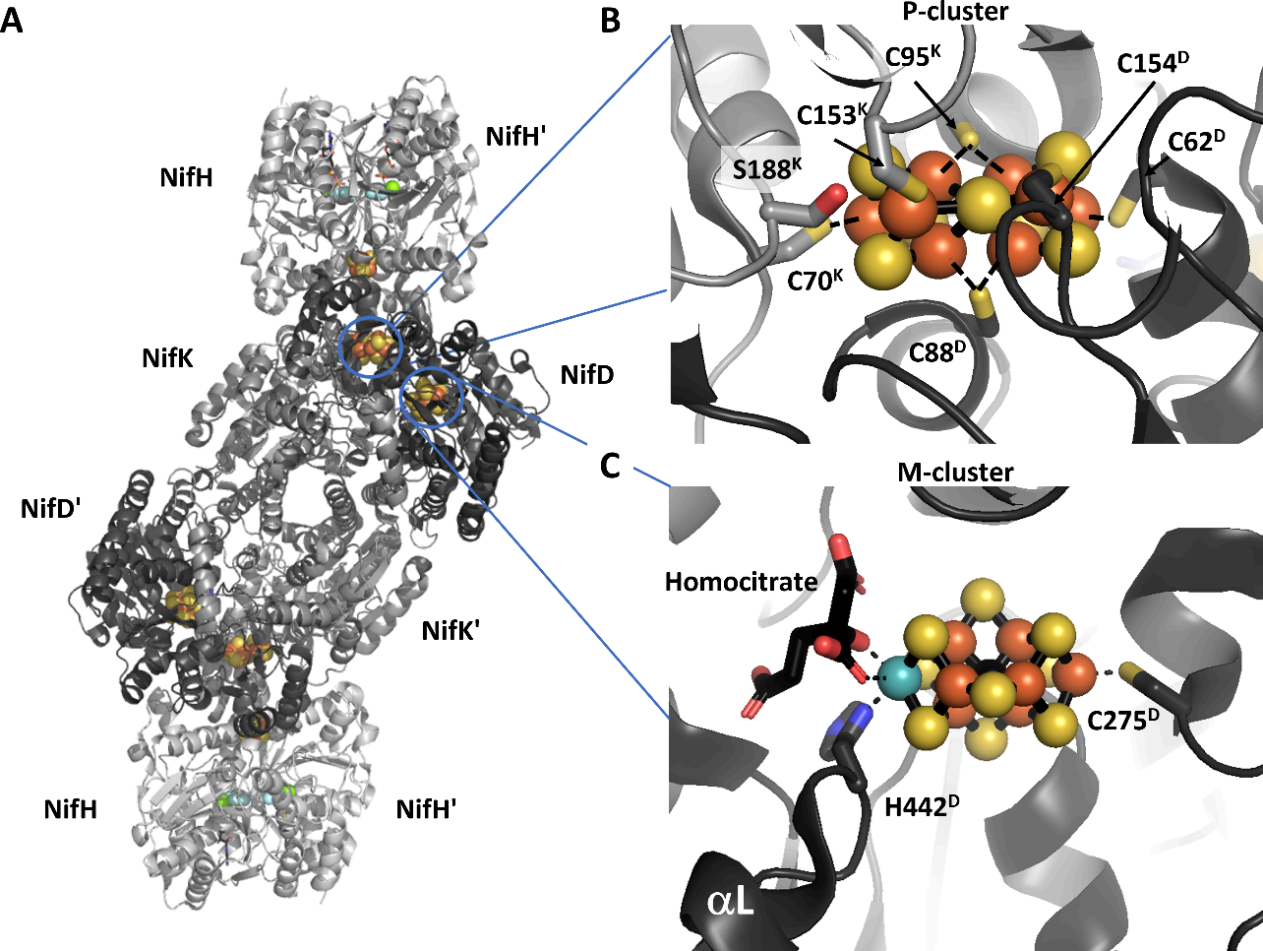
**Primary coordination of MoFe nitrogenase P-cluster and FeMo-co.** (A) Cartoon representation of NifH Fe protein (Component 2) dimers complexed to the catalytic NifDK (component 1) tetramer and stabilized by Mg-ADP aluminum tetrafluoride (Mg^2+^, green sphere; AlF_4_, gray and cyan spheres). Fe ions are indicated in orange and S atoms in yellow. PDB accession 1M34.^156^ (B, C) Zoom-in of P-cluster in the P^N^ state and FeMo-co coordination. D and K superscripts indicate NifD and NifK residues, respectively. PDB accession 1M1N.^184^

In addition to FeMo-co primary coordination by conserved NifD Cys275 and His442 residues, there are multiple polar interactions between NifD residues, the *R*-homocitrate, and the FeMo-co to stabilize metallocofactor binding and ensure proper activity (Figures 15 and 16). The interactions listed below are also conserved in the VnfD and AnfD coordination of the FeV-co and FeFe-co respectively, except where otherwise noted:

1. Two additional polar interactions with the heterometal ion occur through the O5 and O7 oxygen atoms of the *R*-homocitrate molecule.^7,7^ Loss of *R-*homocitrate through deletion of NifV, alkaline treatment, or transiently during catalysis does not result in loss of FeMo-co, but does result in distorted cofactor density and possible rotation of the cofactor about the Cys275–Fe1 bond. In other words, *R-*homocitrate (or *R-*citrate) functions as a “staple” to hold the cofactor in a specific orientation,^10,163,164^ and transient dissociation may explain the observed interconversion of the different Fe-protein [4Fe-4S] and FeMo-co belt sulfurs (S2B, S3A, S5A) during turnover.^165–167^

2. The functional amide group N of conserved NifD Glu191 hydrogen bonds with O1 of *R*-homocitrate.^58,158,168^ In addition to helping coordinate the protein environment near the FeMo-co active site for N_2_ reduction,^3,7^ Glu191 is also observed to be required for maintaining stable incorporation of FeMo-co itself. Mutation of Gln191 to a lysine resulted in N_2_ase proteins with decreased metallocofactor content based on EPR signal intensity.^169,170^

3. On the long-arm of the *R*-homocitrate molecule, O4 is positioned to hydrogen bond to the amine group N of the conserved NifD Lys426 via a water molecule. Substitution of the Lys426 residue (save for K426R mutation) resulted in an enzyme with <50% activity and <50% FeMo-co occupancy compared to the wild type enzyme based on metal analysis and EPR signal intensity.^171,172^ In the VFe and FeFe alternative N_2_ases, an Arg residue replaces NifD Lys426. Based on the NifD L426R substitution showing negligible reduction in activity, the arginine is also implicated in *R*-homocitrate O4 coordination.^59,61,62^ Further, the backbone amide N of the semiconserved Ile425 or Gly424 residues are positioned to bind with *R*-homocitrate O3.^61,164,173–175^ Similarly, substitution of Ile425 resulted in an enzyme with <20% activity.^176^
4. In the N_2_ase resting state, the ε2 amine of conserved NifD His195 is observed to form polar interactions with FeMo-co belt sulfur S2B. Mutation of His195 to asparagine and other residues (except Glu195) result in N_2_ase proteins with decreased metallocofactor content based on EPR signal intensity.^169,170,177^

**Figure 16:**
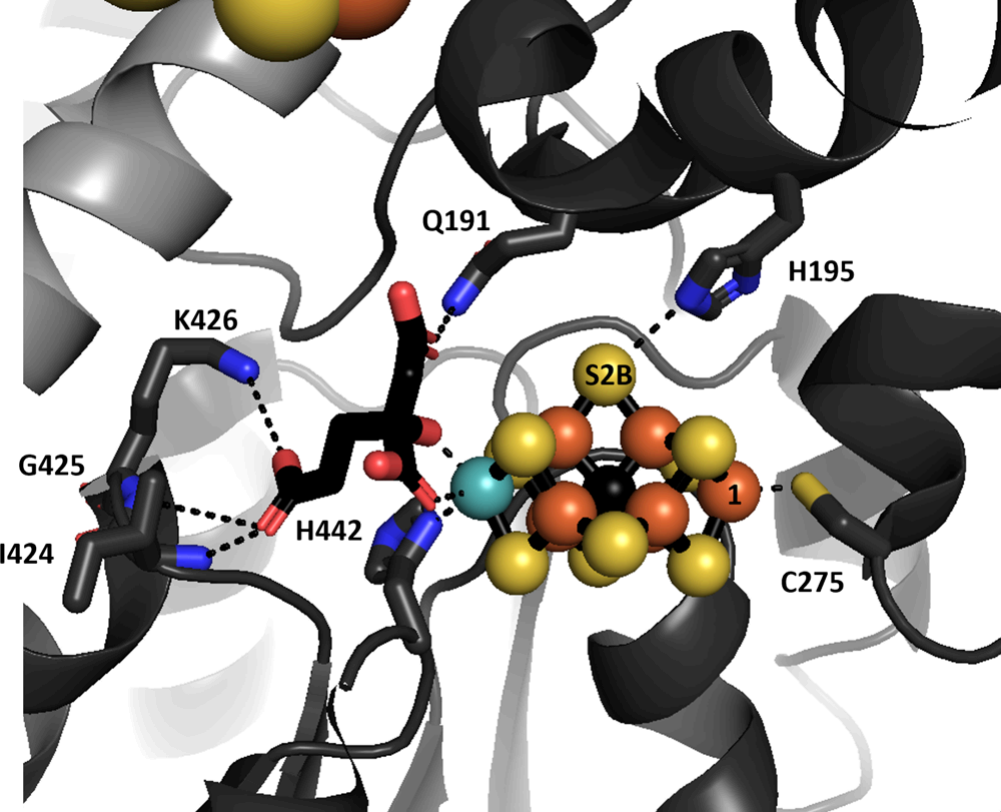
**Nitrogenase residues and homocitrate that are required for stable FeMo-co coordination.** All residues correspond to NifD. PDB accession 1M34.^156^

Thus, interactions between the protein environment and the catalytic FeMo-co are complex and dynamic to enable substrate reduction. Given the observation of complex L-cluster-like metallocofactors and their analogous coordination by a cysteine and histidine pair of residues (Figures 3 and 11), a key question arises as to whether other polar contacts anchor the NFL systems catalytic metallocofactors in a particular orientation to prevent unconstrained rotation about the coordination axis. The residues detailed above for N_2_ase FeMo-co stabilization via additional polar interactions appear to be absent in NFL systems, and work will be critical for understanding the full NFL metallocofactor coordination network for proper cofactor orientation and catalysis.

#### 3.1.2 Electron Transfer in the Nitrogenase P-Cluster and FeMo-co

Initially in the N_2_ase NifDK catalytic cycle, the P-cluster is considered to be in the reduced P^N^ state. The Fe protein in the reduced [4Fe-4S]^1+^ state binds to the NifDK surface, causing a conformational change in the NifDK protein environment in the region of the P-cluster and FeMo-co, which gates the rate limiting transfer of an electron from the P-cluster to the FeMo-co. Specifically, residues NifD Tyr64, NifK Tyr98, and NifK Phe99 are thought to mediate the electron transfer between the P-cluster and FeMo-co based on amino acid substitutions (*Av* numbering).^178^ This initial electron transfer places the P-cluster in the P^1+^ state. Spectroscopic studies indicate that the electron-deficient P^1+^ state is quickly backfilled by an electron from the Fe protein to reduce the P-cluster back to the P^N^ state; a process termed “deficit spending” ^178–183^ (Figures 14 and 17). Transfer of the electron from the Fe protein to the P-cluster oxidizes the Fe protein back to the [4Fe-4S]^2+^ state.

**Figure 17:**
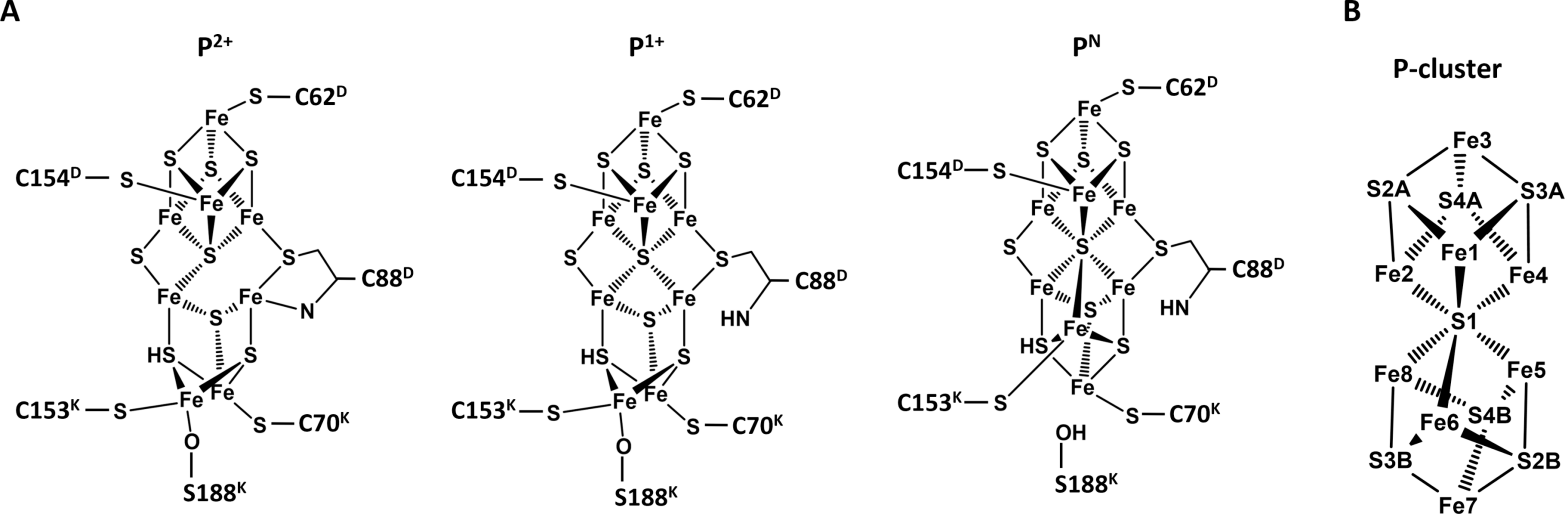
**P-cluster oxidation and reduction states.** (A) Coordination of P-cluster as it switches from the fully reduced P^N^ state to the oxidized P^1+^ state during the nitrogenase NifDK catalytic cycle. In addition, the metastable P^1+^ state can be spontaneously oxidized to the P^2+^ state. D and K superscripts indicate NifD and NifK residues, respectively. (B) Atom numbering of the P-cluster. Structures are based on ref 181.

The binding of the Fe protein and the conformational gating is essential for electron transfer. In the absence of the Fe protein, the P^N^ to P^1+^ redox couple is −0.23 to −0.30 V, whereas the redox couple for the FeMo-co between the resting and reduced state is −0.59 to −0.60 V relative to the standard hydrogen electrode (SHE).^183^ Similarly, the redox couple for the FeV-co and FeFe-co are −0.38 V and −0.40 V, respectively, relative to the SHE.^183^ Therefore, without interactions with the Fe protein, electron transfer from the P^N^ to the FeMo-co does not appear to be thermodynamically favorable. In the presence of nucleotides, the midpoint potential of NifH decreases to −0.43 V for ADP and −0.44 V for ATP relative to the SHE.^185,186^ Similarly, the midpoint potential for NifH from *A. chroococcum* with ADP bound was observed to be −0.45 V versus the normal hydrogen electrode (NHE)^187^ Upon complex formation between NifH and NifDK, the midpoint potential of NifH changes by −0.20 V to yield a thermodynamically favorable −0.62 V for electron transfer from the P-cluster to FeMo-co.^188^ While the P^N^ to P^1+^ transition is the key aspect of N_2_ase catalysis under turnover conditions, the metastable P^1+^ state can be further oxidized to the stable P^2+^ state, requiring multielectron reduction by the Fe protein to return to the P^N^ state (Figure 17). Overall, these electron transfer processes result in a FeMo-co with very low potential electrons that are sufficient to reduce N_2,_ protons, and many other small double- and triple-bond-containing compounds as detailed in section 2.2.

#### 3.1.3 Fe Protein Cycle

Based on sequence identity, it is anticipated that most if not all N_2_ase and NFL Fe proteins carry out a common catalytic cycle to deliver an electron to the catalytic component metallocofactor(s). All sequenced extant N_2_ase and NFL homologues of NifH contain the same conserved Cys97 and Cys132 residues possessed by N_2_ase for coordinating the singular [4Fe-4S] cluster at the homomeric interface for electron transfer^63,102,104,105,121–123^ (Figure 18). In the recently proposed catalytic cycle of N_2_ase based on updated kinetic measurements and differential equation models (^189^ and references therein), the Fe protein is considered to begin with ATP bound to each NifH subunit, and the [4Fe-4S] cluster is in the [4Fe-4S]^1+^ or the all-ferrous [4Fe-4S]^0^ state. Upon interaction of the reduced Fe protein with the catalytic component, only one electron is observed to be transferred irrespective of the [4Fe-4S]^1+^ or [4Fe-4S]^0^ cluster oxidation state per pair of ATP hydrolyzed.^190,191^ After the ATP molecules are hydrolyzed to ADP + Pi, the Fe protein remains bound to the catalytic component. Next, the 2 Pi are released from the Fe protein, which appears to be the rate limiting step of the Fe protein cycle (*k*_pi_ = 25–27 s^–1^). Finally, the oxidized and ADP-bound Fe protein appears to release from the catalytic component whereby it can proceed through nucleotide exchange and reduction to begin the Fe protein cycle again.^191–195^

**Figure 18:**
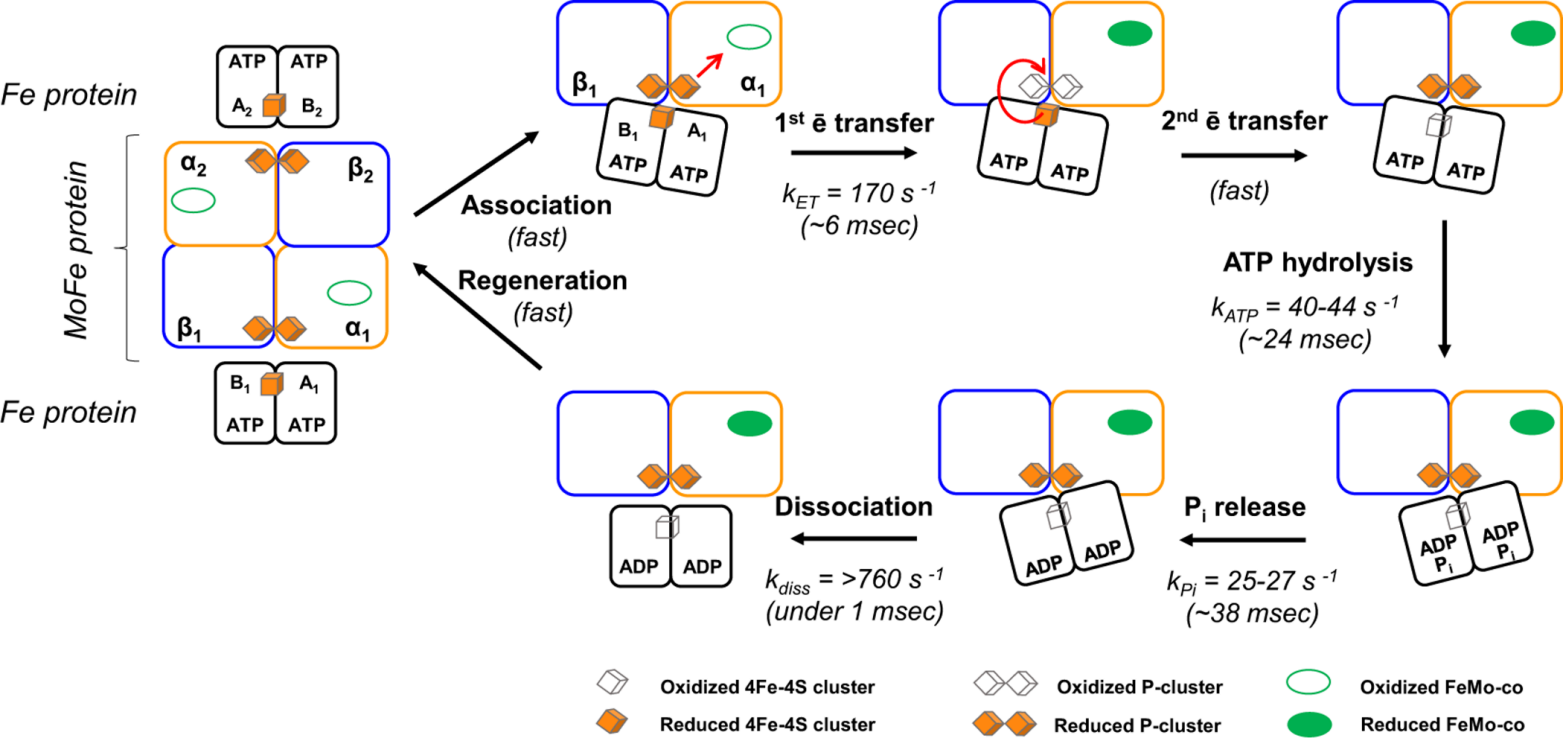
**The Fe protein cycle of nitrogenase.** The scheme shows the steps in the catalytic cycle based on half of the N_2_ase complex. The N_2_ase Fe protein cycle involves transient associations between the reduced, ATP-bound Fe protein and the MoFe protein. It includes electron transfer, ATP hydrolysis, the release of P_i_, and dissociation of the oxidized, ADP-bound Fe protein from MoFe protein. To complete the cycle, the Fe protein needs to be recharged ([4Fe-4S] cluster is reduced) by accepting electrons from an available electron donor. Note that the orientation of the Fe protein depends on nucleotide type. Also, the position of [4Fe-4S] cluster in the ATP-bound Fe protein is closer to the P-cluster than in the case of the ADP-bound form. The kinetic rates are from ref 194. Reproduced from ref 201. CC BY 4.0.

For nucleotide binding and hydrolysis, NifH possesses a Walker A motif (“P-loop”, GKGGIGKS, residues 9–16; *Av* numbering) and Walker B motif (YDVL, residues 125–128; *Av* numbering).^58,124,196,197^ Specifically, NifH is an intradimeric Walker A Protein and a Switch Family Protein in which Lys10 from the adjacent NifH subunit Walker A “P-loop” (residues 9–16) interacts with the bound nucleotide phosphate and stimulates ATP hydrolysis. The Switch I region (residues 39–69) near the nucleotide binding site, and the Switch II region (residues 125–132) near the [4Fe-4S] cluster are involved in signal transduction between the bound nucleotide and [4Fe-4S] cluster.^14,198–200^ Bound ADP-AlF_4_^–^ versus ADP results in NifH conformational changes in the Switch I and II regions that move the [4Fe-4S] cluster approximately 4 Å toward the NifH surface that docks with NifDK.^14,14^ Notably, based on evolutionary tree reconstruction and ancestral protein reconstruction, ancestral N_2_ase NifH proteins also appear to have bound ATP as the catalytic cofactor.^63^

### 3.2 Spectroscopy-Driven Breakthroughs into Nitrogenase Structure, Function, and Assembly

The numerous metallocofactors of N_2_ase have enabled application of a wide array of spectroscopic techniques for the study of structure and mechanism. In conjunction with structural studies and advanced computational analyses, a meaningful correlation has been established between the extensive set of spectroscopic signatures and corresponding electronic and geometric structures for the [4Fe-4S] clusters, P-clusters, and catalytic FeMo-co, FeV-co, and FeFe-co clusters across multiple oxidation states. As many of these studies have been recently reviewed,^20^ here we limit the discussion to new results that showcase the significant utility of a broad range of spectroscopic methods and those that provide a foundation to interrogate the emerging NFL systems (Figure 19). Significant advances have been made using spectroscopy to better understand metallocluster structure in the alternative VFe and FeFe N_2_ases. The biosynthesis and cofactor assembly process have also been the target of many studies. Recent findings on AnfH, AnfDGK FeFe-co, and the NifEN L-cluster are particularly germane to the subject of NFL systems, given the discovery of apparent L-clusters or L-like clusters in Group IV-A NFL systems and the archaeal MCR activation complex.^100,101,147^ Notably, to date there has been no structural, genomic, or spectroscopic evidence for the presence of heterometal atoms or a homocitrate ligand in the active sites of any the NFL systems.

**Figure 19:**
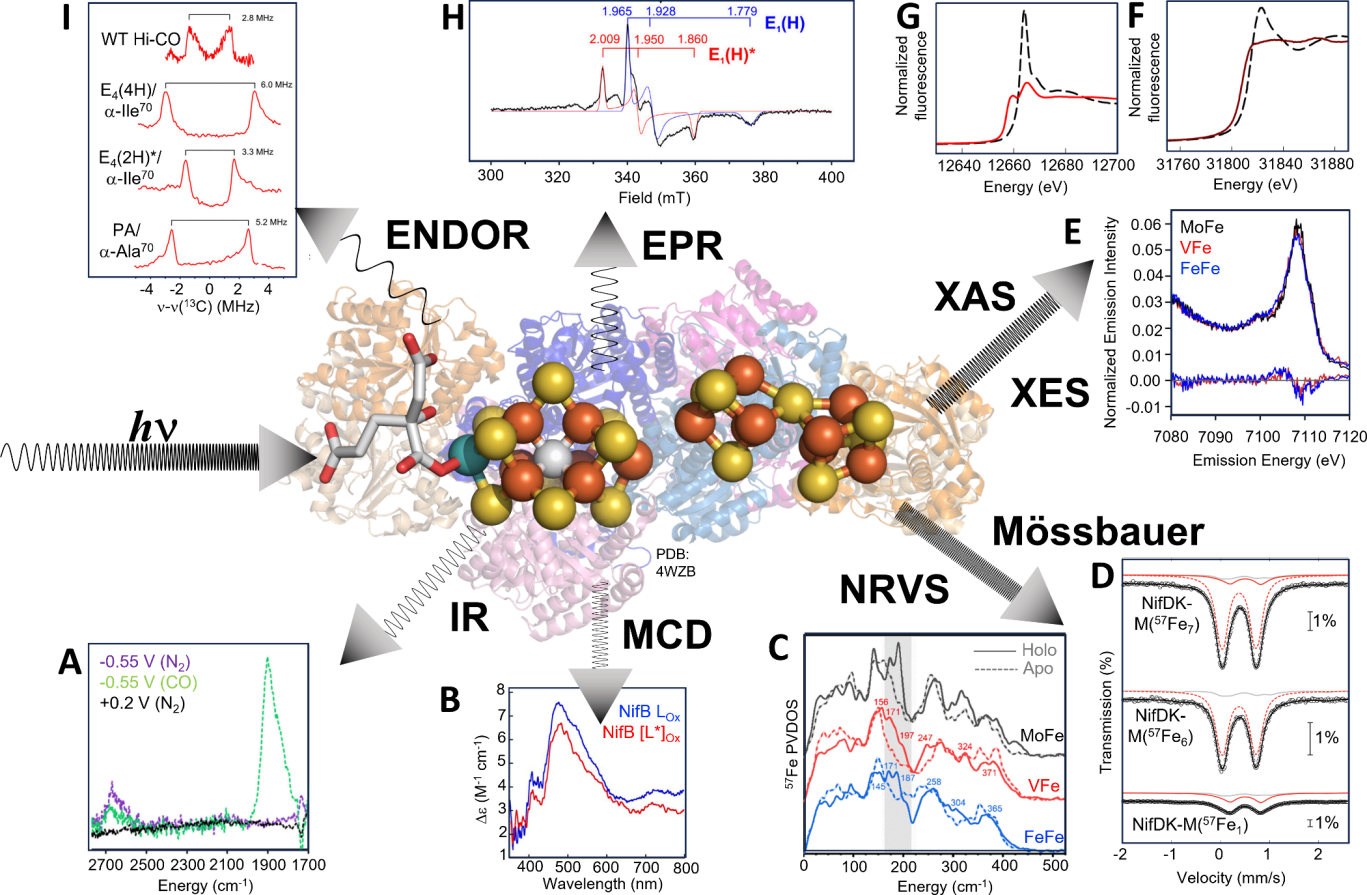
**Recent spectroscopic results on nitrogenases using a range of techniques spanning the electromagnetic spectrum.** (A) Surface-enhanced infrared absorption (SEIRA) spectra of surface-attached NifDK under applied potential in the presence of N_2_ or CO as labeled. Broad bands at ∼2650 cm^–1^ are attributed to the formation of protonated sulfide ligands, while bands at ∼1850 cm^–1^ are attributed to bound CO. These data present clear experimental evidence of protonated sulfide ligands during turnover, providing support for the current mechanistic model. Adapted from ref 205. CC BY 4.0. (B) Magnetic circular dichroism (MCD) spectra of the NifB-synthesized L-cluster and sulfide-deficient L* cluster under oxidizing conditions. The near-identical spectral features suggest sulfide loss occurs readily under oxidizing conditions, with mechanistic implications for cofactor rearrangement. Adapted from ref 206. Copyright 2019 Wiley-VCH. (C) Nuclear resonance vibrational spectroscopy (NRVS) comparing holo and apo forms of the three N_2_ase proteins. Apo-proteins were generated by producing the proteins in a NifB-deficient strain. Pronounced FeMo-co-associated vibrational bands between 170–200 cm^–1^ are attributed to Fe_6_C core “breathing” modes. Adapted from ref 203. CC BY 4.0. (D) Mössbauer spectra of selectively substituted Fe1 in the NifDK FeMo-co shows a higher isomer shift and quadrupole splitting than the average value for the cluster, implicating greater electron density and lower covalency through the bridging sulfides. Adapted from ref 207. Copyright 2023 the authors of ref 207, under exclusive license to Springer Nature. (E) Fe K-edge valence-to-core X-ray emission spectroscopy (XES) of the three N_2_ase catalytic clusters showing nearly identical spectral features, consistent with central carbide ligation. Adapted from ref 202. CC BY 4.0. (F, G) X-ray absorption spectroscopy (XAS) on NifB L-clusters maturated with selenite (G) or tellurite (F) showing reduction and cluster incorporation of the heteroatom. Adapted from ref 67. Copyright 2021 the authors of ref 67, under exclusive license to Springer Nature. (H) X-band electron paramagnetic resonance (EPR) spectrum of AnfDGK + AnfH trapped and frozen under turnover conditions. FeFe-co-derived *S* = 1/2 signals E_1_(H) and E_1_(H)* are suggested to derive from hydride-bound species that are interconvertible in the presence of light. Adapted from ref 208. Copyright 2022 American Chemical Society. (I) Electron–nuclear double resonance (ENDOR) spectra of NifDK selectively labeled with ^13^C at the central carbide site characterized under different reaction conditions. The small isotropic coupling constants indicate little perturbation to the Fe–C bonding or spin coupling scheme across the catalytic cycle. Adapted from ref 209. Copyright 2022 American Chemical Society.

#### 3.2.1 Advances in Resting State Cofactor Geometric and Electronic Structure

In the last 5 years, substantial understanding of the FeFe N_2_ase isoform has emerged. Based on X-ray and structural studies, it was established that a central carbide ion is common to all N_2_ase FeMo-co, FeV-co, and FeFe-co clusters^61,202^ (Figure 19E). This finding is corroborated with nuclear resonance vibrational spectra (NRVS) of all three holo- and apo- N_2_ase isoforms,^203,204^ which show overall similarity in vibrational structure and only slight distinctions in cluster vibrational modes at ∼140–210 cm^–1^ (Figure 19C). These differences have not yet been directly connected to specific vibrational normal modes, but advanced quantum mechanics/molecular mechanics (QM/MM) calculations on the clusters and surrounding protein environment suggest deviations primarily originate from the replacement of a bridging sulfide in FeV–Co with a bridging carbonate ion, and increased symmetry in the FeFe-co. These studies also reproduced the cubane and P-cluster spectra with high fidelity when appropriate secondary sphere interactions were included, highlighting the importance of selecting an appropriate computational model.

Detailed electronic structure determination of FeMo-co in both the resting state and under turnover has been enabled by site selective ^13^C labeling of the central carbide ion via metabolic and genetic manipulation of *A. vinelandii*.^210^ The subsequent pulsed ENDOR studies carried out on ^13^C-labeled FeMo-co indicated exceptionally small isotropic hyperfine coupling to the ^13^C atom, reflecting very low spin density in the carbon 2s orbital (Figure 19I). These studies experimentally validated the resting state cluster spin coupling scheme, with equal numbers of “spin up” and “spin down” Fe ions in the six-iron core around the carbide. In addition, breakthroughs in selective cluster reconstitution were accomplished through postbiosynthetic labeling strategies.^211^ Briefly, the FeMo-co can be extracted from holo-NifDK into organic solvent, and one of the iron centers can be replaced through EDTA chelation followed by reconstitution with excess ^57^Fe. This cluster can be reinserted into apo-NifDK, recovering native levels of activity.^207^ Spectroscopic interrogation of N_2_ase with positionally labeled FeMo-co by ^57^Fe-selective techniques such as Mössbauer and ^57^Fe ENDOR spectroscopy revealed that the unique Fe1 site, presumed to be the iron ion linking the cluster to the protein via Cys275, is more electron rich and exhibits lower metal–ligand covalency than the other belt iron ions in the resting state (Figure 19D). These findings further constrain the broken-symmetry spin coupling schemes that are experimentally supported.

The electronic structure of the FeV-co cluster has also become better understood. Initially, the resting state of FeV-co was thought to comprise an *S* = 3/2 ground spin state on the basis of broad, low-intensity, EPR signals around *g* ≈ 4 observed for the as-isolated enzyme.^212,213^ However, a systematic study has now provided strong evidence that the previously observed signals do not correlate with catalytically active FeV-co.^214^ Instead, the E_0_ resting-state of FeV-co is now thought to be integer-spin, likely *S* = 0, with a valence configuration of [V^3+^4Fe^3+^3Fe^2+^], and the E_odd_ states are half-integer, EPR-active species. Compelling support for this hypothesis was obtained through trapping a state under Ar turnover conditions, which shows well-resolved ^51^V hyperfine coupling consistent with remaining in the trivalent oxidation state, analogous to the seeming redox-inertness of the Mo center in NifDK. The VnfDGK P-cluster electronic structure has also been recently characterized using EPR spectroscopy. For the first time, the oxidized VnfDGK P^2+^ state was shown to have a pronounced feature at *g* ≈ 12 in parallel-mode EPR, with characteristic P^+^ signals under thionine oxidation,^215^ rendering this species very similar to the NifDK P-cluster. These recent EPR signals contrast with prior studies in which the VnfDGK P^2+^ cluster was assigned to be EPR-silent^216^ and, together with earlier work on FeV-co, highlights the importance of coupling spectroscopic, biochemical, and enzymatic characterization when analyzing the often heterogeneous VnfDGK samples.

#### 3.2.2 Insight into the N_2_ase Catalytic Mechanism

Details of the catalytic mechanism for electron accumulation and N_2_ binding have also become more clear in recent years through spectroscopic experiments. EPR studies on the one-electron reduced form of AnfDGK show similar intermediates as NifDK,^208^ implicating a common mechanism for electron and proton transfer and N_2_ binding in which reducing equivalents accumulate on the FeFe-co in the form of hydrides, giving species with *S* = 1/2 spin states (Figure 19H). The central carbide ^13^C ENDOR signals measured across different mutants and under both inhibited and turnover conditions shows very small isotropic hyperfine coupling in all cases, indicative of retaining the cluster spin coupling scheme seen in the resting state and little electronic variation across the carbide during catalysis (Figure 19I).^209^ The invariance of the carbide spectroscopic signals across states has led researchers to propose that the role of the carbide is to provide structural and electronic stability, instead of the electronic and structural flexibility that has been suggested from studies on some N_2_ase model compounds.^217^ Additional structural information on catalytic metallocofactor intermediates has been obtained through surface-enhanced infrared absorption spectroscopy (SEIRAS) performed under electrochemical control (Figure 19A). Clear spectral signatures of nSH (SD) vibrations are observed at low potentials,^205^ indicating protonation of one (or more) bridging sulfide ligands of FeMo-co. Distinct bands are seen in the presence and absence of the inhibitor, CO, suggesting different conformations or populations. To match the spectral signatures to structural models, vibrational frequencies were calculated for different intermediate states, and it was proposed that the signals correspond to protonation of the S2B sulfide ligand in both the bridging and terminal configurations. This spectroelectrochemical experimental evidence for protonated sulfides in the N_2_ase FeMo-co was particularly insightful, and indicated an additional or alternative mechanism of cluster reduction as compared to hydride-bound intermediate formation that has been suggested by EPR for years.^218^ Moreover, the demonstrated application of SEIRAS to large and complex metalloproteins provides opportunities for characterization of NFL systems.

Recent enzyme inhibition studies highlight the importance of the secondary sphere residues in modulating substrate and inhibitor access to the FeMo-co cluster. In particular, the FeMo-co response to CO binding and low-temperature photolysis in the NifD H195Q and V70I mutants has been investigated. The substitutions were found to alter the photolytic behavior of CO, with increased steric bulk changing the rates and speciation of the CO-bound species.^219,220^ In particular, photoinduced CO dissociation in the V70I mutant is significantly slowed, and migration along the cluster has been suggested. Considering the substrate-specific secondary spheres around the NFL active sites and the unusual CO response previously reported for NFL proteins such as MarDK,^100^ mapping analogous CO binding and release dynamics can connect to structural changes during turnover, as reported for NifDK.

#### 3.2.3 Cofactor Assembly

Despite the implicated mechanistic similarities between NifDK, VnfDGK, and AnfDGK, it is now thought that the different N_2_ase isoforms follow distinct cofactor loading and assembly pathways. Specifically, the assembly of the VnfDGK P-cluster has been shown to require the capacity for proper assembly of the FeV-co.^215^ Recent spectroscopic and biochemical evidence indicates that the NifB-deleted strain, which cannot generate the FeV-co, also fails to assemble properly linked P-clusters and instead accumulates immature [4Fe-4S] P-cluster precursors.^215^ This finding of immature P-clusters suggests distinct P-cluster assembly mechanisms in the NifDK versus VnfDGK proteins and underscores the potential fallacy of using “apo”-VnfDGK spectroscopic signatures to isolate contributions from the catalytic site.

Advances have also come in understanding the L-cluster assembly mechanism. The [8Fe-9S-C] L-cluster is suggested to form through the merger of two standard [4Fe-4S] K-cluster precursors on the NifB scaffold. To generate the noncanonical cluster, the radical-SAM enzyme NifB is thought to install the carbide and close the cluster core following 3 sequential deprotonation events, resulting in the [8Fe-8S-C] L* cluster.^221^ Sulfite, instead of sulfide, provides the ninth sulfur atom for completion of the [8Fe-9S-C] L-cluster in the presence of reductant. Sulfide as the sulfur precursor has recently been definitively demonstrated through substitution of sulfite with selenite or tellurite, which generates intact and active L-clusters as confirmed by X-ray absorption spectroscopy and EXAFS analysis (Figure 19F,G).^67^ This ninth sulfur atom is labile and has been suggested to be easily removed under oxidizing conditions, as suggested by EPR and MCD studies on both L* and oxidized L-clusters (Figure 19B).^206^ The flexibility of the belt ligands is important under turnover and inhibitory conditions, where the belt sulfur can be replaced by CO, carbonate, Se, or other potential small molecule substrates or inhibitors. This lability may also be relevant in the NFL systems, where substrates may require access to bind directly to the metallocofactor.

Collectively, over the last 5 years, spectroscopic tools have connected N_2_ase geometric and electronic structures across canonical and noncanonical isoforms of the enzymes. The select accomplishments detailed here offer the potential to develop an insightful relationship between the well-established and relatively well-understood N_2_ase and spectroscopic results obtained from studies on new NFL systems. Ultimately, these signatures provide a roadmap to better understand the structure and mechanism of each NFL enzyme.

## 4 Alternative N_2_ fixation beyond Groups I–III N_2_ases: Group IV-A Nfa

### 4.1 Nfa Identification and Function in N_2_ Fixation

Two instances of potential N_2_ fixation by a member of the NFL family of enzymes have been observed in *Endomicrobium proavitum* (*Ep*) and *Heliophilum fasciatum* (*Hf*). *Ep* possess a single Group IV-A NFL system named NfaBHDK (for nitrogen fixation Group IV-A) and are homologues of NifB, NifH, NifD, and NifK.^105^ *Ep* and closely related bacteria, which do not necessarily possess NfaBHDK, belong to the Elusimicrobia phylum (originally designated as Termite Group 1). Elusimicrobia are a metabolically diverse group of bacteria with members that range from free-living in the soil, to termite gut, cockroach gut, beetle gut, rumen, and endosymbionts and ectosymbionts of protists also living in these environments.^103,222–225^ The Elusimicrobia are part of the Planctomycetes–Verrucomicrobia–Chlamydia superphylum (PVC). Only the *Ectomicrobium* and *Endomicrobium* genera within the Elusimicrobia phylum are observed to have the *nfaBHDK* gene cluster.^224,225^ Of the *Endomicrobium* isolates, *Ep* is the only known free-living species that has been characterized physiologically and metabolically to date.^105^

*Heliophilum fasciatum,* on the other hand, is a photosynthetic clostridium species found in anoxic water-logged soils. It possesses *nfaBHDK* gene homologues as well as four other Group IV NFL systems and DPOR. Importantly, it does not possess any Group I–III N_2_ase genes.^106,107^ Both organisms, when cultured in the presence of ammonium as the sole N-source, exhibited growth, and when cultured on N-free medium with argon as the headspace, exhibited no growth. Strikingly, when cultured on N-free medium with 80:20 N_2_:CO_2_ (*Ep*) or 95:5 N_2_:CO_2_ (*Hf*) headspace, growth was initially observed at the same rate as the ammonium-replete medium, but then growth arrested at a culture optical density at least 2.5-fold lower compared to the ammonium control.^105,107^ Further, during *Ep* and *Hf* growth on N_2_:CO_2_, acetylene reduction activity of the cell culture reached 80–275 (*Ep*) and ∼750 (*Hf*) nmol acetylene reduction per hour per mg total cellular protein (Figure 20). Further, when ^15^N-labeled N_2_ was supplied to *Ep* in the 80:20 N_2_:CO_2_ headspace, it was observed that 85% of the cellular nitrogen originated from the isotopically labeled substrate.^105^ As a comparison, termite gut *Treponema* and *Spirocheta* sp. that possess MoFe N_2_ase exhibit acetylene reduction activities 3–6 times higher than *Ep* when growing diazotrophically on defined media.^226^ Similarly, *H. gestii* and *H. molibis* relatives of *Hf,* which also possess MoFe N_2_ase, exhibit 2–3 times higher acetylene reduction activities.^107^ While purification and enzyme catalytic activity of the *Ep* or *Hf* NfaBHDK system have yet to be reported, these low *in vivo* acetylene reduction activities and only modest growth with N_2_ as the sole nitrogen-source indicate that N_2_ fixation activity is inherently poorer. Such lower activities could be due to an inherently slower enzyme, activity inhibition, or lower Nfa protein abundance compared to Group I–III N_2_ases. Thus, incomplete diazotrophic growth of *Ep* and *Hf* versus relatives possessing bona fide N_2_ase raise the question as to whether N_2_ fixation by Nfa is its primary function or a serendipitous low-activity capability that can only support cell growth at the observed low culture densities for reasons currently unknown.

**Figure 20:**
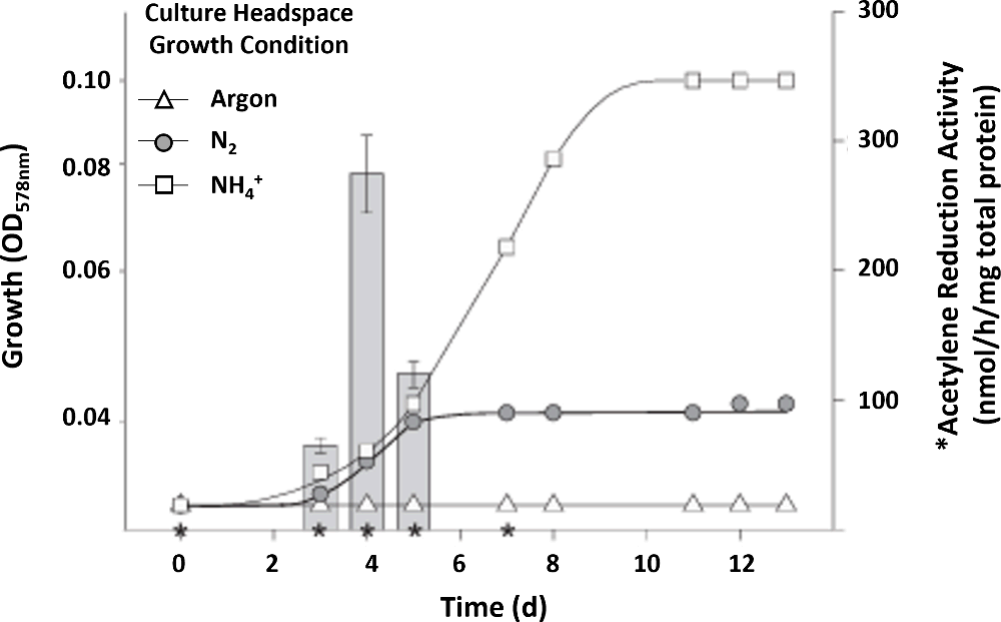
**Diazotrophic growth of *Endomicrobium proavitum*, which only possesses a Group IV-A NFL system, NfaBHDK.** Asterisks indicate time points in which acetylene reduction measurements (bars) were taken. Reproduced from ref 105. Copyright 2015 Society for Applied Microbiology and John Wiley & Sons.

### 4.2 Nitrogen Metabolism beyond N_2_

Beyond initial implications in N_2_ fixation, the substrate specificity and biochemical function of NfaBHDK in bacteria is not fully resolved. Nitrite (NO_2_^–^), nitrous oxide (N_2_O), and hydroxylamine (NH_2_OH) are reduced by N_2_ase to ammonium,^6,227,228^ and it is unknown whether these can also serve as substrates for NfaBHDK. Ammonium, NO_3_^–^, NO_2_^–^, and N_2_O are all common trace compounds present in commercial N_2_ and ^15^N_2_ gases. Previous analyses have shown that per mole of ^15^N_2_ gas, 34 to 1900 μmoles of ^15^N-ammonium, 1.8 to 420 μmoles of ^15^NO_3_^–^/^15^NO_2_^–^, and ≥21 μmol of ^15^N_2_O is detectable in commercial preparations.^229^ For *Ep*, growth with N_2_ headspace to an optical density of 0.04 in a 5 mL culture volume corresponds to 1 μmole of total N that must be assimilated.^105^ This calculation is based on the standard model that 1 L cell culture at an optical density of 1.0 is ∼0.4 g of dry cell weight, and that organic nitrogen composes 14% of the cell mass.^230^ The 10 mL gaseous N_2_ headspace provided to the *Ep* culture would thus contribute a total of 0.06–1 μmoles NH_4_^+^/NO_2_^–^/NO_3_^–^/N_2_O.^105^ While these estimations by no means discount plausible N_2_ fixation by the *Ep* and *Hf* Nfa systems, reduction of NO_2_^–^ and N_2_O to ammonium by these Group IV-A NFL systems could also explain a significant fraction of the assimilated nitrogen and thus growth. Therefore, further biochemical analysis of the Nfa systems is required to determine its contribution to N_2_ fixation versus reduction of alternative substrates contributing to the total nitrogen metabolism and apparent diazotrophic growth of the cell.

### 4.3 Structure of Nfa Systems

Regarding structure, only the primary amino acid sequences for NfaBHDK are known, which share 45%, 41%, 25%, and 22% identity with *A. vinelandii* NifBHDK, respectively.^105,231^ As introduced in section 2.3.1, NfaH contains the conserved Cys97 and Cys133 residues possessed by NifH (Cys96 and Cys132, *Av* numbering) for [4Fe-4S] cluster binding to transfer an electron to the proposed NfaDK catalytic heterotetramer. In addition, NfaH possesses the conserved Walker A (P loop) and Walker B motifs for ATP binding,^58,124,196,197^ along with Switch I and II domains for conformational changes upon ATP hydrolysis.^198,200^ For electron transfer, NfaDK possesses the same 6 conserved cysteine residues N_2_ase uses for P-cluster coordination (Figure 10), these are NfaD Cys Cys53, Cys78, Cys146, and NfaK Cys13, Cys37, Cys97 (*Ep* numbering). In addition, there are several NfaD cysteine residues also near these conserved residues, which are NfaA Cys50, Cys60, and Cys81. Further structural work is needed to determine if these cysteine residues coordinate two [4Fe-4S] clusters, a P-cluster, or some alternative metallocofactor, and if the cluster is dynamically coordinated by these residues during maturation and catalysis.

For coordination of a potential catalytic metallocofactor, NfaD possesses the conserved Cys257 residue that N_2_ase uses for FeMo-co coordination (NifD Cys275; *Av* numbering) and *R. rubrum* (*Rr*) MAR uses for mar2-cluster coordination (MarD Cys270; *Rr* numbering) (Figure 11). Further, in lieu of the N_2_ase NifD His442 residue (*Av* numbering) for coordinating the FeMo-co, FeV-co or FeFe-co heterometal across all N_2_ase isoforms,^59–62,163,165^ NfaD possesses the same conserved histidine residue other Group IV NFL systems like MAR possess for catalytic cofactor binding (Figure 11; MarD His428; *Rr* numbering). In support of a complex L-cluster or L-like catalytic metallocofactor in Nfa systems, NfaB possesses all the conserved motifs of NifB involved in NifB-co (L-cluster) synthesis. In total, while the identity and location of the Nfa catalytic metallocofactor is unknown, this sequence conservation suggests the presence of an L-like cluster within NfaD coordinated by the cysteine/histidine pair corresponding to MarD Cys270 and MarD H428 (*Rr* numbering).^102,105^

### 4.4 Regulation of Nfa and Nitrogenase

#### 4.4.1 Nfa Regulation by Available Nitrogen Source

At the regulatory level, the Group IV-A NFL system of *Ep* is implicated in nitrogen metabolism. When cultured with a mixture of ammonium and ^15^N_2_ as the N-source, acetylene reduction activity and ^15^N-assimilation into cell mass was inhibited by nearly 100%. Transcriptional analysis demonstrated that the *Ep* Group IV-A *nfaH* gene was only produced in the absence of ammonium.^105^ Nitrogen-based Nfa gene regulation is further supported by *Ep* genome analysis, which indicates the presence a Gln B/Gln K family PII-like signal-transduction protein.^231^ As such, Nfa is the first NFL system implicated to be regulated for the maintenance of nitrogen homeostasis of a cell. Further research is required to determine if additional regulatory mechanisms are involved at the protein levels like those discussed below, which have been a hallmark for stably operating the ATP- and electron-demanding N_2_ase within a cell.

#### 4.4.2 Nitrogenase Transcriptional Regulation by PII

In diazotrophic bacteria, PII proteins regulate N_2_ fixation by binding or releasing from their protein targets based on the urididylylation state of the PII protein (uridine monophosphate, UMP, post-translational modification). PII urididylylation is regulated by bifunctional Gln D (Figure 21). Low cellular glutamine and high α-ketoglutarate concentrations, indicative of insufficient nitrogen availability to the cell, stimulates Gln D urididylyl-transferase activity to urididylylate UMP (PII-UMP). Conversely, high cellular glutamine levels and low α-ketoglutarate concentrations, indicative of sufficient nitrogen assimilation into the cell, stimulate Gln D urididylyl-hydrolase activity to remove UMP from PII-UMP.^232,233^

**Figure 21:**
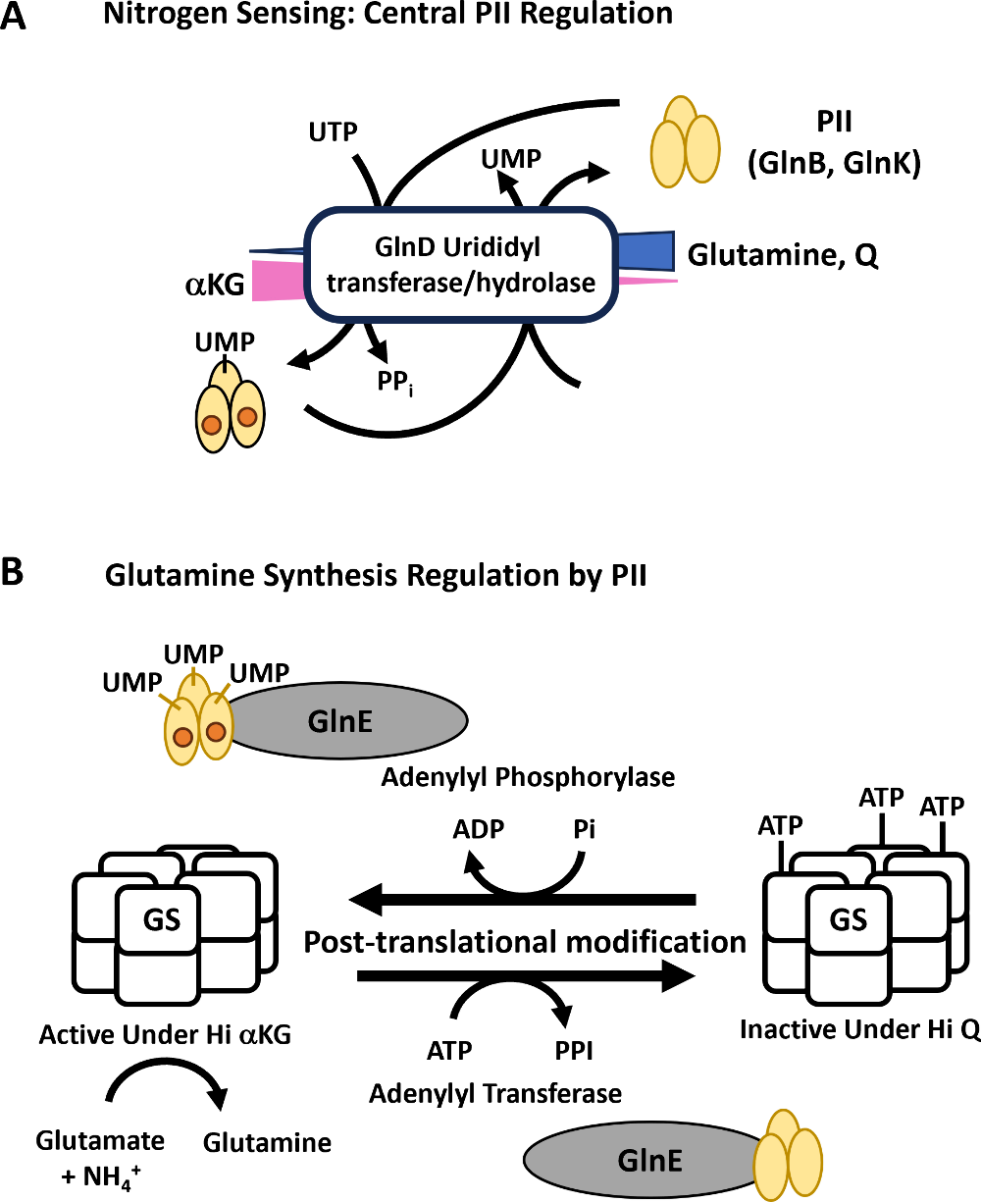
**Regulation of nitrogen metabolism by PII enzymes.** (A) Gln D monitors cell nitrogen status through binding of glutamine (Q) and α-ketoglutarate (αKG), which leads to urididylylation (UMP) of PII. (B) The PII urididylylation state regulates Gln E adenylyltransferase activity versus adenylyl phosphorylase activity to add and remove the adenylyl (AMP) modification from glutamine synthetase (GS), respectively.

For N_2_ase regulation, PII is involved both at the transcriptional level and at the protein level through post-translation modification of NifH. For transcriptional regulation, PII in the unmodified state due to sufficient available nitrogen binds to the NifL sensory protein, which in turn binds to the NifA transcriptional regulator. The PII-NifL-NifA complex results in an inactive NifA transcriptional regulator and prevents *nif* gene expression. When nitrogen availability for the cell becomes limiting, the resulting PII-UMP releases NifL, and NifL in turn releases NifA to activate *nif* gene expression from the *nif* gene promoter^234,235^ (Figure 22C).

**Figure 22:**
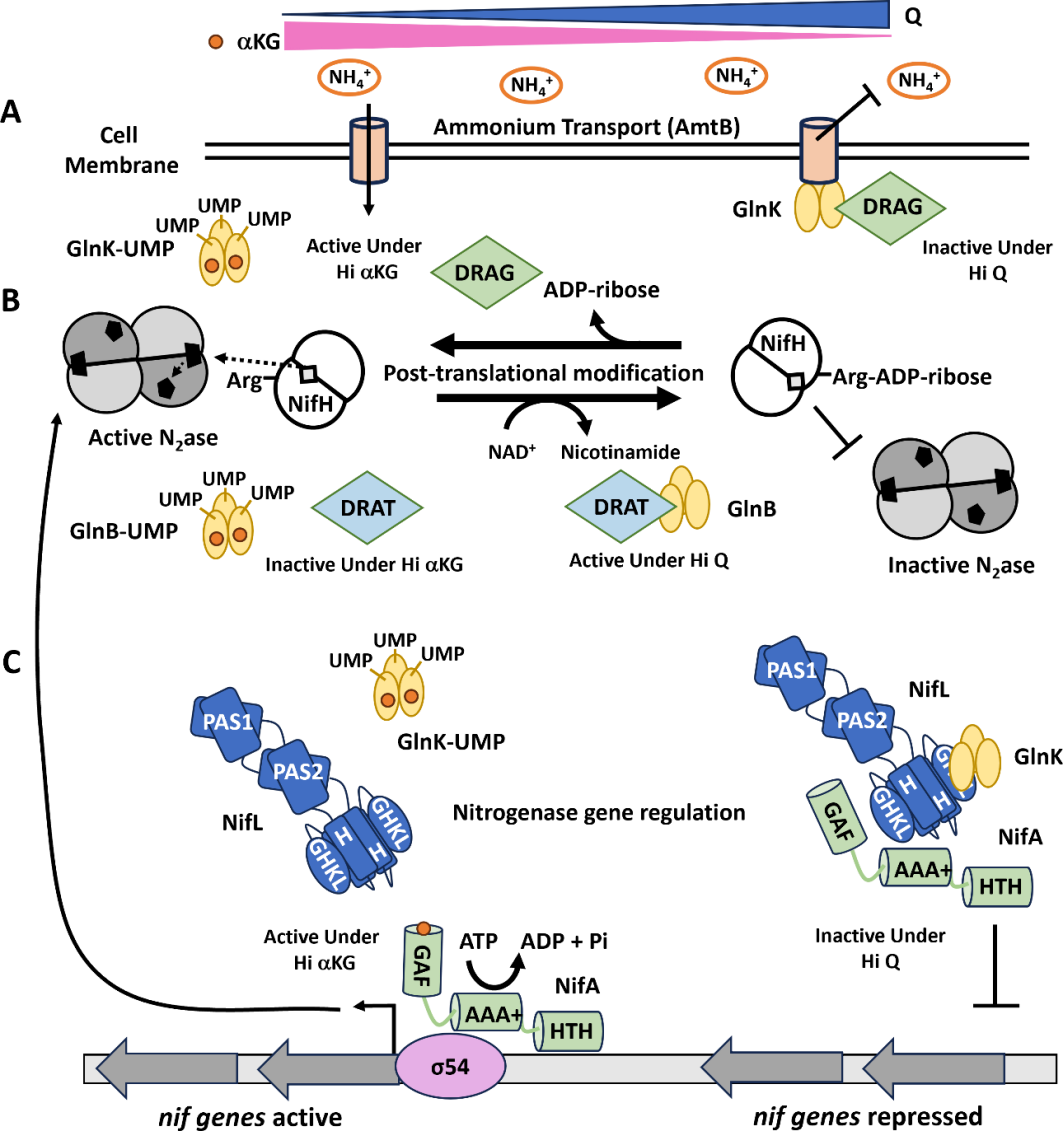
**Regulation of ammonium transport and nitrogenase activity.** The PII urididylylation state (A) regulates ammonium uptake by AmtB ammonium transporters, (B) regulates nitrogenase activity through modulating dinitrogenase reductase activating glycohydrolase (DRAG) and dinitrogenase reductase ADP-ribose transferase (DRAT) activities to remove and add ADP-ribosylation from NifH, respectively, and (C) regulates nitrogenase gene transcriptional activation through altering the binding properties of NifL and NifA two-component regulatory system.

#### 4.4.3 Regulation of the Nitrogenase Fe Protein

For potential regulation of the Fe protein cycle and electron transfer, all N_2_ase and NFL system Fe protein homologues possess a conserved Arg100 residue (*Av* numbering) except ChlL/BchL.^102^ NifH Arg100 is located on conserved helix 4. NifH Arg100 interacts with NifD helix 5 and NifK helix 9 during NifH association with NifDK, and is required for catalysis.^199,236^ This arginine residue is modifiable by NAD-dependent ADP-ribosylation via dinitrogenase reductase ADP-ribosyl-transferase (DRAT) to prevent NifH from complexing with NifDK during times of ammonia availability and/or energy deficiency^237–240^ (Figure 22B). Subsequently, the ADP-ribose post-translational modification is removed by dinitrogenase reductase activating glycohydrolase (DRAG). Notably, DRAT and DRAG are primarily found in the phototrophic alphaproteobacteria (e.g., *R. rubrum*, *R. palustris*, *R. capsulatus*), in part to prevent deleterious N_2_ase activity during transient fluctuations when light and thus ATP become limiting. DRAG and DRAT can also be found in some betaproteobacteria, like *Azoarcus* sp. However, they are not found in the model diazotrophic gammaproteobacteria *A. vinelandii*.^241,242^ DRAT and DRAG can act on NifH and the VnfH and AnfH alternative N_2_ase Fe proteins as substrates within the same organism,^243–247^ and can cross-react with Fe proteins from other species including *A. vinelandii*.^241,248^

There are no known reports of DRAG and DRAT acting on NFL systems such as DPOR and COR, and the factors that determine specificity of Fe proteins as substrates are not fully resolved. The key NifH Arg100 alone does not define specificity, as substitutions to this residue alters NAD cleavage activity by DRAT but does not alter DRAT binding to NifH.^249^ Similarly, substitutions to NifH residue Arg140 also result in the loss of ADP-ribosylation activity but do not alter DRAT binding to NifH. These results indicate that the Arg100 and Arg140 residues are required for DRAT to carry out its catalytic mechanism but not for DRAT binding.^250^ In contrast, NifH recognition and binding by DRAT appears to involve specific NifH conformational states of the Fe protein catalytic cycle. Substitutions of residues within the Switch I region such as NifH D43N results in a variant that binds but cannot hydrolyze ATP. Further, the NifH D43N variant does not serve as a substrate for NifH Arg100 ADP-ribosylation by DRAT.^250^ Similarly, substitution P135F near the Switch II region alters the [4Fe-4S] cluster coordination region and raises the midpoint potential by +0.05 to +0.12 V, also resulting in a NifH variant that cannot hydrolyze ATP and does not serve as a substrate for Arg100 ADP-ribosylation.^250–253^ Given that the NifH protein surface with Arg100 must interact with NifDK and DRAT during their respective catalytic activities, these structural and mutational analyses indicate that DRAT likely recognizes a particular conformational state of NifH after ATP hydrolysis has occurred, consistent with the role of DRAT in downregulating NifH activity when ATP is limiting. Whether NflH homologues adopt a conformation that serves as a substrate for DRAT is entirely unknown and requires further investigation.

In archaea and potentially in some bacteria, regulation of the Fe protein cycle by PII proteins appears to be quite different. NifI_1_ and NifI_2B_ form two subfamilies of PII proteins, whereas all other known PII proteins, including Gln B and Gln K, are part of a distinct third subfamily.^254^ Genes for *nifI*_1_ and *nifI*_2_ are present in the *nif* operons of all N_2_-fixing archaea as well as some anaerobic N_2_-fixing Bacteria, including *Chlorobium tepidum*, *Dehalococcoides ethanogenes*, *Heliobacterium chlorum*, some *Clostridia* sp. and *Desulfitobacteria* sp.^254,255^ In the diazotrophic archaeon, *Methanococcus maripaludis*, both genes are necessary for N_2_ase switch-off in the presence of ammonia.^256^ Mechanistically, NifI_1_ and NifI_2_ appear to bind to each other as an I_1_I_2_ complex and then bind to NifDK under N-replete conditions to inhibit NifH association and thus N_2_ase activity. Further, the I_1_I_2_ complex appears to coordinate multiple NifDK complexes, resulting in larger albeit unresolved structure of 500–800 kDa.^257^ Addition of α-ketoglutarate, which for PII proteins signals nitrogen-limiting conditions, relieved N_2_ase inhibition by NifI_1_ and NifI_2_. The binding of I_1_I_2_ suggests a competitive inhibition mechanism distinct from post-translational modification mechanism of NifH Arg100 in other bacteria.^257,258^

#### 4.4.4 PII Function beyond Nitrogenase

While the presence of PII-family regulators in *Ep* suggests a possible role in *nfaBHDK* gene regulation in response to nitrogen availability, PII-family proteins have broad regulatory functions that are not limited to N_2_ase. Further, no PII proteins are observable in the genome of *Hf*, indicating its NFL systems are regulated by alternative mechanisms, if any.^106^ Specifically, PII proteins like Gln K and Gln B are also involved in regulating assimilation of ammonium by glutamine synthetase (Gln A) and regulating ammonium uptake through interactions with the ammonium transporter (AmtB) (Figure 22A). Genes for AmtB and Gln A are also found on the *Ep* and *Hf* genomes.^105,106^ PII proteins can regulate glutamine synthetase activity through interactions with Gln E, which is a bifunctional Gln A adenylyltransferase and Gln *A* adenylyl phosphorylase for posttranslational regulation of glutamine synthetase activity (Figure 21). When there is sufficient cellular nitrogen, PII binds Gln *E* and stimulates Gln *E* adenylyltransferase activity to add an AMP post-translational modification onto a conserved Gln *A* Tyr397 residue (*E. coli* numbering). Gln A-AMP exhibits downregulated glutamine synthetase activity to prevent unnecessary ATP usage. Conversely PII-UMP, which is synthesized when the cellular nitrogen availability becomes poor, cannot interact with Gln E. The dissociation of PII from Gln E stimulates Gln E phosphorylase activity to remove AMP from Gln *A* and thus upregulate glutamine synthetase activity. Further, PII proteins regulate transcription of the *glnA* gene through interactions with the sensory histidine kinase NtrB. NtrB cannot bind PII-UMP, and unbound NtrB thus phosphorylates NtrC transcriptional regulator to activate *glnA* expression. Conversely, NtrB bound to PII dephosphorylates NtrC, preventing *gln A* expression.^259,260^

Regarding ammonium transport, PII directly binds to the AmtB ammonium transporter when cell nitrogen availability is sufficient to control ammonium uptake. Prevention of excess accumulation of ammonia in the cell in turn prevents excess ammonia assimilation by GS and thus unnecessary ATP usage (Figure 22A). When the cellular nitrogen status becomes poor, PII urididylylation to form PII-UMP causes PII-UMP to release from AmtB and allows more ammonium influx into the cell if available.^261–264^ In total, regulation of nitrogen metabolism by PII proteins is complex^259^ and PII signaling has been integrated by various bacteria to control ammonium metabolism, to control N_2_ fixation via N_2_ase, or both. Thus, there is much still to be resolved about the regulatory mechanisms of Group IV-A NFL systems in response to nitrogen availability.

## 5 NFL Systems for Chlorophyll Synthesis: Group V DPOR and COR

### 5.1 (Bacterio)­chlorophyll Biosynthetic Pathways

NFL oxidoreductases are an integral part of energy metabolism in phototrophic bacteria, algae, fungi, and plants through participation in multiple steps of chlorophyll (Chl) and bacteriochlorophyll (Bch) synthesis. Phototrophic organisms harvest light for ATP synthesis (photophosphorylation) through membrane-associated pigment-containing structures called light-harvesting (LH) complexes. The LH complexes are highly organized protein-pigment complexes containing chlorophyll (*a*, *b*, *c*_1_, *c*_2_, *d*, and other naturally occurring forms), bacteriochlorophyll (*a*, *b*, *c*, *d*, *e*, *g*), and carotenoids.

There are multiple in-depth reviews regarding (bacterio)­chlorophyll biosynthesis and the structure and/or function of DPOR and COR.^7,265–269^ Thus, herein we only review the most salient features of the (bacterio)­chlorophyll biosynthetic pathways and DPOR and COR NFL systems for (bacterio)­chlorophyll synthesis for comparison with other NFL systems. Synthesis of (bacterio)­chlorophyll begins with protoporphyrin IX,^270^ as does heme cofactor synthesis (not shown) (Figure 23). Notably, protoporphyrin IX is synthesized from uroporphyrinogen III, which is the common precursor of the major tetrapyrrole-base cofactors: cobalamin (B12), F430, heme, and (bacterio)­chlorophyll. The biosynthetic pathway for Bch *a* and Bch *b* diverges due to distinct reactions catalyzed by chlorophyllide *a* oxidoreductase COR isoforms a-COR and b-COR^114^ (Figures 5 and 23). As introduced in section 2.3.3, a-COR has dual functions: it can perform the 8-vinyl reduction of 8-vinyl-chlorophyllide *a* to chlorophyllide *a* (Chlide), replacing the need for Divinyl Reductase (DVR), and it can catalyze the C7=C8 double bond reduction of Chlide to form 3-vinyl-bacteriochlorophyllide *a* as a product (Figure 5).^114,115,271,272^ Conversely, b-COR from Bch *b*-producing *B. viridis* recognizes only 8 V-Chlide *a*, not Chlide, as its substrate and catalyzes the direct formation of 3-vinyl-bacteriochlorophyllide *b*, also known as bacteriochlorophyllide *g*.^114,116^ As such, due to the broad substrate specificities of COR DPOR and other enzymes in the (bacterio)­chlorophyll biosynthetic pathways, the sequence of reactions varies from species to species, and may even diverge and converge within the same organism (Figures 4 and 5). Lastly, the a-COR and b-COR share 89% amino acid identity, indicating only a few key amino acid differences result in their distinct respective functions. However, given a structure for a-COR or b-COR BchXYZ has not been determined, the structure–function relationship driving substrate recognition is unknown.^114,116^

**Figure 23:**
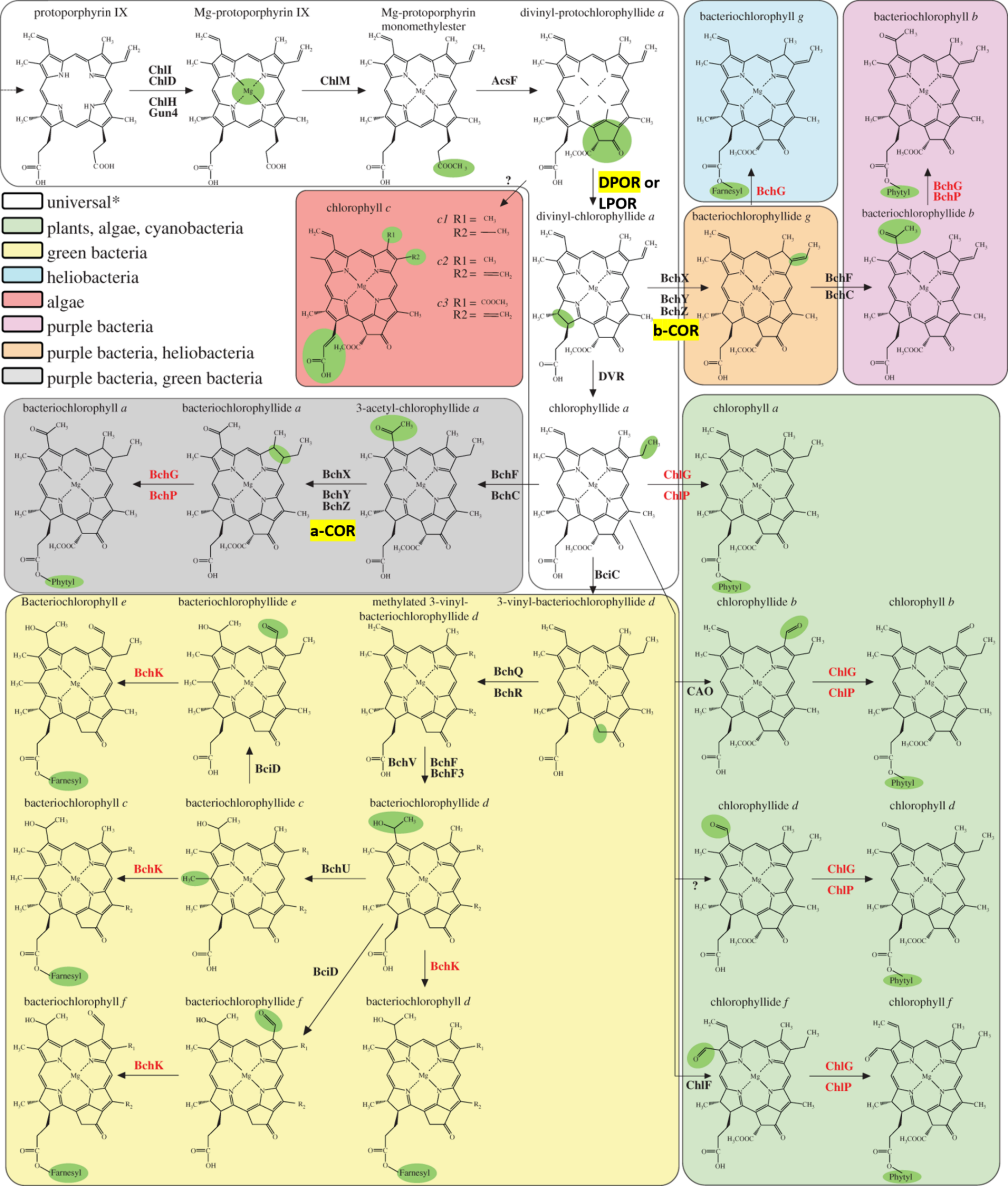
**Biosynthetic pathways for (bacterio)­chlorophyll.** Highlighted are the roles of the NFL dark-operative protochlorophyllide oxidoreductase (DPOR) and the two chlorophyllide oxidoreductase isoforms a-COR and b-COR. Note that in oxygenic plants, algae, and prokaryotes, the light-operative protochlorophyllide oxidoreductase (LPOR) is alternatively expressed instead of DPOR during oxygenic and light conditions (section 2.3.3; eqs 8 and 9). Modified from ref 270 to show DPOR and COR reactions. CC BY 4.0.

### 5.2 Function and Structure of DPOR and COR

As with other N_2_ase and NFL systems, DPOR and COR are two-component systems consisting of a homodimeric Fe protein (ChlL/BchL or BchX) and a tetrameric catalytic component of (ChlNB, BchNB or BchYZ) in an α_2_β_2_ assembly. Atomic resolution structures for DPOR in the complexed quaternary state or for BchL and BchNB separately have been acquired for *Cereibacter sphaeroides* (formerly *Rhodobacter sphaeroides*),^273–275^ *Rhodobacter capsulatus*,^134^ *Thermosynechococcus vestitus*,^276^ and *Prochlorococcus marinus*.^277^ The *Cereibacter sphaeroides* DPOR trapped in the turnover state with ADP-trifluoroaluminate (ADP-AlF_3_) shows that the BchL [4Fe-4S] cluster, the BchNB [4Fe-4S] cluster, and the 8-vinyl-protochlorophyllide *a* substrate reside in a straight line with 17–19 Å intramolecular spacing for electron transfer (Figure 24) (PDB 2YNM;^277^). Notably, the substrate is located in a pocket which otherwise in N_2_ase is the FeMo-co active site region. However, in DPOR and presumably COR, this region is modified such that that they bind and stereospecifically reduce their respective substrates.^134,271^ Mutagenesis studies indicate that the BchB residues Asp290 and Leu426 (*Prochlorococcus marinus* numbering) are required for substrate binding and reduction. While the precise mechanism is not known, it is thought that BchB residues Arg48, Asp290, and His394 form a hydrogen bonding network with the substrate and potentially interstitial water molecules for the reduction of the C=C double bond at positions 17 and 18 (Figure 24B, cyan).

**Figure 24:**
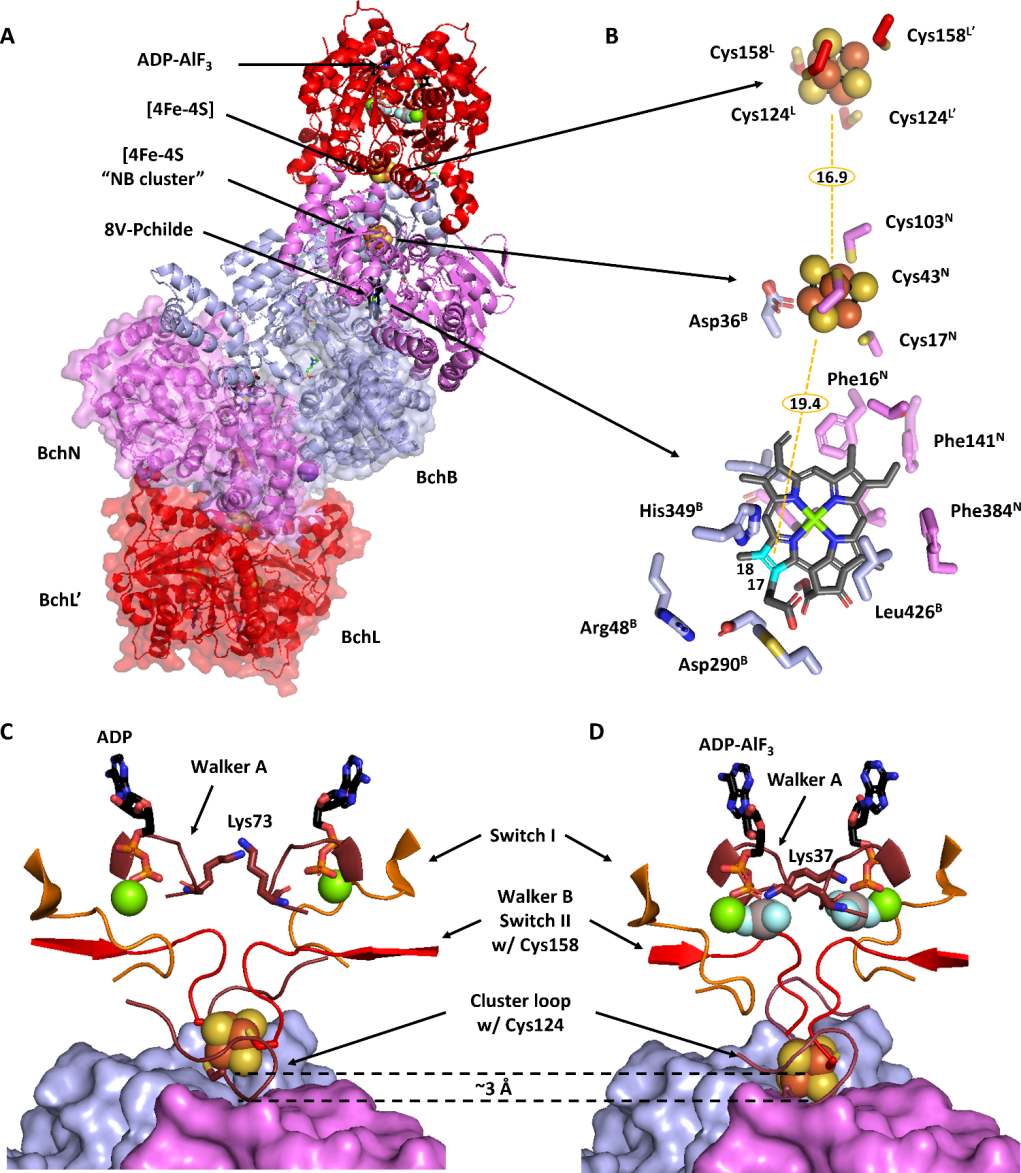
**Structure of DPOR BchL and BchNB under turnover (ADP-AlF**_**3**_ **bound) and nonturnover (ADP-bound) conditions.** (A) Overall DPOR structure and (B) spatial separation of the [4Fe-4S] clusters for electron transfer to the bound 8-vinyl-protochlorophyllide substrate. Intermolecular distances are given in Å. (C, D) Structure of DPOR (C) under ADP-bound non-turnover conditions and (D) under ADP-AlF_3_-bound “turnover” conditions. (C) and (D) show that the BchL Walker sites, Switch I/II regions, and cluster loop concertedly move the BchL [4Fe-4S] cluster toward the protein surface for electron transfer. PDB 3FWY and 2YNM. Superscripts designate the protein subunit, (BchL, BchN, or BchB).

Regarding electron transfer, a similar conformational change is observed in the DPOR Fe protein as seen in N_2_ase NifH upon ATP versus ADP nucleotide cofactor binding. Binding of ADP versus the ATP analog ADP-trifluoroaluminate to BchL causes the Switch I and Switch II domains to move the [4Fe-4S] cluster approximately 3 Å closer to the surface of the protein (Figure 24C,D).^63,104^ Based on these structural changes, it is proposed that BhcL and BchX will show a similar reduction in potential upon ATP binding to drive electron transfer to the catalytic components’ [4Fe-4S] cluster.^275,278^ Also, for BchL, specific structural elements appear to be autoregulatory for activity, which has previously not been observed in N_2_ase or other NFL systems. In the nucleotide-free state, acidic residues on the N-terminal region reposition to cover over the [4Fe-4S] cluster. Mutations to these residues lead to a BchL protein that is 2–3 times more active for electron transfer and turnover.^278^ Thus, the N-terminal tail may play an autoregulatory role for DPOR activity in response to available ATP levels.

The BchNB [4Fe-4S] cluster ultimately transfers an electron originating from the BchL [4Fe-4S] to the substrate. As introduced in section 2.3.2, the catalytic [4Fe-4S] “NB” cluster is asymmetrically coordinated by three cysteine residues from ChlN/BchN and a unique aspartate residue from ChlB/BchB (Figure 24B). In sequence alignments, the ChlB/BchB aspartate is in the same position as NifK Cys95 (Figure 10). Mutations to any of these coordinating residues results in 0–5% catalytic activity relative to the wild-type DPOR, implicating their role in proper metallocofactor coordination and electron transfer.^134^ In addition, DPOR also contains a cysteine residue in ChlB/BchB corresponding to NifK Cys153 in sequence alignments (*Av* numbering) (Figure 10). Prior to available atomic-resolution structures, the ChlB/BchB cysteine residue was previously proposed to coordinate the catalytic metallocofactor.^108,279^ Notably, mutational analysis revealed that the ChlB/BchB cysteine residue corresponding to NifK Cys153 was indispensable for activity, metallocofactor incorporation, and subunit association.^134^ Given available atomic resolution structures do not show the ChlB/BchB cysteine residue to coordinate the NB [4Fe-4S] cluster under dithionite reduced or turnover conditions,^134,276^ further work is required to determine if this cysteine residue coordinates a still-to-be-observed metallocofactor oxidation/reduction state during the catalytic cycle, or is alternatively involved in metallocofactor assembly. Likewise for COR, it is unknown whether BchYZ also uses an asymmetric coordination of its catalytic [4Fe-4S] cluster. In support of an asymmetric coordination, mutagenesis analysis of BchYZ from *Roseobacter denitrificans* along with activity assays, EPR, and metal analysis suggest that the same three BchY cysteine residues present in ChlN/BchN and the single BchZ cysteine residue in the same sequence position as the ChlB/BchB aspartate residue coordinate the catalytic [4Fe-4S] cluster (Figure 10, ? symbols).^280^

## 6 F430 Cofactor Synthesis: Group IV-B CfbCD

### 6.1 Biological Role and Synthesis of Cofactor F430

F430 is a tetrapyrrole-based cofactor (coenzyme) with a nickel prosthetic group found in methanogenic and methanotrophic archaea.^281–283^ F430 participates in the catalytic reaction of Methyl-CoM Reductase (MCR), which performs the last step of respiratory methane production by methanogens or the first step of anaerobic oxidation of methane (AOM) by anaerobic methanotrophic (ANME) archaea. AOM couples methane oxidation to reduction of Fe^3+^, humic acids, or metal oxides as terminal electron acceptors, or AOM syntrophically couples methane oxidation to sulfate reduction by sulfate reducing bacteria (SRB).^282,284,285^ In methanogenic archaea, MCR with F430 in the Ni­(I) reduced state binds methyl-coenzyme M (CH_3_–S–CoM; *S*-methyl-2-mercaptoethanesulfonate) followed by binding of reduced coenzyme B (HS-CoB; 7-mercaptoheptanoylthreoninephosphate). Subsequently, electron transfer between substrates results in release of methane and formation of the CoM–S–S–CoB heterodisulfide. For ANME archaea that perform AOM, the reverse reaction is catalyzed by MCR, where overall methane or a short chain alkane is added to CoM–S–S–CoB to produce HS-CoB and alkyl–S–CoM as the products.^282^ Due to the thermodynamics of this redox couple between methane and the heterodisulfide (Δ*G°* = −30 kJ/mol), the MCR oxidation reaction proceeds at 0.01% specific activity of the favorable CH_3_–S–CoM reduction reaction, which is sufficient to account for observed slow AOM activities by ANME archaea.^283^

F430 synthesis by archaea utilizes uroporphyrinogen III, the common precursor to tetrapyrrole-based cofactors, as introduced in section 2.3.4 ^286,287^ (Figure 25). First, the *S*-adenosyl-l-methionine-dependent uroporphyrinogen III methyltransferase (SUMT) of archaea (a.k.a. CobA or CysG in other organisms) adds a methyl group at C2 and C7. One SAM molecule and H^+^ is required for each methylation event, resulting in dihydrosirohydrochlorin (precorrin-2).^288–290^ The SUMT from archaea (27–28 kDa) is smaller than the bifunctional *E. coli* CysG enzyme (52 kDa), which contains an additional 22 kDa N-terminal domain that functions as an oxidase-ferrochelatase to convert precorrin-2 to siroheme.^291–293^ In contrast, methanogenic archaea possess a separate precorrin-2 dehydrogenase (PC2-DH) that oxidizes precorrin-2 by 2-electrons to sirohydrochlorin, analogous to the oxidation of precorrin-2 to sirohydrochlorin by SirC (*hemX*) in *Bacillus megaterium*.^289,290^

**Figure 25:**
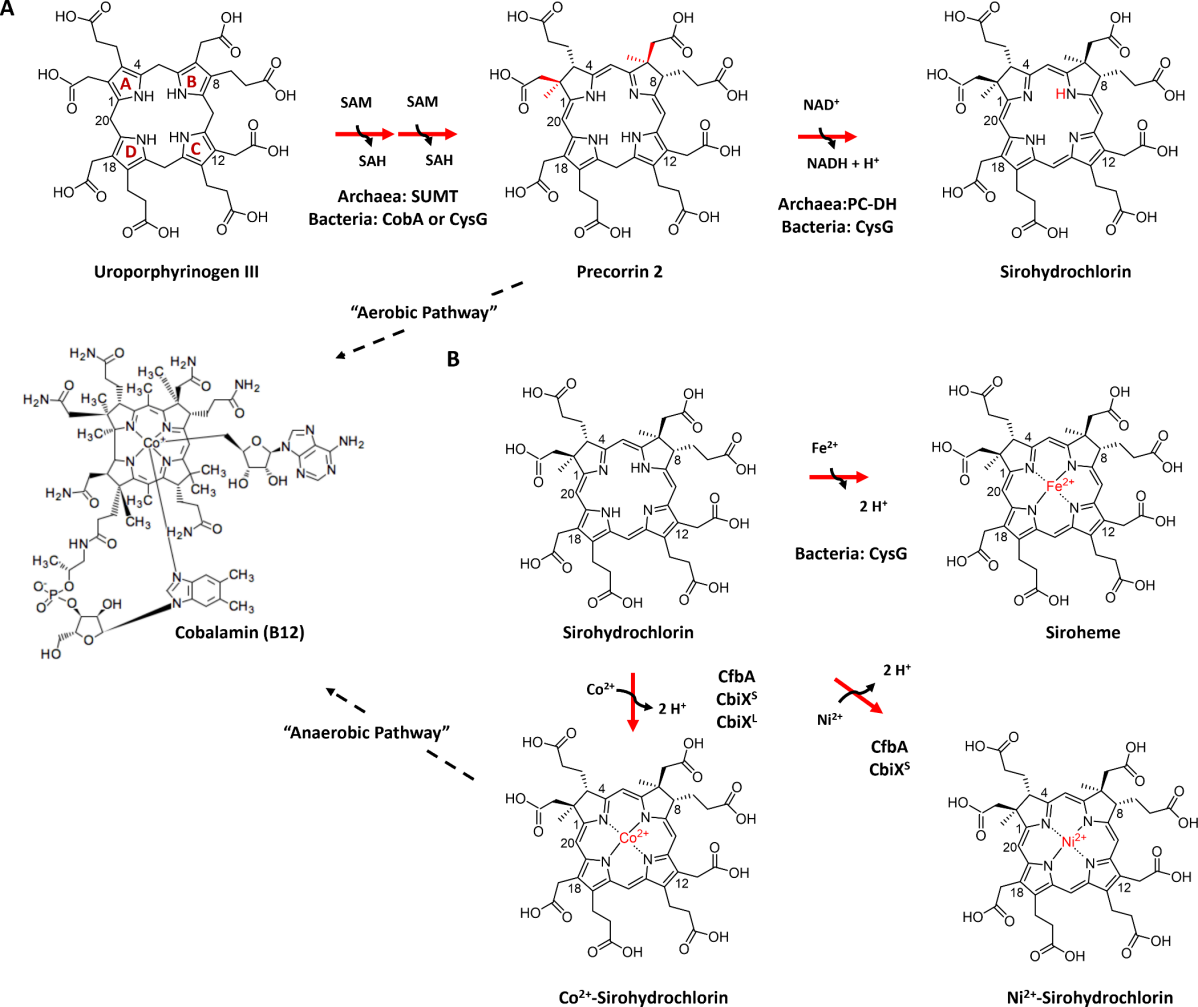
**Initial steps of F430 cofactor synthesis conserved in other bacteria and archaea for heme and cobalamin (B12) biosynthesis.** For (A) sirohydrochlorin synthesis and (B) siroheme (heme) synthesis, the bacteria CysG is a multifunctional protein. In archaea, *S*-adenosyl-l-methionine-dependent uroporphyrinogen III methyltransferase (SUMT) and precorrin-2 dehydrogenase (PC-DH) catalyze independent reactions.

Sirohydrochlorin is the branch point for the synthesis of siroheme (heme), cobalamin, and F430 cofactors in methanogenic archaea.^294–297^ A metal chelatase with specificity for both Ni^2+^ and Co^2+^ named CbiX^S^ (“conversion of precorrin-2 to adenosyl-cobinamide” proteins) or CfbA (“cofactor F430 biosynthesis” proteins) inserts Ni^2+^ into sirohydrochlorin to form Ni^2+^-sirohydrochlorin for F430 synthesis^117,133^ or inserts Co^2+^ to form Co^+^-sirohydrochlorin for vitamin B12 (cobalamin) synthesis^298^ (Figures 25 and 26). The “S” of CbiX^S^ designates “short” to differentiate the archaeal CbiX peptide sequences from the 2-fold longer bacterial cobaltochelatase homologues designated Cbix^L^, which are more specific for cobalt.^298^ Next Ni^2+^-sirohydrochlorin *a*,*c*-diamide synthetase, a homologue of CbiA cobyrinic acid *a*,*c*-diamide synthetase, transfers an amide group from two glutamine molecules to the *a*- and *c*-carboxylic acid moieties of Ni^2+^-sirohydrochlorin to form Ni^2+^-sirohydrochlorin *a*,*c*-diamide. In *Methanosarcina acetivorans* the diamide synthetase is designated as CfbB, and in *Methanosarcina barkeri* it is designated as CfbE.^117,133^

**Figure 26:**
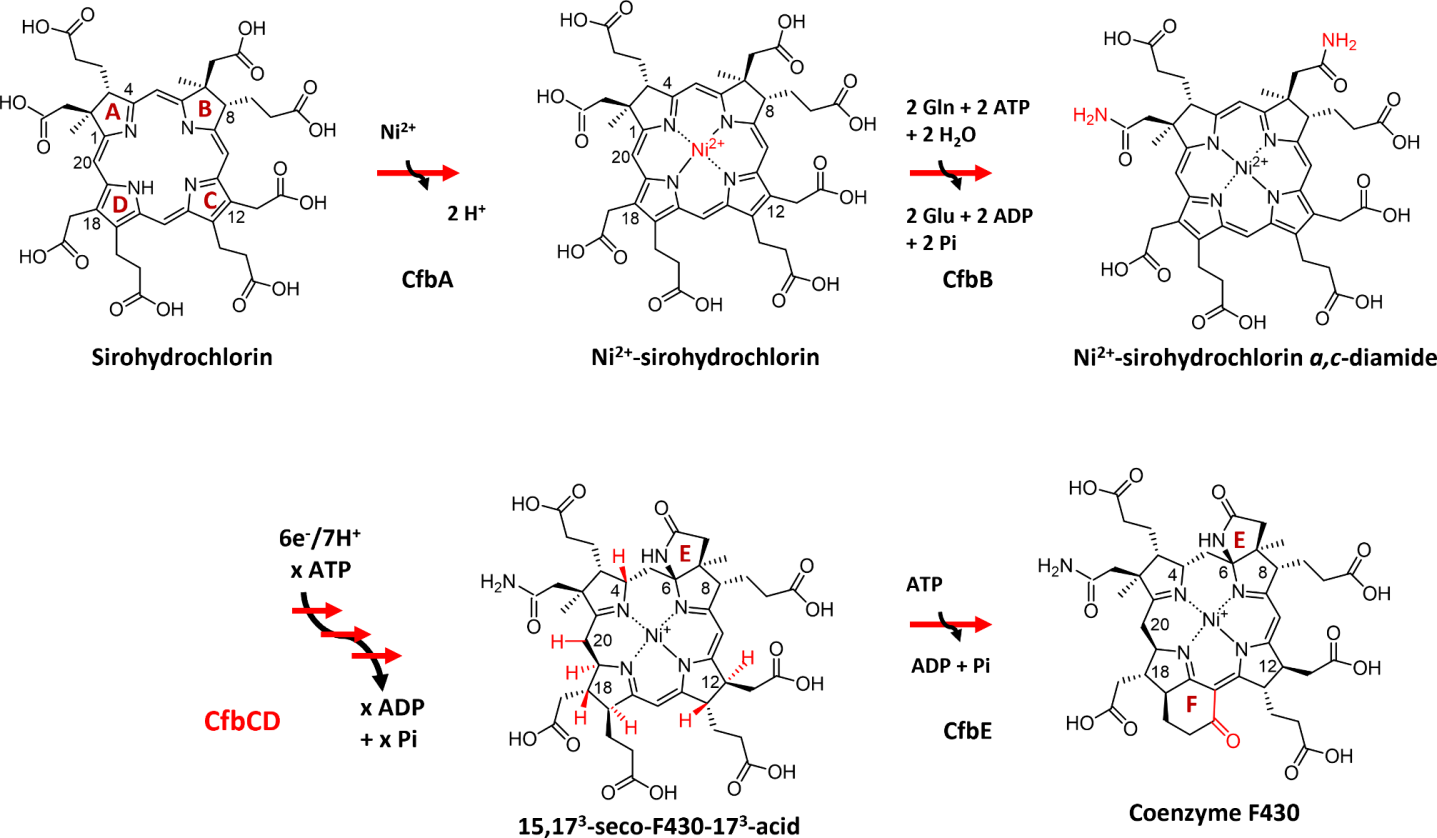
**Synthesis of coenzyme F430 in methanogenic and methanotrophic archaea.** CfbA, Ni^2+^/Co^2+^ chelatase; CfbB, cobyrinic acid *a*,*c*-diamide synthetase; NFL CfbCD, Ni^2+^-sirohydrochlorin *a*,*c*-diamide reductive cyclase; CfbE, F430 synthetase. Protein nomenclature is based on ref 117.

The quintessential 6-electron reduction of Ni^2+^-sirohydrochlorin *a*,*c*-diamide is performed by NFL CfbCD, a Ni^2+^-sirohydrochlorin *a*,*c*-diamide reductive cyclase that ultimately leads to formation of 15,17^3^-seco-F430-17^3^-acid as the penultimate species in the F430 biosynthetic pathway^299^ (Figures 6 and 26). For disambiguation, it is noteworthy that the NflH and NflD components of Ni^2+^-sirohydrochlorin *a*,*c*-diamide reductive cyclase are CfbC and CfbD, respectively, in *M. acetivorans*.^117^ But, they are designated as CfbD and CfbC, respectively, in *M. barkeri*.^133^ Henceforth, for the purposes of this review, the nomenclature of Zheng et al. of CfbC and CfbD as the NflH and NflD homologues, respectively, will be used for consistency with the N_2_ase field.^117^ In the ultimate step, F430 synthetase activates the *g*-propionate side chain of 15,17^3^-seco-F430-17^3^-acid in an ATP-dependent manner for cyclization and formation of the F430 carboxylic ring designated as “F”. In *M. acetivorans*, the F430 synthetase is designated as CfbE. Conversely, in *M. barkeri* the F430 synthetase is designated as CfbB.^117,133^ After completion of F430 synthesis, the cofactor is proposed to be shuttled to the MCR enzyme complex for insertion by chaperone McrD.^117^

### 6.2 Function and Structure of CfbCD

The Ni^2+^-sirohydrochlorin *a*,*c*-diamide reductive cyclase (CfbCD) is a two-component system of CfbC ATP-dependent reductase and catalytic CfbD. To date, representatives from *M. barkeri*, *M. acetivorans*, and *Methanocaldococcus jannaschii* have been partially characterized.^117,133,300^ The purified *M. barkeri* CfbCD *in vitro* initially performs a sequence of reduction reactions using six electrons and seven protons at three spatially distinct C=C double bonds across the Ni^2+^-sirohydrochlorin *a*,*c*-diamide macrocycle (Figure 26). Addition of six of the seven H^+^ to Ni^2+^-sirohydrochlorin *a*,*c*-diamide by CfbCD results in six new stereo centers in the macrocycle, which is proposed to be Ni^2+^-hexahydrosirohydrochlorin *a*,*c*-diamide based on primary mass, UV–vis absorption spectrum, and HPLC retention time compared to synthesized Ni^2+^-tetrahydrocorphinate.^133^ However, the order by which the reduction steps proceed and what dictates substrate specificity is currently unknown. For the *M. barkeri* CfbCD reaction assay, substrate reduction occurred within the first 90 min, followed by a slow, possibly nonenzymatic cyclization over 21 h of the *c*-acetamido group to from the γ-lactam ring E of the final 15,17^3^-seco-F430-17^3^-acid product. No reductase activity was observed in the absence of ATP or CfbC.^133^ In contrast, the *M. acetivorans* CfbCD enzyme system exhibited complete conversion to 15,17^3^-seco-F430-17^3^-acid in 7 h with no indication of an initial fast reduction phase and subsequent slower cyclization phase.^117^ Thus, at present it is unclear whether there are functional differences between CfbCD homologues from various archaea regarding catalysis of the γ-lactam ring E, if the ring formation is actually spontaneous with kinetic rates that are dependent on the reaction conditions, and if a yet to be identified cyclase is required for efficient ring formation to generate F430 *in vivo*. Regardless, due to the reductive function of CfbCD, the 15,17^3^-seco-F430-17^3^-acid product and ultimately F430 are the most reduced of all the known tetrapyrrole-based cofactors.

CfbC shares high predicted structural homology and sequence identity (52%) with NifH.^199,301,302^ The *Methanosarcina barkeri* CfbC Fe protein produced heterologously by *E. coli* and reconstituted with iron–sulfur clusters forms a homodimer that complexes a [4Fe-4S] cluster at the interface. Nonreconstituted CfbC from *E. coli* contains substoichiometric iron and sulfur content, exhibits low activity, and is primarily observed in the monomeric state as determined by size exclusion chromatography and iron/sulfide analysis.^133,302,303^ Mutational analysis of the CfbC cysteine residues Cys96 and Cys132 to alanine or serine (*M. barkeri* numbering) along with AlphaFold structural modeling indicated that these residues from each half of the CfbC homodimer coordinate the bridging [4Fe-4S] cluster at the homodimer interface,^302^ analogous to [4Fe-4S] coordination by the N_2_ase NifH cysteine residues Cys97 and Cys132 (*Av* numbering) (Figure 27). A [4Fe-4S] cluster with all-cysteine coordination by the CfbC Fe protein is supported by UV–vis absorbance spectroscopy with characteristic absorbance at ∼410 nm. Further, resonance Raman spectroscopy shows a predominant band at 336 cm^–1^ and further low-intensity bands around 350, 360, 380, and 390 cm^–1^ consistent with four thiolate ligands^20,76,302^ (Figure 27). X-band EPR spectroscopy of CfbC in the dithionite reduced state and no nucleotide cofactor exhibits features of [*g*_1_, *g*_2_, *g*_3_] = [2.06, 1.94, 1.87] and [*g*_1_, *g*_2_, *g*_3_] = [5.73, 5.01, 4.42] that arise from the *S* = 1/2 and *S* = 3/2 spin states of the [4Fe-4S]^1+^ cluster, respectively. Addition of ADP alters the relative intensities of these features, and addition of ATP alters the EPR lineshapes to [*g*_1_, *g*_2_, *g*_3_] = [2.03, 1.94, 1.87], similar to EPR spectra of NifH, VnfH, and AnfH in the absence versus presence of ATP.^16,133,304,305^ This observed behavior suggests that, analogous to the Fe proteins of N_2_ase, CfbC undergoes a conformational change upon binding of ATP for subsequent electron transfer to CfbD.^16^

**Figure 27:**
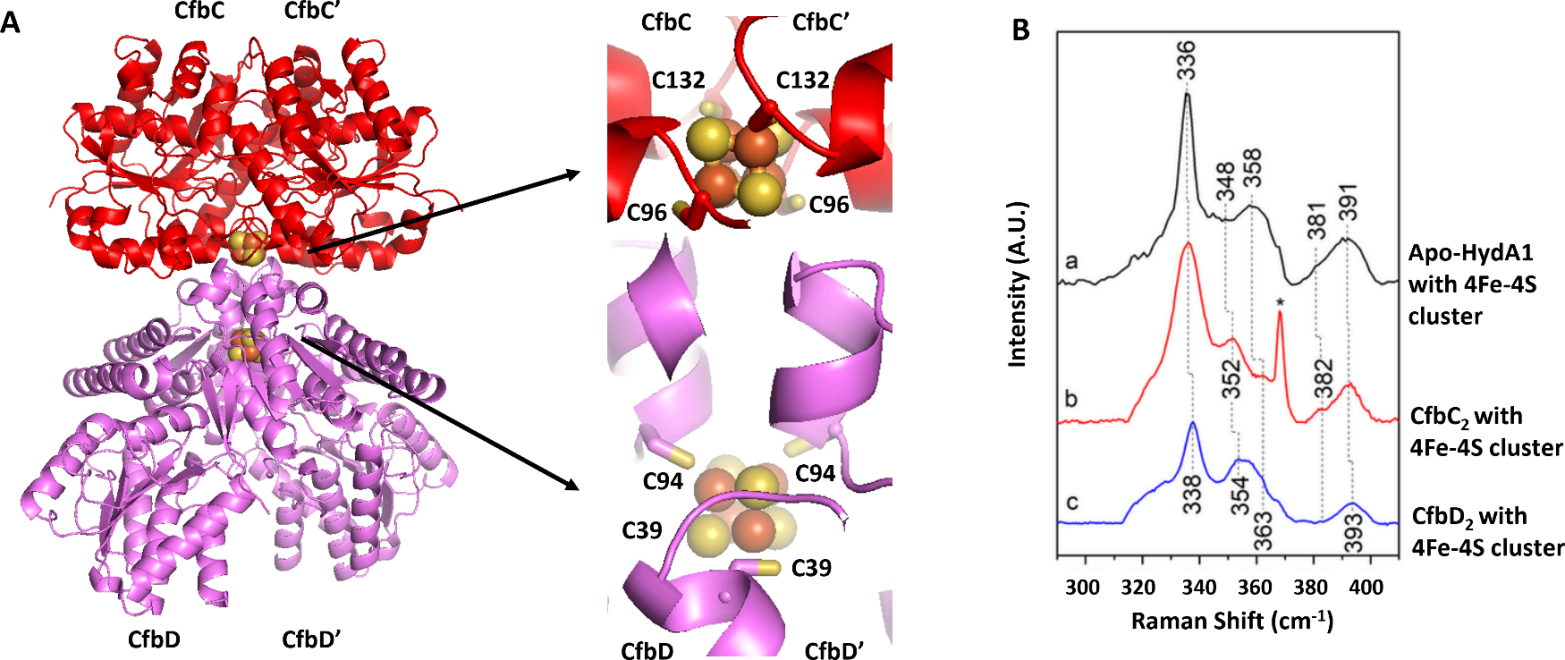
**Predicted structure and resonance Raman spectroscopy of CfbCD indicating an all-cysteine coordination of the CfbC and CfbD [4Fe-4S] clusters.** (A) AlphaFold 3 with Chai-I modeling of [4Fe-4S] cluster coordination, where each half of the CfbC and CfbD dimer provides two cysteine ligands for each metallocofactor based on ref 302. (B) Resonance Raman spectroscopy of the apo-FeFe hydrogenase (apo-HydA1) which contains a singular all-cysteine coordinated [4Fe-4S] cluster (PDB 6Gl6), and resonance Raman of CfbC and CfbD homodimeric proteins, also indicating an all-cysteine coordination. All spectra were normalized with respect to the nonresonantly excited phenylalanine side chain mode at ca. 1004 cm^–1^. The band at 368 cm^–1^ marked with an asterisk (*) is from an optical artifact. (B) reproduced from ref 302. CC BY-NC-ND 4.0.

A distinguishing feature of the catalytic CfbD subunit is its observed homomeric composition and symmetric catalytic metallocofactor coordination. CfbD residues Cys39 and Cys94 (*M. barkeri* numbering) correspond to NifD Cys88 and Cys154 in sequence alignments (*Av* numbering). Cys39 and Cys94 are observed to symmetrically coordinate the CfbD catalytic [4Fe-4S] cluster.^16^ Heterologous CfbD production in *E. coli* results in formation of a homodimer, irrespective of the presence of a metallocofactor.^133,303^ After reconstitution, subsequent iron/sulfide analysis, UV–vis spectroscopy, EPR spectroscopy, and AlphaFold modeling all indicate the presence of a [4Fe-4S] cluster^133,302^ (Figure 27). The X-band EPR spectrum of the dithionite reduced CfbD exhibits lineshapes for *S* = 1/2 and *S* = 3/2 signals corresponding to [*g*_*1=2*_, *g*_3_] = [2.05, 1.93] and [*g*_1_, *g*_2_, *g*_3_] = [5.73, 5.00, 4.15], respectively, irrespective of the presence of a bound nucleotide cofactor on the CfbC Fe protein. Raman resonance spectroscopy exhibited a predominant band at 336 cm^–1^ and additional low-intensity bands around 350, 360, 380, and 390 cm^–1^, consistent with an all-cysteine residue coordination of a [4Fe-4S] metallocofactor. Further, mutational analysis of the cysteine residues to serine or alanine and AlphaFold modeling indicate that the CfbD cysteine residues Cys39 and Cys94 (*M. barkeri* numbering) from each half of the CfbD homodimer coordinate the bridging [4Fe-4S] cluster at the homodimer interface.^302^ At present, high-resolution structures of CfbC and CfbD are yet to be determined, and thus the location of substrate binding for reduction is unknown.

### 6.3 Electron Transfer in CfbCD for Substrate Reduction

For the reduction of Ni^2+^-sirohydrochlorin *a*,*c*-diamide to Ni^2+^-hexahydrochlorin *a*,*c*-diamide, the midpoint potentials of reconstituted CfbC and CfbD proteins, measured separately and in the absence of nucleotide cofactors or substrate, were observed on average for CfbC to be *E*_m_ = −0.256 V and for CfbD to be *E*_m_ = −0.407 V relative to the standard hydrogen electrode (SHE). In comparison, the midpoint potential for *A. vinelandii* NifH is −0.30 V versus the SHE.^90,185,186^ When in the presence of nucleotides, the midpoint potential of NifH decreases to −0.43 V for ADP and −0.44 V for ATP versus the SHE.^185,186^ Similarly, the midpoint potential for NifH from *A. chroococcum* with ADP bound was observed to be −0.45 mV versus the normal hydrogen electrode (NHE).^187^ Additionally, complex formation between NifH and NifDK decreases the midpoint potential of NifH by −0.20 V to −0.62 V.^188^ A similar reduction in midpoint potentials for CfbC upon nucleotide binding and complexing with CfbD is anticipated.^302^ Thus, in the absence of nucleotide cofactors and complex formation, the thermodynamics of electron transfer from CfbC to CfbD is “uphill” and unfavorable. Such thermodynamic constraints suggest that analogous to N_2_ase, one electron is passed during each event when CfbC in the proper reduction and nucleotide cofactor state and is complexed with CfbD. Ultimately the sequential series of electron transfer events would result in the 6 electron reduction of Ni^2+^-sirohydrochlorin *a*,*c*-diamide. Notably, no partially reduced intermediates have been detected during CfbCD catalysis, suggesting that the Ni^2+^-hexahydrochlorin *a*,*c*-diamide or 15,17^3^-seco-F430-17^3^-acid product is not released from the CfbCD complex until it is fully reduced.^117,133,269^ However, the mechanistic sequence of reduction events on the Ni^2+^-sirohydrochlorin *a*,*c*-diamide substrate and the physiological electron donor, such as a ferredoxin or flavodoxin as observed for N_2_ase is unknown.^306–310^ Further, whether or not CfbC follows the same catalytic cycle of NifH with hydrolysis of two ATP per electron as discussed in section 3.1.3 is unknown.^191–195^ One striking observation is that when combined together, purified CfbC and CfbD from *M. barkeri* form a 1:1 complex with molecular weight of ∼135 000 Da, consistent with a tetrameric α_2_β_2_ assembly. Dissociation constants are estimated to be *K*_d_ < 15 μM, and are independent of the presence or absence of any nucleotide cofactor (ADP, ATP, or AMP-PNP).^311^ The observed stability of the CfbCD complex is in stark contrast to the transient complex between NifH and NifDK or between the DPOR and COR Fe proteins and catalytic components that must be trapped using nonhydrolyzable ATP analogs.^199,280,303,312^ Strong interactions have also been observed between the CfbC and CfbD of *Methanocaldococcus jannaschii* *in vivo* based on yeast two-hybrid genetic reporter systems and immunoprecipitation. Specifically, immunoprecipitation of CfbD by anti-CfbD cross-linked agarose from *M. jannaschii* cell lysate also recovered associated CfbC.^300^ Therefore, CfbCD may represent a stable catalytic complex with a mechanistic cycle for electron transfer that differs from N_2_ase and other known NFL systems.

### 6.4 Regulation of CfbCD

Little is known regarding the regulation of *cfb* genes in archaea. While *M. barkeri* and *M. acetivorans* organize *cbiX* ^*S*^ and *cfbBCDE* as gene clusters on the chromosome, *M. jannaschii* *cfb* gene homologues are dispersed throughout the chromosome.^313^ In *M. jannaschii*, protein abundance of CfbC and CfbD was quantified by Western immunoblot analysis as a function of growth conditions.^300^ Irrespective of ammonium concentration used as the nitrogen source, and irrespective of the inclusion of low concentrations of compounds inhibitory to growth (sodium azide, isothiocyanate, cyanide), the same abundance of CfbCD was detected. The consistent CfbCD abundances suggests that the expression of *cfbC and cfbD* is constitutive, consistent with the role CfbCD in cofactor biosynthesis. However, further work with other methanogens to determine if constitutive *cfb* gene expression is a common paradigm for F430 biosynthesis, or if conditions relevant to F430 biosynthesis such as during Ni^2+^ limitation and metabolic shifts between hydrogenotrophic, methylotrophic, and acetoclastic (acetotrophic) growth reveal regulation.

## 7 Unknown Cofactor Synthesis: Group VI NFL Systems

The Elusimicrobia family of bacteria possess a diverse group of NFL sequence designated as Group VI, which are distinct from the Group IV-A Nfa. Group VI sequences are proposed to be involved in tetrapyrrole-based cofactor synthesis based on genome context.^121^ The Group VI NflH sequences share ∼40% sequence identity with NifH, and they possesses two cysteine residues corresponding to NifH Cys97 and Cys132 in sequence alignments (*Av* numbering) for likely [4Fe-4S] cluster binding. Group VI NflH sequences also possess the Walker A, Walker B, Switch I, and Switch II motifs for ATP binding, hydrolysis, and signal transduction between the nucleotide and the [4Fe-4S] cluster.^63,102,104,121^ Notably, no NifB homologues are observed in the genomes of organisms only possessing Group VI NFL sequences. Further, in analogy to DPOR, COR, and CfbCD, the key conserved NifD Cys275 and His442 residues (*Av* numbering) used by N_2_ase for FeMo-co coordination are missing in the Group VI NflD and NflK sequences.^102,121^ The absence of NifB and FeMo-co coordination ligands suggests that the Group VI NFL systems function using simpler iron–sulfur clusters. Notably, the Group VI NflD and NflK sequences are highly divergent, sharing only 22.5% and 22.7% identity on average with NifD and NifK, respectively. In approximately 1/3 of Elusimicrobia genomes with Group VI systems, only NflH and NflD were present, analogous to CfbCD. In the remaining genomes, NflH, NflD and NflK were all present. Notably, cysteine residues at analogous positions used by N_2_ase for P-cluster coordination, and DPOR, COR, and CfbD for catalytic [4Fe-4S] cluster coordination where highly variable across Group IV NFL sequences.^121^ Further, for each Group VI NflDK set of sequences, at most four cysteine residues were observable among the set of six conserved residues used by N_2_ase for P-cluster binding, further supporting the conclusion that Group VI NFL systems likely use [4Fe-4S] or similar clusters for catalysis.^121^ Ultimately, there is much to learn about these elusive NFL systems regarding the processes they catalyze and metallocofactors employed.

## 8 Volatile Organic Sulfur Compound Cleavage: Group IV-C MAR

### 8.1 Dimethylsulfide and Methylthioethanol in Nature

Volatile organic sulfur compounds (VOSCs) are ubiquitous in the environment, spanning atmospheric,^314,315^ marine,^316,317^ freshwater,^318–320^ and terrestrial ecosystems.^321,322^ In addition, numerous VOSCs are also present in crude petroleum and fossil-fuel hydrocarbon deposits.^323^ VOSCs are produced by animals, plants, fungi, and bacteria via a diverse number of pathways that typically involve utilization or synthesis of the amino acids methionine and cysteine as part of energy, carbon, and/or sulfur metabolism.^118,324–327^ Currently, environmental VOSCs known to be specifically relevant to methylthio-alkane reductase (MAR) are dimethyl sulfide (DMS) and 2-methylthioethanol (MT-EtOH).^102^ Whether other VOSCs serve as substrates for MAR cleavage remains unknown.

DMS is the highest-abundance VOSC on the planet. Approximately 20–33 Tg of sulfur in the form of DMS is produced each year by bacterial processes in the ocean.^328,329^ Oceanic DMS represents 85% of the global DMS production, with terrestrial vegetation processes contributing an additional 13% and soil microbes the final 1–2%.^329^ Specifically, marine algae, corals, plants, and heterotrophic bacteria, including deep sea species, first produce more than 2 petagrams of organic sulfur in the form of dimethylsulfoniopropionate (DMSP) each year.^330–332^ In these organisms, DMSP is synthesized from methionine and is proposed to act as an osmolyte, hydrostatic pressure protectant, cryoprotectant, predator deterrent, and/or antioxidant.^332–335^ DMSP released into the ocean environment is then utilized by marine bacteria as a carbon and energy source through multiple distinct pathways that produce methanethiol (demethylation pathway), DMS (cleavage pathways), or dimethyl sulfoxide (DMSO) (oxidation pathway) as byproducts (reviewed in ref 336). For terrestrial plants, DMS production varies by species, and likely arises from enzymes that cleave *S*-methyl-l-methionine, DMSP, or dimethylsulfoniopropionaldehyde synthesized by the organism.^337,338^ Lastly in soils, known pathways of DMS production are widespread in bacteria.^321,322^ Herein, methionine is first cleaved to methanethiol by methionine gamma lyase (MGL), followed by SAM-dependent methylation of methanethiol to DMS by MddA (for MeSH-dependent DMS production protein). Alternatively, MddA can also directly methylate hydrogen sulfide (HS^–^) to methanethiol, followed by a second methylation to DMS.^339^ Furthermore, a separate SAM-dependent HS^–^ and methanethiol methyltransferase that produces DMS is designated as MddH and has been identified in soil, freshwater, coastal, and marine bacteria.^340^

Annual global biological production of MT-EtOH is not known, but MT-EtOH has been observed to be synthesized by plants (melon, tomato, beans^341–343^ ), yeasts,^344,345^ and bacteria (*Lactococcus* sp., *Rhodospirillum rubrum*, *Rhodopseudomonas palustris*, *E. coli* sp.) via pathways that are presumed or verified to use methionine.^118,325–327,346,347^ The best characterized pathway for MT-EtOH synthesis is the dihydroxyacetone phosphate shunt (DHAP), which is found in 10% of sequenced bacteria, predominantly in freshwater, soil, and pathogenic species.^118,327^ All extant organisms synthesize SAM from methionine or acquire it from the environment for methyltransferase reactions, radical SAM reactions, and synthesis of polyamines, acyl- and aryl-homoserine lactone quorum sensing compounds, modified lipids, and natural products (reviewed in refs 348 and 349). SAM can function as an α-aminobutyryl donor, or SAM can be decarboxylated by SAM decarboxylase to form *S*-adenosyl-(5′)-3-methylthiopropylamine (a.k.a. dcSAM) as a γ-aminopropyl donor. When SAM is used for synthesis of quorum sensing compounds, modified lipids, and natural products, then 5′-methylthioadenosine (MTA) is released as a byproduct.^350,351^ Enzymes of the DHAP shunt further metabolize MTA to DHAP and 2-methylthioacetaldehyde. Two variations of the DHAP shunt have been characterized, which begin either with the bifunctional MTA phosphorylase (MtnP, a.k.a. MTAP) to form adenine and 5-methylthioribose-1-phosphate, or begin with the monofunctional MTA nucleosidase (MtnN, a.k.a. Pfs) to form adenine and 5-methylthioribose (Figure 28). In the case of the ladder, 5-methylthioribose kinase (MtnK) converts 5-methylthiorobise to 5-methylthioribose-1-phosphate. Subsequently, 5-methylthioribose-1-phosphate isomerase (MtnA) forms 5-methylthioribulose-1-phosphate as the substrate for the class II 5-methylthioribulose-1-phosphate aldolase (Ald2), resulting in DHAP and 2-methylthioacetaldehyde.^118,326,327,346,352^ While the precise enzyme is unknown, presumably an alcohol dehydrogenase homologue performs the final reduction of 2-methylthioacetaldehyde to MT-EtOH for MAR. Notably, 5′-deoxyadenosine produced as a byproduct from radical SAM reactions also serves as a substrate for the DHAP shunt with nearly equal catalytic efficiency as MTA to produce DHAP and acetaldehyde.^118,352^ Further, the physiological role of the DHAP shunt is organism specific:

(A) *E. coli* employs the DHAP shunt to salvage DHAP from MTA and 5dAdo as a carbon and energy source for growth.^118,346,353^

(B) *B. thuringiensis* employs the DHAP shunt to prevent inhibitory 5dAdo and 5-deoxyribose accumulation from radical SAM reactions.^352^
(C) Nonsulfur purple alphaproteobacteria like *R. rubrum*, *R. palustris*, and *Blastochloris viridis* employ the DHAP shunt to intracellularly produce MT-EtOH as a substrate for MAR.^102,118,326,327^

**Figure 28:**
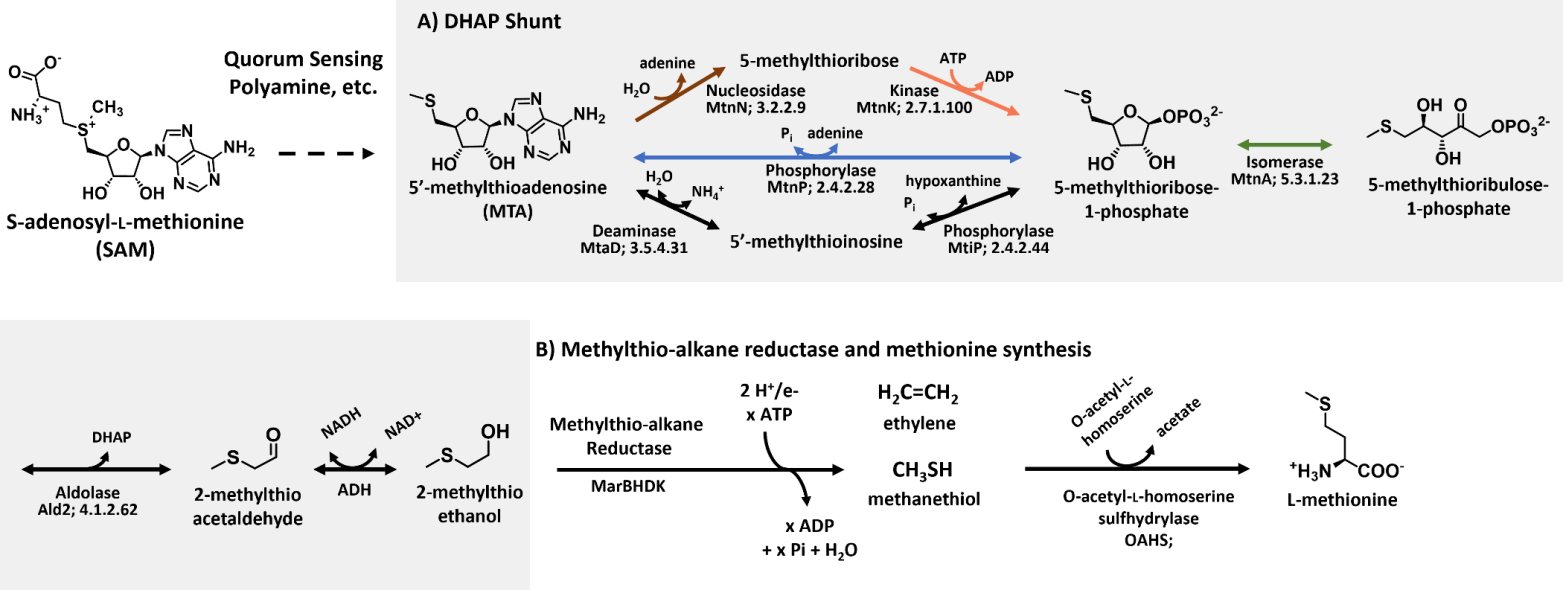
**Role of MAR in sulfur salvage from 5′-methythioadenosine (MTA) nucleotide.** (A) Dihydroxyacetone shunt pathway for 5′-methylthioadenosine (MTA) metabolism to 2-methylthioethanol (MT-EtOH) and (B) conversion into methionine of methanethiol produced from volatile organic sulfur compounds like DMS and MT-EtOH by MAR.

When MAR cleaves DMS and MT-EtOH for sulfur salvage, the resulting methanethiol is coupled with *O*-acetyl-l-homoserine or *O*-succinyl-l-homoserine by *O*-acetyl-l-homoserine sulfhydrylase or *O*-succinyl-l-homoserine sulfhydrylase, respectively, to regenerate needed methionine for the cell^102,120,327^ (Figure 28). While the contribution of DMS and MT-EtOH cleavage by MAR to form methanethiol and ultimately methionine for cell growth in the environment is unknown, it is proposed to function in both intracellular and intercellular sulfur salvage. The sulfur salvage model is based on the observation that available sulfate in freshwater and soil environments can spatially and temporally range from <1 μM to 3 mM, and for many organisms cell growth is limited when total available sulfur becomes ≤100 μM (see ref 327 and references therein).

### 8.2 Function and Structure of MAR

#### 8.2.1 Identification of MAR Function

The function of MAR was initially identified in the purple nonsulfur bacteria, *Rhodospirillum rubrum* (*Rr*), *Rhodopseudomonas palustris*, and *Blastochloris viridis*.^102^ MAR activity stoichiometrically cleaved MT-EtOH to ethylene, cleaved DMS to methane, and similarly cleaved ethylmethylsulfide (EMS) to ethane^102,327^ (Figure 7). Synthesis of (2-[C^14^-methyl]­thio)­ethanol and subsequent pulse-chase feeding of *Rr* with and without MAR genes revealed MAR activity resulted in direct production of methanethiol followed by formation of methionine for cell metabolism (Figure 28). Free methanethiol release refuted previous presumptions that VOSC cleavage by MAR to from the methanethiol product required a thiol-carrying cofactor like glutathione,^326,327^ in contrast to cleavage of 1-methylthio-d-xylulose-5-phosphate by 1-methylthio-d-xylulose-5-phosphate methylsulfurylase, which results in an *S-*methylthio-glutathione intermediate.^119,120,354^ Beyond proteobacteria, phylogenetic analysis indicates that functional MAR systems are also likely present in *Clostridia* sp., *Fibrobacter*, and Negativicutes. In the absence of *marH*, *marD*, or *marK* genes from the chromosome, no MAR activity is observed in *Rr*, indicating that the other N_2_ase Nif and Anf systems and NFL DPOR, COR, and NflDK systems of *Rr* cannot compensate for or cross-react with MAR.^100^ Activity was restored upon reintroduction of these missing genes via expression from a plasmid, establishing their specific involvement in MAR function. Located on the *Rr* genome near the *marBHDK* genes is an additional set of *nflDK* genes.^102,123,124^ These genes were completely dispensable for MAR activity *in vivo*. Similarly, the N_2_ase metallocofactor assembly scaffold, NifEN, was also dispensable for MAR activity, indicating that MAR metallocofactor assembly occurs *in situ* analogous to the FeFe N_2_ase.^100^

Recently, MAR function has been confirmed using purified proteins homologously produced in *Rr* and heterologously produced in *R. capsulatus*.^100,101^ The isolated catalytic MAR system is composed of a homodimeric MarH Fe protein and a heterotetrameric MarDK component. Activity requires both components, ATP, an electron donor (dithionite), and oxygen-free conditions to cleave DMS, EMS, and MT-EtOH into methanethiol and the respective hydrocarbon.^100,101^ Notably, while purified MAR can also reduce acetylene to a mixture of ethylene and ethane as observed in the alternative N_2_ases, it does not appear to be able to reduce N_2_ to ammonia, establishing MAR and N_2_ases as functionally distinct systems. MAR also can reduce protons to produce H_2_, but whether H_2_ is an obligate part of the MAR catalytic cycle as for N_2_ase is unknown.^101^

#### 8.2.2 Structure of MarH and MarDK

MarH shares 47.3% sequence identity with NifH, and possesses cysteine residues Cys97 and Cys133 (*Rr* numbering) in the same sequence alignment position as NifH Cys97 and Cys132 (*Av* numbering) for [4Fe-4S] cluster binding. Metal analysis (ICP-MS) and cryo-EM structural determination of MarH complexed with MarDK by MgADP–AlF_3_ shows that MarH is a structural homologue of NifH with a [4Fe-4S] cluster coordinated at the dimeric interface by Cys97 and Cys133 from each of the two monomers. Further, MarH possesses the Walker A, Walker B, Switch I and Switch II motifs for ATP binding, hydrolysis and signal transduction between bound nucleotides and the [4Fe-4S] cluster^63,101,102,104^ (Figure 29). Currently, whether or not MarH follows the same catalytic cycle of NifH with hydrolysis of two ATP per electron as discussed in section 3.1.3 is unknown.^191–195^ Further, the physiological electron donor for MAR catalysis in *Rr* is unknown, although a ferredoxin is implicated.^102^

**Figure 29:**
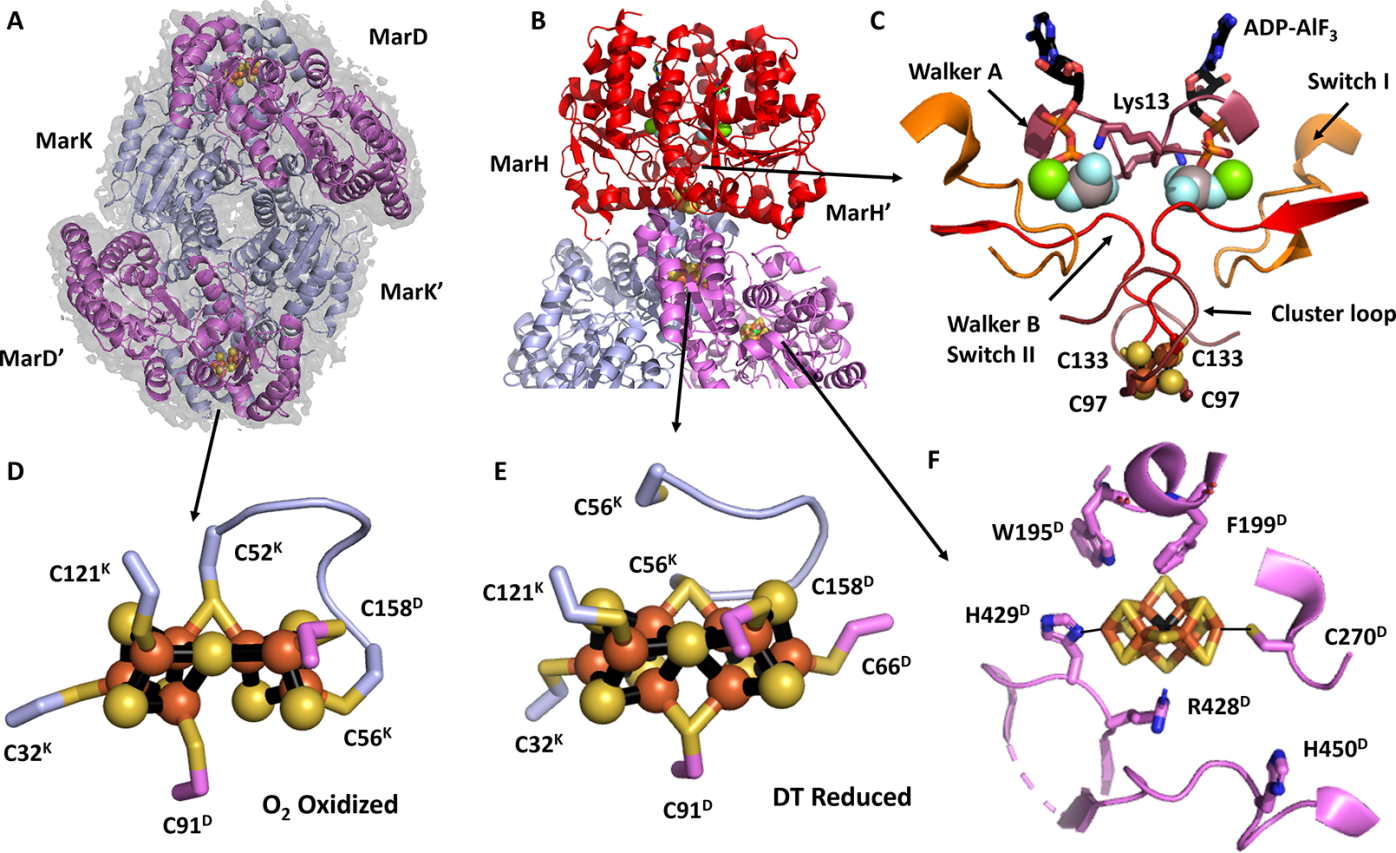
**Structure of methylthio-alkane reductase of *Rhodospirillum rubrum*.** (A) MarDK tetramer under O_2_ oxidizing conditions (PDB 9D9U)^100^ and (B) MarH dimer complexed with MarDK tetramer with ADP-trifluoroaluminate under dithionite reducing conditions (PDB 9FMG).^101^ (C) MarH nucleotide and [4Fe-4S] coordination by Walker A/B, Switch I/II, and cluster loop regions. Residue numbers are for *R. rubrum* MAR. (D, E) MarDK P-cluster under O_2_-oxidized (mar1-cluster) and dithionite-reduced conditions, respectively. (F) MarDK catalytic mar2-cluster metallocofactor, which appears to be an L-cluster, but the central carbide is unverified. Superscripts D and K correspond to MarD and MarK proteins, respectively.

Structures of MarDK in the oxygen oxidized state and MarDK complexed with MarH by MgADP–AlF_3_ in the dithionite reduced state both show that MarDK is based on the N_2_ase fold. Each MarD and MarK peptides assemble into three subdomains composed of four or five β-sheets surrounded by five α-helices (Figure 29). MarDK neither possesses the extended NifK N-terminal domain of MoFe N_2_ase nor the extended AnfD C-terminal domain for FeFe N_2_ase. Moreover, MarDK does not possess the δ subunit (VnfG or AnfG) of VFe and FeFe alternative N_2_ases, making MarDK closest in resemblance to VFe N_2_ase without a δ subunit.^100,101^ Both MAR structures indicate the presence of a [8Fe-7S] P-cluster at each of the two MarDK interfaces and the dithionite-reduced structure also indicates an 8Fe-containing L-like cluster in the NifD subunit as the likely catalytic site. The presence of a P-cluster and L-cluster are corroborated by ICP-MS quantification of ∼32 Fe per MarDK tetramer and negligible V or Mo.^100,101^ Currently, the L-like cluster is designated as the mar2-cluster, as the presence of a carbide in the center of the cluster cannot be unambiguously assigned in cryo-EM analyses (Figure 29). In the oxygen-oxidized structure, the L-like mar2-cluster is absent, reflective of the high oxygen sensitivity of the enzyme.^100,101^ The MAR metallocofactor intramolecular distances of 15.0 Å between the MarH [4Fe-4S] and MarDK P-cluster and 14.4 Å between the P-cluster and mar2-cluster are very similar to N_2_ase at 15.3 Å between the NifH [4Fe-4S] cluster and the NifD_K_ P-cluster and 14.9 Å between the P-cluster and FeMo-co^101^ (Figures 14 and 29). Altogether these structural studies indicate that MarH bound to MarDK is poised to perform electron transfer for catalysis in a manner similar to N_2_ase, but with some salient difference as detailed below.

In the dithionite reduced state, the P-cluster is coordinated by MarD Cys66, Cys91, Cys158 and MarK Cys32, Cys56, Cys121 (*Rr* numbering).^101^ These are the same conserved cysteines present in all known N_2_ases for P-cluster binding (Figure 10), and for the first time reveals an NFL system that coordinates a P-cluster in the same way as N_2_ase in the reduced P^N^ state, confirming previous postulations based on amino acid conservation^102^ (Figure 29). In the oxidized structure, a P-like cluster was also observed, but with a different arrangement of coordinating cysteine residues. MarK possesses an additional cysteine residue, MarK Cys52, that is located near P-cluster coordinating residue MarK Cys 56. While MarK Cys52 was not observed to interact with the P-cluster in the dithionite reduced structure, it replaced MarK Cys56 in coordinating belt irons Fe4 and Fe5 in the oxidized state. Further, MarK Cys56 shifted to coordinate iron Fe7 in lieu of MarD Cys66 (NifD Cys62 in *A. vinelandii*), which made no contact and instead was oriented away from the cluster.^100^ Lastly in the oxygen oxidized state, the P-like cluster exhibited oxidative loss of one of the belt irons, similar to that observed in IDS oxidized N_2_ase mutant NifK S188A^355,356^ (Figure 29). The MoFe N_2_ase NifK residues Ser188 or Tyr98, which coordinate the P^2+^ oxidized state and provide some oxygen protection, are absent in MAR.^355,357^ Thus, while the oxidation state and catalytic relevance of the P-cluster coordination in the oxygen oxidized structure is unknown, the differences between the dithionite reduced and oxygen oxidized structures suggest that MAR may coordinate oxidized and reduced P-cluster states for electron transfer in a different way from N_2_ase.

For substrate reduction, the presumed catalytic L-like mar2-cluster coordination environment differs from that of N_2_ase. No homocitrate and no histidine residue in the same sequence position as NifD H442 (*Av* numbering) are present. Thus, there is no apparent tridentate coordination of the terminal mar2-cluster Fe ion in contrast to N_2_ase coordination of the Fe, V, or Mo heterometal in the FeMo-co, FeV-co, and FeFe-co, respectively (Figures 16 and 29). In contrast, a different MarD histidine residue coordinates one of the mar2-cluster terminal Fe ions. This His429 residue (*Rr* numbering) is conserved across many other Group IV NFL sequences (Figure 11). On the opposite end of the mar2-cluster, the Cys270 residue coordinates the other terminal Fe ion. Notably, Cys270 corresponds to NifD Cys275 in sequence alignments (*Av* numbering) for FeMo-co coordination, and is also conserved across other Group IV NFL sequences (Figure 11). Further, MarD Arg428 is located near belt sulfur S5A along the mar2-cluster Fe1–Fe3–Fe7–Fe8 edge (Figure 29). Notably in MAR, NifD Arg96 and Arg359 (*Av* numbering) are absent, which in MoFe N_2_ase are positioned along the Fe1–Fe3–Fe7–Mo edge. NifD Arg96 and Arg359 have been proposed to help stabilize the charge distribution that accumulates on the Fe2–Fe3–Fe6–Fe7 face of FeMo-co during catalysis.^358^ One possibility is that the MarD Arg428 performs a similar function. Alternatively, based on computational substrate channel prediction, the MarD Arg428 may be involved in correctly positioning belt sulfur S5A such that the substrate channel is structured to pass by this sulfur and approach sulfur S2B to access the presumed catalytic face of the metallocofactor.^101^ Mutational analysis in which the mar2-cluster coordinating MarD Cys270 is substituted to an alanine or His429 is substituted to a leucine or phenylalanine results in an inactive enzyme for VOSC cleavage. Conversely, substitution of MarD Cys73, which in N_2_ase is the gatekeeper Val70 (*Av* numbering), and mutation of MarD His450, which is on the same loop as N_2_ase His442 (*Av* numbering) for FeMo-co coordination, has no effect on MAR activity. These mutational studies corroborate the structural models that MarD Cys270 and MarD His429 are requisite residues for metallocluster binding.^100^ Notably, MarD Cys270, His429 and Arg428 are conserved across nearly all Group IV NFL systems, indicating a common motif is utilized for metallocofactor binding and/or catalysis (Figures 11 and 29).

In addition to differences in metallocofactor coordination between MAR and N_2_ase systems, the potential environment for substrate coordination also differs. Helix 9 of N_2_ase above FeMo-co belt sulfur S2B is NifD 190-SQSLGHH-196 (*Av* numbering), whereas in MAR it is MarD 194-HWSTGFD-200 (*Rr* numbering) (Figures 16 and 29). In N_2_ase, Gln191 and His195 are required for N_2_ reduction,^359^ in NifD Gln191 also coordinates O1 of the *R*-homocitrate. As thoroughly reviewed elsewhere,^7^ conserved Gln191 and His195 residues in Nif and Vnf N_2_ases are involved in coordinating the belt sulfur S2B both when bound to FeMo-co and FeV-co or displaced by substrate binding via a hydrogen-bonding network. Structural studies and computational modeling indicate this hydrogen-bonding network may extend to the protein surface and likely provides protons for substrate and metallocofactor reduction intermediates.^212,360–362^ In the cryo-EM structure of MAR under dithionite reducing conditions, MarD His194, Trp195, and His375 are each within hydrogen-bonding distance to sulfur S2B.^101^ Substitution of MarD His194 to an alanine or phenylalanine had negligible effect on substrate reduction activity.^100^ Similarly, substitutions to Phe199 and D200, which are more distal to S2B had no effect. Only the substitution of Trp195 with an alanine resulted in an inactive enzyme, implicating a possible role in substrate or metallocofactor belt sulfur coordination. However, further studies are required to determine how VOSC substrates are coordinated for catalysis, which metallocofactor iron(s) function in reductive catalysis, and whether the belt sulfurs are dynamically displaced by substrate binding.

### 8.3 Function of MarB and NifB in Metallocofactor Maturation

MarB shares 40.9% sequence identity with NifB from *A. vinelandii* and possesses all of the identified motifs conserved across NifB enzymes for coupling together two [4Fe-4S] clusters and inserting a central carbide in a SAM-dependent manner.^18,102^ Genetic studies of *Rr* implicate that MarB and NifB share common functionalities. When both MarB and NifB are present in the organism, or when only MarB is present in the organism, *in vivo* MAR activities are at wild-type levels (50–100 μmol of DMS or MT-EtOH cleaved per liter of culture per unit culture density over 72 h). Conversely, when only NifB is present in the organism, MAR is functional but with an altered activity profile; MT-EtOH reduction to ethylene increases 2-fold while DMS reduction to methane decreases >2-fold. In the absence of both NifB and MarB, no activity is detectable.^100^ Thus, MarB and NifB each must be able to synthesize and insert a functional metallocofactor into MarDK. However, whether the metallocofactors produced by MarB versus NifB differ in identity, orientation of insertion into MarDK, or both are unknown. Regardless, given the association of NifB homologues with Group IV NFL systems like MAR and of unknown function, these observations point to commonalities in complex metallocofactor identity and underlying catalytic mechanisms for substrate reduction across the Group IV NFL systems.

### 8.4 EPR Spectroscopy of MAR

EPR spectra of the metallocofactors of MarH and MarDK resemble, to varying degrees, prior results on N_2_ases, implicating further structural and mechanistic parallels between the systems. Like NifH, isolated MarH shows two distinct sets of signals for an *S* = 1/2 and an *S* = 3/2 [4Fe-4S]^+^ species, with only ∼20% in the *S* = 1/2 spin state (Figure 30A).^305,363^ Slight changes in the line shape and spin state populations are observed upon addition of ATP, indicative of nucleotide binding to the protein and suggesting a conformational change. These observations are very similar to prior observations of NifH. The MarDK complex in the absence of MarH shows distinct signals upon oxidation that are reminiscent of the N_2_ase L-cluster (a.k.a. NifB-co), either when assembled on the NifEN scaffold or extracted into solution.^101^ While the electronic origins of these unusual features are poorly understood, the low g-values (*g*_avg_ ≈ 1.91) and asymmetric line shape observed are considered to be the hallmark of a low-coordinate [8Fe-S-9C] cluster and support the structural data assignment of the active cofactor to a carbide-bearing L-like cluster (Figure 30C).^145,211,364^

**Figure 30:**
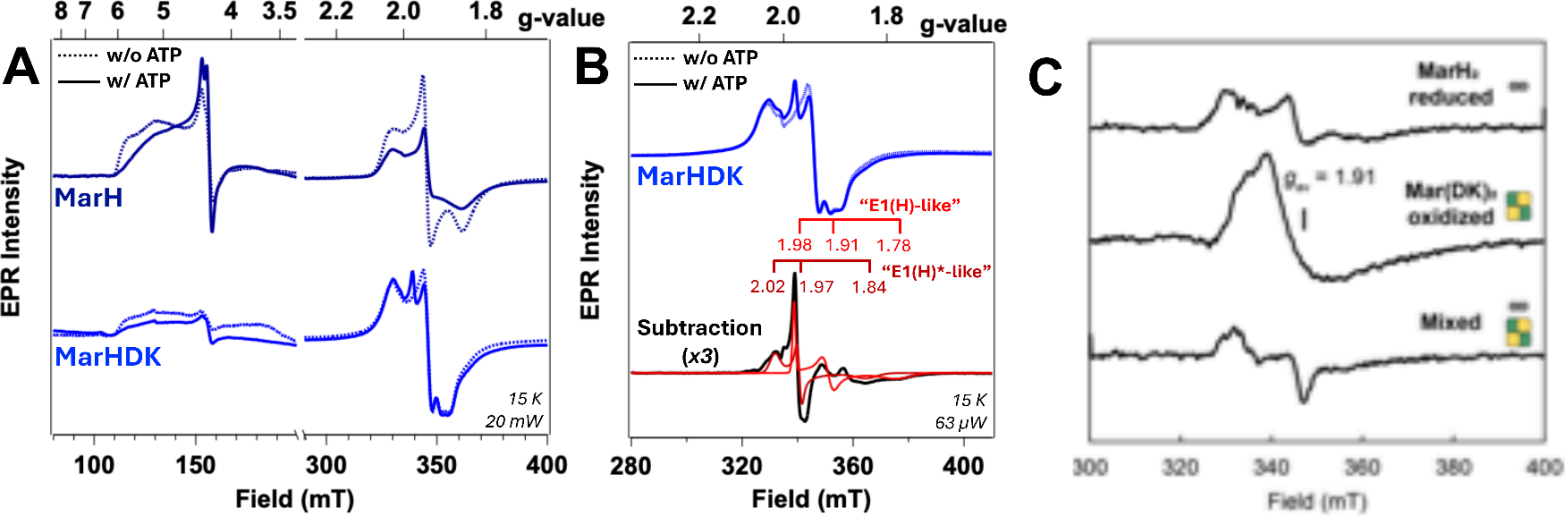
**EPR spectroscopy of methylthio-alkane reductase.** (A) EPR spectroscopy on MarH and MarH + MarDK in the presence (solid traces) and absence (dotted lines) of ATP. The spectrum shows both *S* = 3/2 and *S* = 1/2 species at *g* ≈ 5 and *g* ≈ 2, respectively. (B) Zoomed-in view at *g* ≈ 2 (top) and spectral subtraction removing contribution prior to addition of ATP (bottom) showing distinct mar2 cluster signals. Simulated spectra for both components overlaid with indicated *g* values highlight similarity to turnover signals in the FeFe N_2_ase. (C) Reduced and oxidized MarDK EPR spectra showing features characteristic of the oxidized L-cluster. (A) and (B) reproduced from ref 100. CC BY-NC-ND 4.0. (C) reproduced from ref 101. CC BY 4.0.

Upon mixing oxidized MarDK with reduced MarH and ATP, the as-isolated MarH and MarDK signals largely disappear, and small amounts of signals corresponding to MarDK at very reducing potentials appear. Such signals have been interpreted to reflect interprotein electron transfer from MarH to reduce the MarDK clusters, with the low-potential MarDK signals assigned to P-cluster-like states (Figure 30C).^101^ Mechanistic insight has been further interpreted from spectroscopy performed on MarH and MarDK samples frozen while under turnover conditions, again analogous to early studies on N_2_ases (Figure 30A,B).^365,366^ In the presence of ATP, reductant, substrate, and an ATP-regeneration system based on creatine phosphate/creatine phosphokinase, new EPR signals are observed in the *g* ≈ 2 region.^100^ Spectral subtraction of the samples containing ATP from those lacking ATP show two distinct EPR-active *S* = 1/2 species at [*g*_1_, *g*_2_, *g*_3_] = [1.98, 1.91, 1.78] (*g*_iso_ = 1.89) and [*g*_1_, *g*_2_, *g*_3_] = [2.02, 1.97, 1.84] (*g*_iso_ = 1.94) in an approximately 1:1 ratio (Figure 30A,B). These species are notably distinct in line shape and position from standard iron–sulfur cluster signals and resemble the assigned hydride-bound species trapped under similar conditions for the FeFe N_2_ase.^208^ Based on the spectral similarities, these *g* values implicate the formation of purported metal-hydride species at the mar2-cluster active site of MarDK during catalytic C–S bond cleavage. Thus, while the precise mechanism of activity and stoichiometries of protons, electrons, and ATP remain unresolved, a similar coupling between proton transfer and electron transfer to accumulate reducing equivalents on the mar2 cluster appears to be required. Further application of spectroscopy to elucidate the mechanistic details of MAR is needed.

### 8.5 Regulation of MAR

At present, known regulation of MAR occurs at the transcriptional and at post-translational levels. MAR is not synthesized in *Rr* under oxic conditions.^102,327^ During anoxic conditions, expression of MAR is repressed when available sulfate is >100 μM (EC_50‑SO_4__ = 110–140 μM), irrespective of the presence of MAR substrates (MT-EtOH, DMS) or substrate precursors (MTA).^102,119,120,327^ Expression of MAR genes and the additional nearby *nflDK* genes of unknown function in *Rr* require the LysR-type transcriptional regulator, SalR. Notably, SalR protein and mRNA transcript abundance also increases when available sulfate is <100 μM, suggesting SalR is under control of a secondary level of regulation.^102^ Therefore, whether SalR senses the sulfur status of the cell or another master regulator senses the sulfur status of the cell to modulate SalR levels is unknown. Regardless, the expression of MAR in response to available sulfate concentrations falling below 100 μM is consistent with the observation that *Rr* cell growth becomes limited when total available sulfur is ≤100 μM.^327^ Post-translation regulation of MAR activity is controlled by a phenolic acid decarboxylase regulator (PadR) family winged helix protein called MarS.^100^ MarS directly complexes with MarDK under both light-limiting and sulfate-replete conditions to decrease MAR activity by ∼3-fold. Further, interaction of MarS with MarDK leads to higher order structures of ∼500 kDa, reminiscent of PII protein NifI_1_ and NifI_2_ interactions with NifDK in archaea (see section 4.4).^257^ Based on structural modeling, a winged-helix domain from one of the monomers in the MarS dimer is predicted to bind over the surface where MarH binds to competitively inhibit MarH. The higher order structures are predicted to form via each half of the MarS dimer binding two separate MarDK complexes.^100^ Currently it is unknown what regulates binding and release of MarS from MarDK in response to darkness and sulfate availability, but clearly organisms have evolved regulatory mechanisms to control activity of NFL systems that are distinct from use of PII regulators employed for N_2_ase regulation.

## 9 Isethionate Cleavage: Group IV-F ISR

### 9.1 Biological Synthesis of Taurine and Isethionate

Taurine (2-aminoethanesulfonic acid), a precursor of isethionate (2-hydroxyethanesulfonic acid), is an amino sulfonic acid produced by eukaryotic animals, algae, and by some prokaryotes like cyanobacteria *Synechococcus* sp.^367–370^ (Figure 31). In mammals, taurine is synthesized from cysteine by the activity of cysteine dioxygenase (CDO) to form cysteine sulfinic acid, followed by cysteine sulfinic acid decarboxylase (CSAD) to form hypotaurine (aminoethane sulfinic acid). Finally, hypotaurine is oxidized by hypotaurine dehydrogenase to form taurine.^371–373^ While less understood for bacteria, cyanobacterial *Synechococcus* sp. also appear to employ the same biochemical sequence for taurine synthesis using prokaryotic versions of CDO and CSAD.^367,374^ Also, taurine in mammals is alternatively synthesized from Coenzyme A by first degrading Coenzyme A to pantetheine, followed by hydrolysis to pantothenic acid and cysteamine by pantetheinase, and finally oxidation to hypotaurine by CDO.^375^ In algae and birds, a serine/sulfate pathway is used where cysteate is produced as a key intermediate, which is then decarboxylated by CSAD to taurine.^368,369^ Notably in animals, cysteine sulfinic acid can be decarboxylated by either CSAD or l-glutamic acid decarboxylase (GADC), but with an ∼700-fold lower specific activity by GADC.^367,368,376^

**Figure 31:**
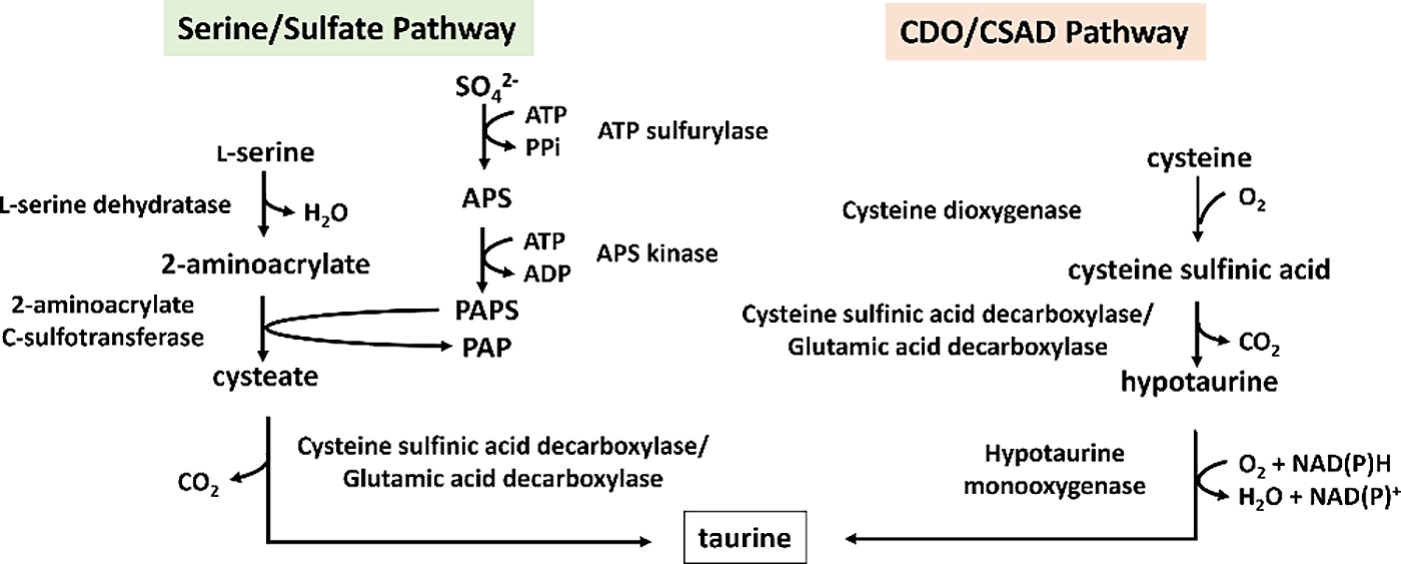
**Taurine synthesis pathways.** APS, adenosine-5′-phosphosulfate; PAPS, 3′-phosphoadenosine-5′-phosphosulfate; CDO, cysteine dioxygenase; CSAD, cysteine sulfinic acid decarboxylase.

Isethionate is produced from taurine in bacteria for sulfur and carbon metabolism by several systems. Taurine-pyruvate aminotransferase (Tpa) transfers the taurine amine onto pyruvate to form alanine and sulfoacetaldehyde as an intermediate. Alternatively, taurine dehydrogenase (TauXY) uses an electron acceptor to oxidize taurine to sulfoacetaldehyde and ammonia.^377–379^ Subsequently, the sulfoacetaldehyde is reduced to isethionate by one of three nonrelated NADPH-dependent sulfoacetaldehyde reductases, SarD, TauF, and IsfD, that thermodynamically favor the reductive reaction toward isethionate under physiological conditions^380,381^ (Figure 32). Also, nonrelated NAD-dependent isethionate dehydrogenases preferentially oxidize isethionate to sulfoacetaldehyde, but whether the reverse reaction is physiologically relevant in isethionate metabolism is unclear.^104,380^

**Figure 32:**
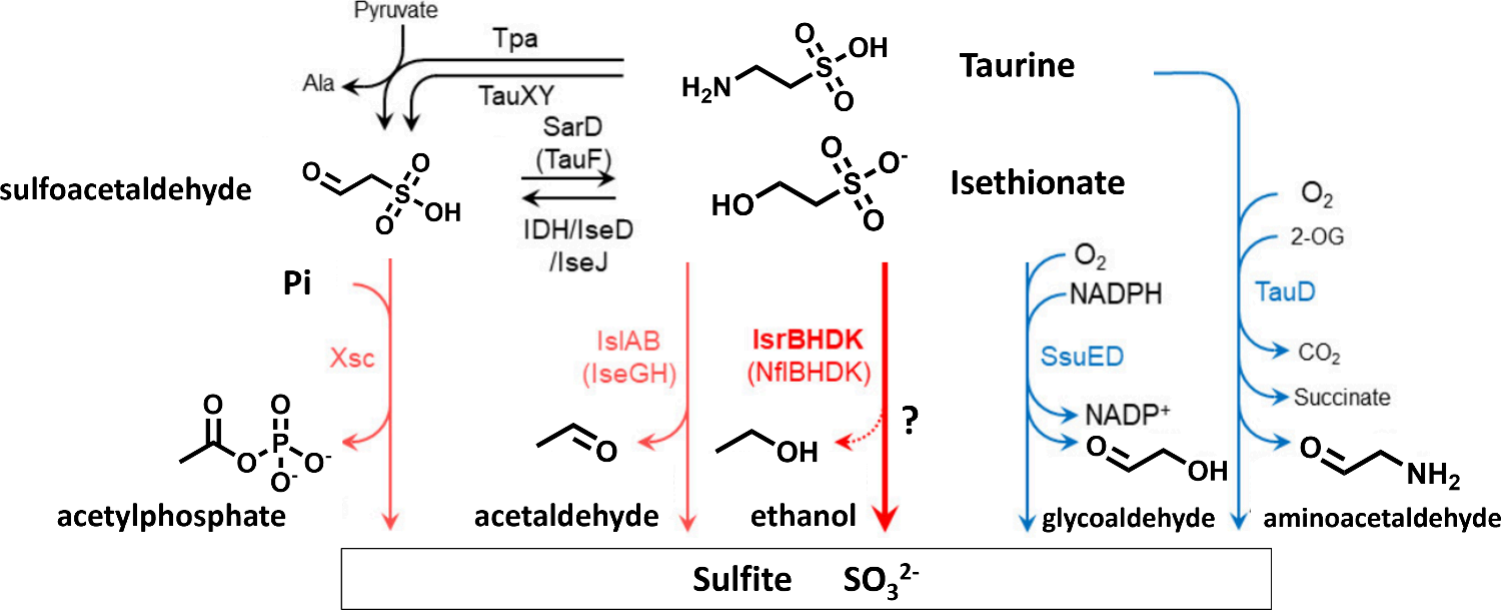
**Taurine and hypoxanthine catabolic pathways.** Tpa, taurine-pyruvate aminotransferase; TauXY, taurine dehydrogenase; SarD, TauF, IsfD, sulfoacetaldehyde dehydrogenase; IseD, IseJ, isethionate dehydrogenase; Xsc, sulfoacetaldehyde acetyltransferase; IslAB, isethionate lyase; Isr, isethionate reductase; SsuED, isethionate monooxygenase; TauD, taurine dioxygenase. Modified from ref 104 to show chemical structures. CC BY 4.0.

### 9.2 Biological Role of Taurine and Isethionate

Marine algae produce taurine and isethionate as an osmoprotectant to regulate cellular pressure and volume in high salinity environments like the ocean.^368,369^ Each year approximately 600–1360 Tg of sulfate is assimilated into organic sulfur compounds by oceanic algae and phytoplankton.^330,382^ Marine microbial sulfur metabolism has resulted in a reservoir of >6700 Tg of dissolved organic sulfur compounds. These organic sulfur compounds including sulfonates like DMSP, taurine and isethionate serve as carbon, sulfur, and energy sources for many oceanic bacteria.^330,383,384^

In mammals, taurine serves many functions and is synthesized by cells at ∼1 mM concentrations, making it one of the most abundant amino acids present in mammalian cells. Taurine serves as an osmolyte to regulate cell shape and osmotic pressure for proper tissue function.^370^ For mitochondria, it has been proposed to serve as a buffer (p*K*_a_ ≈ 9.0) in the mitochondrial matrix, which is the innermost part of the mitochondria, to maintain the proton gradient across the mitochondrial membrane near 0.5 pH units (outside pH ≈ 7.0; matrix pH ≈ 7.5).^385^ Maintaining a ΔpH gradient (i.e., a proton motive force) and a metal ion gradient (Δψ) are necessary components of the membrane ion potential for chemiosmotic production of ATP by ATP synthase.^386,387^ Taurine is essential in the development and function of the visual system, reproductive system, heart and cardiovascular system, immune system, and central nervous system (reviewed in ref 388). Lastly in mammals, taurine is used in the synthesis of taurine-conjugated bile salts. Bile salts, which are amino acid modified cholesterols produced by the gall bladder, aid in fat breakdown and vitamin and drug absorption, and they serve as liver signaling molecules and as antibiotic compounds (reviewed in ref 389). Taurine and isethionate from the body can be taken up by host-associated bacteria to serve as an osmoprotectant.^390^ In addition, taurine, isethionate, and taurine-conjugated bile salts can be used as carbon, sulfur, and energy sources by commensal and pathogenic gut bacteria.^391–393^

Given the prevalence of taurine and isethionate in host and marine ecosystems, bacteria have developed numerous mechanisms for their assimilation as carbon or sulfur sources, or for energy metabolism. For taurine and isethionate catabolic pathways previously characterized, the consistent end result is sulfite and a carbon-containing product (Figure 32).^104,379^ Once sulfite is released from taurine and isethionate, bacteria can then utilize the sulfite for cysteine and methionine synthesis through assimilatory sulfate reduction pathways.^394^ In addition, anaerobic sulfate reducing bacteria (SRB) can perform anaerobic respiration for energy metabolism (oxidative phosphorylation) with sulfite as the terminal electron acceptor.^394–396^

### 9.3 Identification of ISR in Sulfur Metabolism

Recently, a Group IV-F NFL system named isethionate reductase (ISR) was observed to be involved in sulfur assimilation from isethionate in the nonsulfur purple bacterium, *Rhodobacter capsulatus*. This system was composed of gene cluster *isrBHDK*. Notably, *C. sphaeroides*, which does not naturally possess isethionate reductase, was able to grow with isethionate when complemented with *isrBHDK* or the characterized isethionate sulfite-lyase (islA) and activating enzyme (islB) genes.^397,398^ Given that isethionate sulfite-lyase results in the production of sulfite, it was concluded that the product of isethionate cleavage by ISR was sulfite as well. Furthermore, drawing analogy to the reductive nature of N_2_ase and NFL enzymes of known function (Nif, Vnf, Anf, DPOR, COR, CfbCD, MAR), and based on the observation that no ethylene was released from isethionate (2-hydroxyethanesulfonic acid) by ISR, it was proposed that the carbon-containing product of isethionate cleavage by ISR is expected to be ethanol. Specifically, multiple rounds of the presumed IsrH catalytic cycle would ultimately provide at least two electrons in an ATP-dependent manner to IsrDK. The two-electron reductive cleavage of the sulfonate C–S bond between C1 of the 2-hydroxyethane group and the sulfonate S would result in ethanol and sulfite.^104^ However, biochemical confirmation of this reaction and the proposed reductive nature of ISR is still to be determined.

### 9.4 Structure of Isethionate Reductase

The structure of ISR minimally is a heteromeric complex of IsrD and IsrK. Synthesis of a 6-histidine tagged version of IsrD on the N-terminal end of the protein and isolation by immobilized metal affinity chromatography (IMAC) recovered IsrK in complex with IsrD. By analogy to other N_2_ase and NFL systems, this IsrDK complex is likely a heterotetramer.^104^ Based on primary sequence analysis, IsrH, IsrD, and IsrK share 44.1%, 16.8%, and 16.4% sequence identity to NifH, NifD, and NifK from *Azotobacter vinelandii*, respectively. In addition, IsrB shares only 13.8% similarity to NifB of *A. vinelandii*. IsrH possesses the two cysteine residues Cys99 and Cys135 (*R. capsulatus* numbering), corresponding to NifH Cys97 and Cys132 in sequence alignments (*Av* numbering) for likely [4Fe-4S] cluster binding. In addition, IsrH possesses conserved sequences for the Walker A, Walker B, Switch I, and Switch II motifs for ATP binding and hydrolysis and signal transduction between the nucleotide and [4Fe-4S] cluster.^63,102,104^ Whether or not IsrH follows the same catalytic cycle of the NifH Fe protein with hydrolysis of two ATP per electron is unknown.^191–195^ For catalytic metallocofactor coordination, IsrDK conserved cysteine residues are distinct compared to N_2_ase and DPOR/COR systems. Of the six conserved cysteine residues for P-cluster coordination by N_2_ase (Figure 10), IsrDK possesses only cysteine residues corresponding to NifD Cys88 and NifK Cys70, Cys95, Cys153. By analogy to DPOR and COR, as discussed in section 5, these four cysteine residues suggests coordination of a [4Fe-4S] cluster at the IsrDK interface.^104,302^ IsrDK also lacks the metallocofactor coordinating residues Cys270 and H429 found in MarD for binding the L-like mar2 cluster. Further structural determinations are required to elucidate the identity and coordination of the Isethionate Reductase IsrDK metallocofactor.

### 9.5 Regulation of ISR

Expression of *isrBHDK* is linked to sulfur availability for synthesis of needed cellular cysteine and methionine. When *R. capsulatus* was grown on isethionate versus replete sulfate, *isrBHDK* showed a 1350–3000-fold increase in abundance. Coordinately, the proposed sulfonate ABC transporter genes *ssuCAB* increased in abundance 1250–4650-fold. However, it is not known at present whether these genes are regulated in response to the absence of sulfate or presence of isethionate, or both. Notably, other known taurine and sulfur metabolism genes located on the 26 kb genomic region surrounding *isrBHDK* also increased in abundance over 1000-fold when cells were grown on isethionate versus sulfate. These included taurine ABC transporter genes *tauABC*, serine *O*-acetyltransferase (*cysE*) that forms *O*-acetylserine for cysteine synthesis by *O*-acetylserine sulfhydrylase (*cysK*, cysteine synthase), and cysteine desulfurase (*sufS*) for Fe–S cluster synthesis.^104^ Such a large-scale shift in the expression of sulfur metabolism genes in response to *R. capsulatus* growth in isethionate versus sulfate is analogous to responses observed in *Rr* for growth on VOSCs versus sulfate. In *Rr,* numerous genes contained on a 36 kb region of the genome designated as the “thiol cluster” exhibited >50-fold increase in transcript and >200-fold increase in protein abundance during growth on (i) limiting sulfate versus replete sulfate, (ii) growth on MT-EtOH versus replete sulfate, and (iii) growth on 5′-methylthioadenosine (MT-EtOH precursor) versus growth on replete sulfate.^102,119,120^ Upregulated *Rr* thiol cluster genes not only included *marBHDK,* but also multiple homologues of methionine transporters (*metINQ*), homoserine *O*-acetyltransferase (*cysE*) and *O*-acetylhomoserine sulfhydrylase (*OAHS*) for methionine synthesis. The similarity between *R. rubrum* and *R. capsulatus* transcriptional response suggests that *isrBHDK* expression in *R. capsulatus* is regulated by sulfate, as observed for *marBHDK* expression in *R. rubrum* (see section 8.5). Currently the regulatory proteins, specific signals that induce *isrBHDK*, and whether organisms can use isethionate via ISR as a sole carbon source, in addition to a sulfur source, are unknown.

## 10 Conclusions and Outlook

For decades, enigmatic gene sequences that are homologues of N_2_ase have been progressively identified, scattered throughout the genomes of diverse bacteria. Even before the first of these nitrogen fixation-like genes and proteins were functionally characterized, it was evident from their amino acid sequences that the reactions they catalyze are almost certainly different from N_2_ase.^123^ Notably, key N_2_ase residues involved in coordinating the P-cluster and catalytic FeFe-co, FeV-co, or FeFe-co were substituted to other amino acid incapable of such coordinating contacts (Figure 15). Likewise, the quintessential N_2_ase NifD Q191 and H195 were also lacking, which dynamically coordinate binding of the FeMo-co belt sulfur S2B, N_2_ substrate, and likely other substrates to the accessible cofactor iron(s) (Figure 16). Through work in photosynthetic organisms, including bacteria, algae, and plants, the dark-operative protochlorophyllide oxidoreductase (DPOR) was the first NFL system to be isolated, revealing an architecture and catalytic electron transfer paradigm that paralleled N_2_ase. In fact, to date all characterized NFL systems, and even those observed through genome sequencing, are configured as a two-component system for catalytic electron transfer via a series of iron–sulfur cluster metallocofactors.

The highly conserved Fe protein homologues of NifH, also known as “component 2”, share over 40% identity across all analyzed sequences and are poised to initiate electron transfer via a singular [4Fe-4S] cluster at the homodimer interface. Through the structural studies on both N_2_ase and DPOR (Figure 24), the electron transfer appears to be gated through movement of the cluster position relative to the Fe protein surface to modulate its midpoint potential. Such movements are orchestrated by the Switch I and Switch II domains of the Fe protein, based on the phosphorylation state of the bound nucleotide cofactor, likely ATP versus ADP in all cases.^63^ Clearly, further work is required with the recently characterized methylthio-alkane reductase (MAR), isethionate reductase (ISR), and Ni^2+^-sirohydrochlorin *a*,*c*-diamide reductive cyclase (CfbCD), as well as those of unknown function, to determine how the nucleotide identity and hydrolytic cycle tune the protein structure and electron potential to gate component interactions and electron transfer. Embedded in this Fe protein electron transfer mechanism is the intriguing question of specificity. To date there is no coherent understanding of the surface structure of the Fe protein for specific docking to its cognate catalytic component across the nitrogenase superfamily of enzymes. For example, the Fe protein of DPOR has been shown to cross react with the catalytic component of COR, and vice versa. Likewise, the Fe protein of *A. vinelandii* N_2_ase has been shown to cross react with the catalytic component from *K. pneumoniae*, among others. Yet in stark contrast, there is no observation of unmodified MAR, ISR, and DPOR/COR catalytic components cross-reacting with N_2_ase Fe proteins. Even among the N_2_ases themselves, Fe proteins of *C. pasteurianum* and *R. rubrum* do not cross-react with the *A. vinelandii* and/or *K. pneumoniae* catalytic components.^399,400^ Structural, mutagenesis, and modeling work is necessary in the future to understand the mechanisms by which Fe proteins specifically interact with their target protein partners. Such specificity is especially relevant in the context of a biological cell like *R. rubrum*, where N_2_ase and multiple NFL systems must operate concurrently, whereby each carry out their distinct roles in nitrogen, sulfur, and bacteriochlorophyll metabolism in a regulated manner. Certainly, aberrant *in vivo* cross reactions between N_2_ase and NFL components, and between N_2_ase regulatory proteins (e.g., DRAG, DRAT, NifI_1_I_2_) and NFL components that compromise activity would be detrimental, particularly for NFL systems whose abundance is 10–100 times less than N_2_ase. As the NFL field progresses, it is prudent to consider that NFL Fe proteins may not all follow the same catalytic cycle as N_2_ase. The emerging story of CfbCD indicates that the CfbC Fe protein, either when isolated or *in vivo*, forms a persisting stable complex with CfbD, raising the question as to whether component dissociation must occur for catalytic turnover in some NFL systems.^300,311^

At present, one of the greatest challenges facing the field of NFL enzymes is to uncover the function of the numerous systems remaining uncharacterized. Of the six Group IV NFL clades (Figure 8), only Nfa from Group IV-A, CfbCD from Group IV-B, MAR from Group IV-C, and ISR from Group IV-F have a verified or proposed function. Further, Group VI is completely devoid of any characterized members. Many of these groups are deeply rooted, like Group V, where DPOR and COR catalyze separate protochlorophyllide ring reduction reactions. The sequence divergence in Group IV-F alone between ISR and other Group IV-F members already hints that subclades within this group have disparate functions (Figures 10 and 11). Certainly, other Groups will not be monofunctional either. A key barrier to unraveling the biologically relevant functions of NFL systems is the lack of NFL-possessing organisms with tractable genetics. To date, only *R. rubrum*, *R. palustris*, and *R. capsulatus* present the field with amenable systems for studying MAR, ISR, and three additional NFL enzymes of unknown function: two in *R. palustris* and one in *R. rubrum*. Ever since the identification of Group IV and Group VI genes in sequenced genomes, genetic context has been little help for unravelling the mystery of their biologically relevant substrate(s) beyond a tantalizing association of Group IV NFL sequences with genes involved in sulfur metabolism and Group VI NFL sequences with genes involved in tetrapyrrole ring metabolism.^102,121,123^ Recently developed heterologous expression systems such as in *R. rubrum*, *R. capsulatus*, and recent advances in *E. coli* expression systems for N_2_ase and NFL proteins will be paramount for identifying specific reactions they can catalyze and for producing sufficient protein quantities for structural and spectroscopic characterization.^100,101,146,401^ However, heterologous expression systems do not overcome the fundamental issue of uncovering and verifying the biological function of these diverse systems in their native hosts, which is compounded by the issue that many NFL systems, like those from Group VI, are present in organisms with no available culturing method.

An emerging feature of the NFL family of enzymes is their diversity of metallocofactors and their coordination for catalysis. Strikingly, as indicated by structural, spectroscopy, and genetic studies, a complex iron–sulfur cluster with a carbide core likely sits at the heart of the majority of the Group IV NFL enzymes. The N_2_ase NifB, which synthesizes the initial L-cluster (also known as NifB-co), and MarB, whose precise metallocofactor product is unknown, both can assemble and insert a functional metallocofactor into MAR.^100^ The fundamental question needing answered is whether NifB, MarB and other NFL homologues (NflB) synthesize the same initial cofactor. Given the radical SAM and additional SAM binding domains are conserved across NifB and Group IV NflB homologues, except for those associated with ISR, a central carbide is tenable. However, whether the NflB homologues insert a ninth sulfur to make the full [9S-8Fe-C] L-cluster or stop at the precursory [8S-8Fe-C] L*-cluster remains to be determined.^67^ Further, given the NifB homologue, IsrB, from isethionate reductase only possesses a radical SAM domain and IsrDK is lacking conserved residues for FeMo-co or L-cluster binding, its function at present remains obscure. A final tantalizing metallocofactor question for the NFL systems yet to be characterized is the presence of a heterometal. Recent work with the *Roseiflexus* sp. N_2_ase system, which lacks NifEN, has 50-fold higher activities when synthesized in the presence of Mo, suggesting N_2_ase and NFL enzymes alike may be able to form a Mo-substituted catalytic cofactor *in situ* to achieve needed substrate specificities.^131^ However, given the numerous groups of NFL sequences with conserved histidine and cysteine residue pairs for coordinating an L-like cluster (Figure 11), it is unlikely that a unique catalytic metallocofactor occurs for each of these groups as the defining factor in substrate specificity. Significant endeavors in structural biology, amino acid substitutions, and advances in trapping bound intermediates and inhibitors are necessary to determine (i) how the protein environment surrounding the catalytic metallocofactor confers substrate specificity and (ii) identify whether the cofactor directly coordinates the substrate during catalysis.

In closing, NFL enzymes are ultimately biological catalysts, and as such need regulatory mechanisms to ensure proper cellular operation. A hallmark of N_2_ase when it composes 10% or more of the cellular proteome to fix sufficient N_2_ for the organisms’ metabolism is its energy demand in the form of ATP and low potential electrons. As such, Fe protein post-translational modification proteins (DRAG and DRAT) for ADP-ribosylation and PII-family proteins that bind the catalytic subunit all occur to prevent association of the two N_2_ase component for catalysis when cellular energy is depleted or fixed nitrogen becomes sufficient. Even in the NFL enzymes, *R. rubrum* has found the need to inactivate MAR through binding of a winged-helix protein, MarS, when cellular energy is depleted or available sulfur becomes sufficient.^100^ Further, given the sensitivity of N_2_ase to oxidative damage by O_2_ and noncompetitive inhibition by CO, regulatory proteins have evolved that bind and protect N_2_ase from these detrimental molecules. Specifically, the Shethna II protein embeds itself between NifH and NifDK to shut down N_2_ase activity and protect the metallocofactors from oxidative damage by O_2_,^402–404^ and the CowN protein complexes with NifDK to selectively block CO from accessing the active site.^405–407^ While the colocalization of genes for regulatory proteins with N_2_ase and NFL genes may implicate their role as such, genomic context alone provides little for surmising the biochemical regulatory switch. Also, as in the case of *R. rubrum* MarS and *A. vinelandii* Shethna II protein, the corresponding genes are located elsewhere on the genome, and their role became evident only after being surprisingly copurified with the catalytic subunits. Undoubtedly, new regulatory proteins and mechanisms that modulate or protect NFL activity are awaiting to be discovered along with the functions of the NFL enzymes themselves.
